# Supplementary material for: METTL1 Promotes let-7 MicroRNA Processing via m7G Methylation
Source: Mol Cell. 2019 Jun 20;74(6):1278–1290.e9. doi: 10.1016/j.molcel.2019.03.040 (PMC6591002; doi:10.1016/j.molcel.2019.03.040)
Supplement: Document S1. Figures S1–S6, Table S8, and Methods S1 [file mmc1.pdf]

Molecular Cell, Volume *74*

Supplemental Information

METTL1 Promotes *let-7* MicroRNA Processing via m7G Methylation

Luca Pandolfini, Isaia Barbieri, Andrew J. Bannister, Alan Hendrick, Byron Andrews, Natalie Webster, Pierre Murat, Pia Mach, Rossella Brandi, Samuel C. Robson, Valentina Migliori, Andrej Alendar, Mara d'Onofrio, Shankar Balasubramanian, and Tony Kouzarides

Figure S1

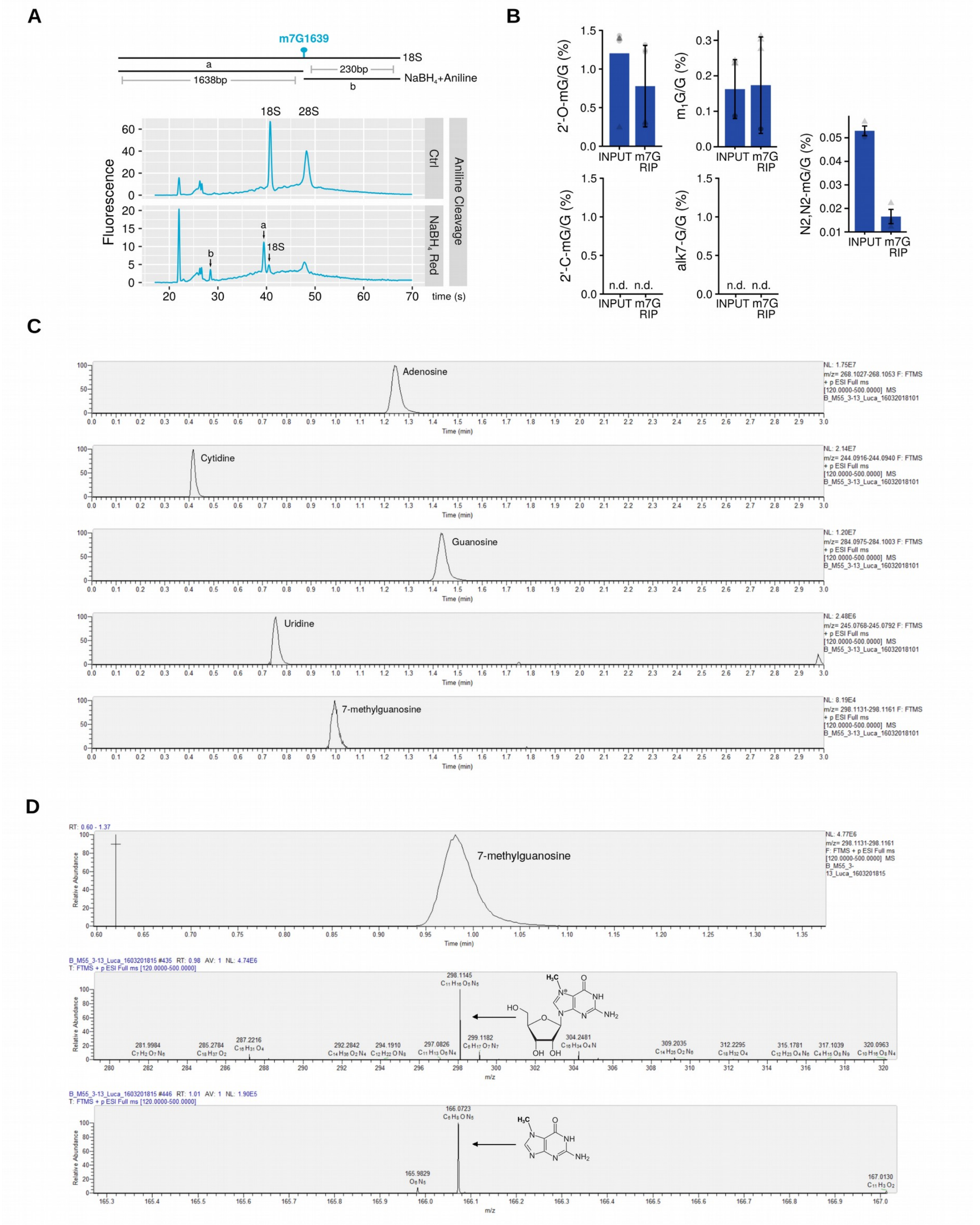

Figure S1 | High-throughput profiling of m7G modification in miRNAs. Related to Figure 1.

A, Schematic representation (top) of the expected cleavage of *18S* rRNA after $NaBH_4$ + aniline treatment and electropherogram analysis (bottom) of untreated and treated total RNA from A549 cells. **B,** Immunoprecipitation with anti-m7G antibody does not enrich RNAs containing five different guanine modifications (including four methylations) as determined by mass spectroscopy. Error bars represent S.E.M. **C,** HPLC trace of the four canonical RNA nucleosides along with 7-methylguanosine, showing retention times that are an exact match with pure nucleosides run as quantitation standards. **D,** Extracted ion chromatogram of 7-methylguanosine, with the accompanying mass spectra showing the intact protonated precursor ion of the nucleoside [M+H] (middle panel) and the 7-methylguanine ion generated by in-source fragmentation as an internal verification of its identity.

Figure S2

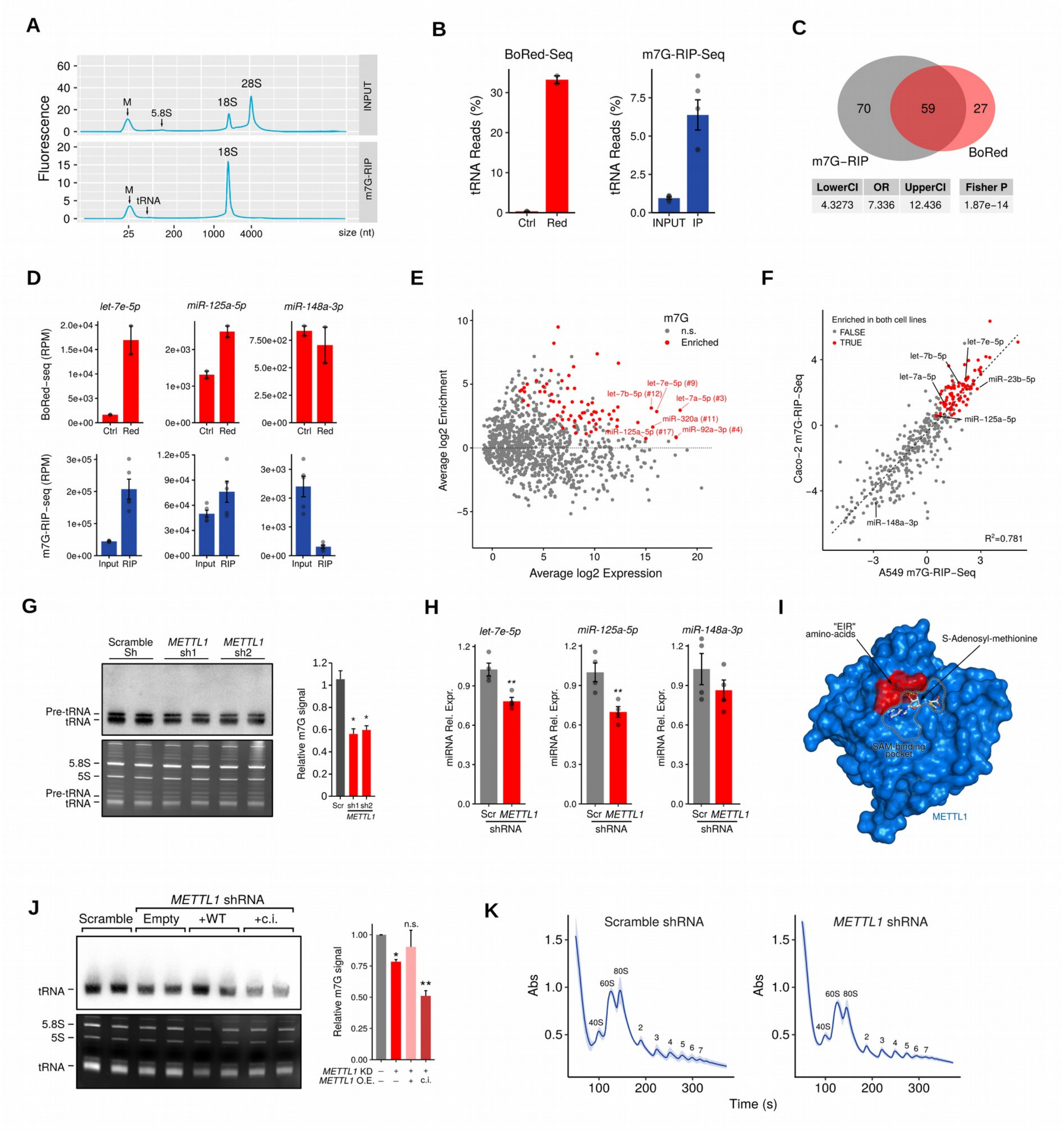

Figure S2 | Validation of BoRed-Seq and m7G-RIP-Seq. Related to Figure 1.
A, Electropherogram analyses of total RNA before and after immunoprecipitation with an m7G-specific antibody showing specific enrichment for *18S* rRNA in the immunoprecipitate. **B,** Read counts for tRNAs from the BoRed-Seq and m7G-RIP-Seq experiments are shown. Error bars represent S.E.M. **C,** Venn diagram showing the high overlap between miRNAs significantly enriched in either m7G-RIP-Seq or BoRed-Seq of A549 cells, respectively. Confidence interval of the odds ratio (OR) and P-value, as obtained by Fisher's exact test, are indicated in the table below. **D,** Read counts for the indicated miRNA from the BoRed-Seq and RIP-Seq experiments are shown. Error bars represent S.E.M. **E,** Scatter plot showing the absence of correlation between m7G and miRNA expression levels. Labels show the m7G containing miRNAs amongst the top 20 most expressed ones and their absolute ranking. **F,** Scatter plot of m7G-RIP-Seq miRNA enrichment in Caco-2 versus A549 cells, highlighting a high degree of correlation, as indicated by R^2 Pearson correlation coefficient. **G,** Immunoblot using an m7G-specific antibody shows a reduction of m7G levels in tRNAs upon *METTL1* knockdown. Right graph shows the average ± S.E.M. digital quantification of the m7G signal (*P<0.05, two tailed t-test). **H,** RT-qPCR quantification of *let-7e-5p, miR-125a-5p* and *miR-148a-3p* upon *METTL1* KD in Caco-2 cells. The average of four independent biological replicates ± S.D. is shown (**P<0.01, two tailed t-test). **I,** Crystal structure of METTL1 in complex with S-adenosylmethionine (PDB file: 3CKK). The EIR (107-109) amino acids mutated to alanine to generate the catalytically inactive mutant are shown in red. **J,** RNA immunoblot with an m7G-specific antibody on total RNA from *METTL1* knockdown or control cells over-expressing either WT METTL1 or a catalytically inactive METTL1 (c.i.). **K,** Polysome fractionation analysis. Cell extracts from control or *METTL1* knockdown cells were prepared and resolved in a 5–50% sucrose gradient. The absorbance at 254 nm was continuously measured. The peaks corresponding to free 40S and 60S subunits, 80S and polysomes are indicated. Shaded area in grey indicates the S.E.M. of two independent samples for each condition.

Figure S3

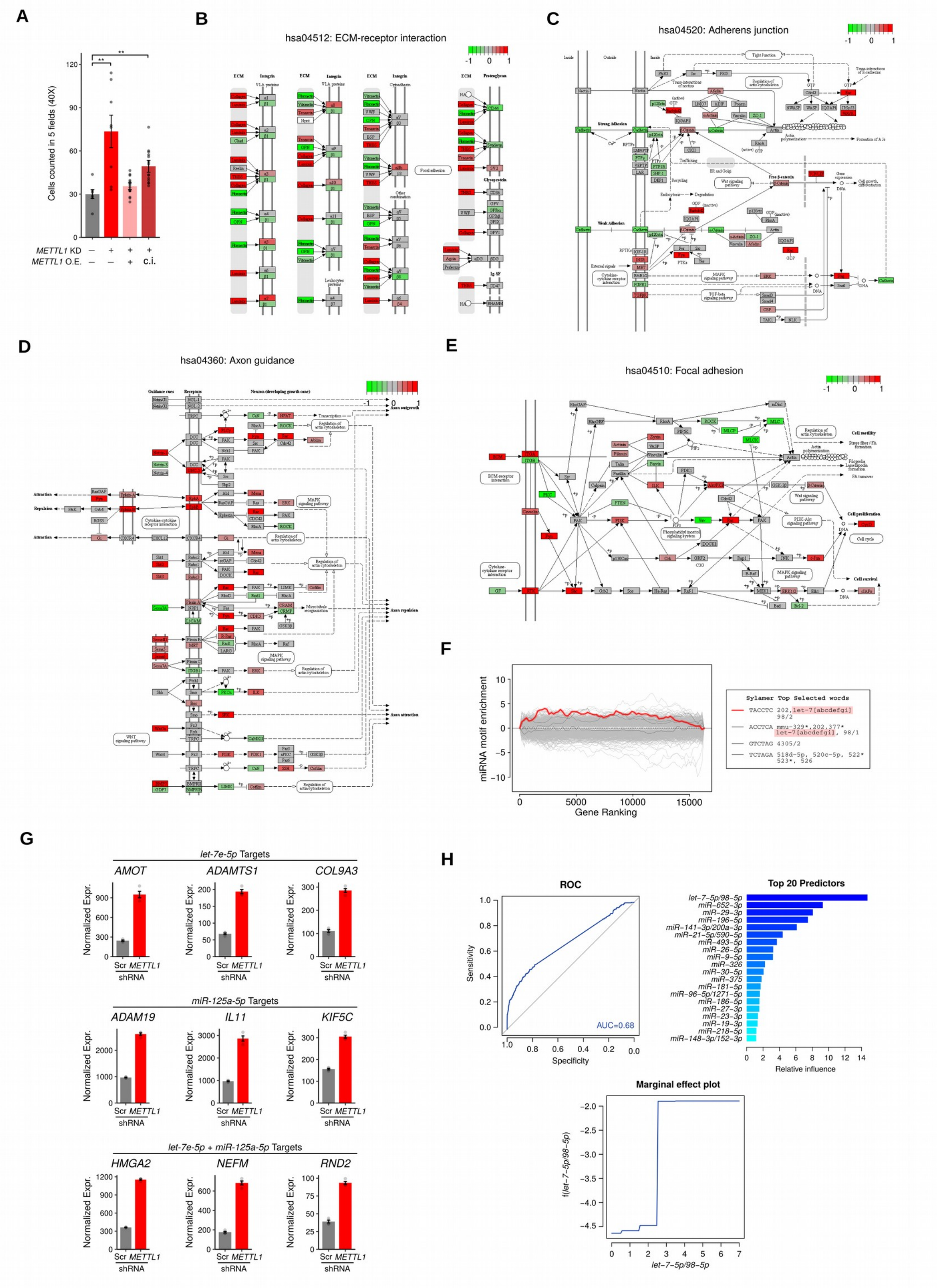

Figure S3 | Effects of targeting *METTL1* in A549 cells. Related to Figure 2.
A, migration assay of A549 cells. Increased migration due to *METTL1* KD is rescued by the expression of WT METTL1 (+), but not by a catalytically inactive version of the enzyme (c.i.). **B,C,D,E,** Graphical representation of KEGG pathway regulation, showing up-regulation of ECM-receptor interaction (B), adherens junction (C), axon guidance (D) and focal adhesion (E). Data were obtained by comparing RNA expression data from *METTL1* KD and control A549 cells (up-regulated genes, red; down-regulated genes, green). **F**, Sylamer miRNA target seed motif analysis on up-regulated mRNAs upon *METTL1* KD, showing a specific enrichment for the *let-7 (5p)* seed motif. **G,** representative cell migration genes whose expression is upregulated upon *METTL1* KD, which are targets of *let-7e-5p*, *miR-125a-5p* or both (for all of them FDR<0.05, as evaluated with *limma*). **H,** A gradient boosting classifier was used to model the mRNA expression fold changes observed by microarray analysis as a function of the presence of seed sites for different miRNAs in 3'-UTRs. Receiver operating characteristic (ROC) curve displays the goodness of the model, with an area under ROC (AUC) of 0.68. The presence of *let-7 (5p)* seed sequence within an mRNA represents the strongest predictive factor for its up-regulation upon *METTL1* KD, as shown by the top 20 predictor list. The marginal effect plot shows that there is a positive correlation between the number of seeds in the 3'-UTR and mRNA up-regulation.

Figure S4

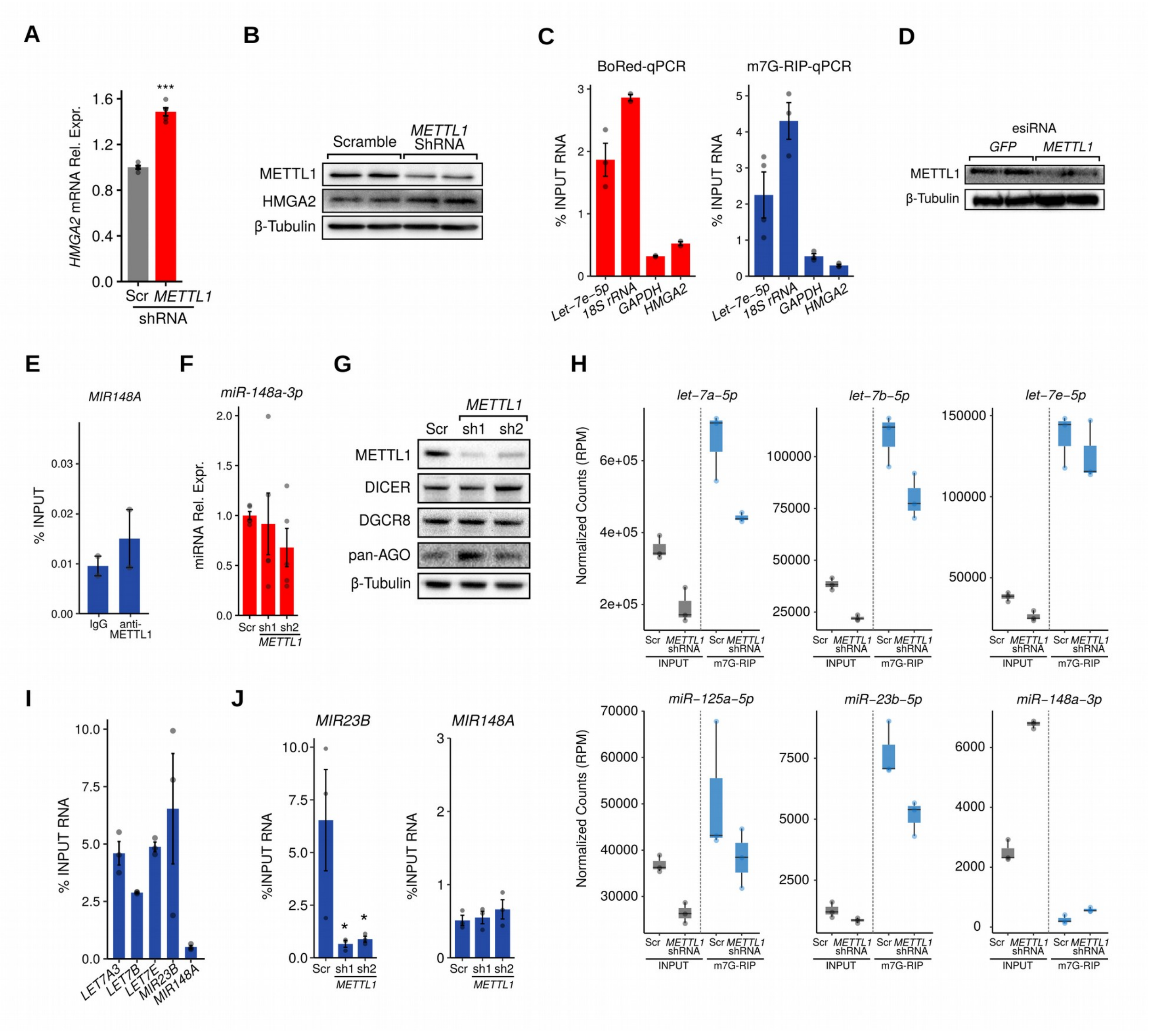

Figure S4 | miRNA processing is dependent on m7G. Related to Figure 3 and 4.
A, *HMGA2* expression measured by RT-qPCR in Caco-2 cells infected with *METTL1*-specific or control (Scr) TET-inducible shRNAs five days after doxycycline treatment. The average of five biological replicates ± S.D. is shown (***p<0.001, two tailed t-test). **B,** Western blot showing METTL1, HMGA2 and β-tubulin protein levels in Caco-2 cells infected with *METTL1*-specific or control (Scramble) TET-inducible shRNAs five days after doxycycline treatment. Two representative biological replicates of a total of four are shown. **C,** BoRed and m7G-RIP coupled to RT-qPCR of *HMGA2* in A549 cells. *18S* rRNA is used as a positive control; *GAPDH* is a negative control. *let-7e-5p* levels are replotted alongside as a positive reference. The average of three biological and technical replicates ± S.E.M. are shown. **D,** Western blot showing METTL1 down-regulation upon transfection with *METTL1* specific siRNAs in A549 cells stably expressing a luciferase cDNA with a *Hmga2*-3'-UTR with mutated *let-7* seed sites. Two independent transfections of a total of four replicates are shown. **E,** CLIP-qPCR using a METTL1-specific antibody or a non-specific IgG. The levels of immunoprecipitated *miR-148a* primary hairpin are shown. The average of two independent immunoprecipitation reactions ± S.E.M. is presented (*P<0.05, two tailed t-test). **F,** RT-qPCR showing the levels of *miR-148a-3p* upon *METTL1* KD in A549 cells. The average of five independent biological replicates ± S.D. is shown. **G,** Western blot of DICER, DGCR8 and ARGONAUTE (pan-AGO) upon *METTL1* KD in A549 cells. **H,** Box plots visualizing the normalized smallRNA-Seq counts of INPUT and m7G-RIP miRNAs from either control (Scr) or *METTL1* KD A549 cells. **I,** m7G RNA immunoprecipitation and RT-qPCR of *LET7A3*, *LET7B*, *LET7E/125A* and *MIR23B* primary transcripts in A549 cells. *MIR148A* is shown as a negative control. **J,** m7G-RIP and RT-qPCR of *MIR23B* and *MIR148A* primary transcripts upon *METTL1* KD in A549 cells. The average of three independent biological replicates ± S.E.M. is shown (*P<0.05, **P<0.01, ***P<0.001, two tailed t-test).

Figure S5

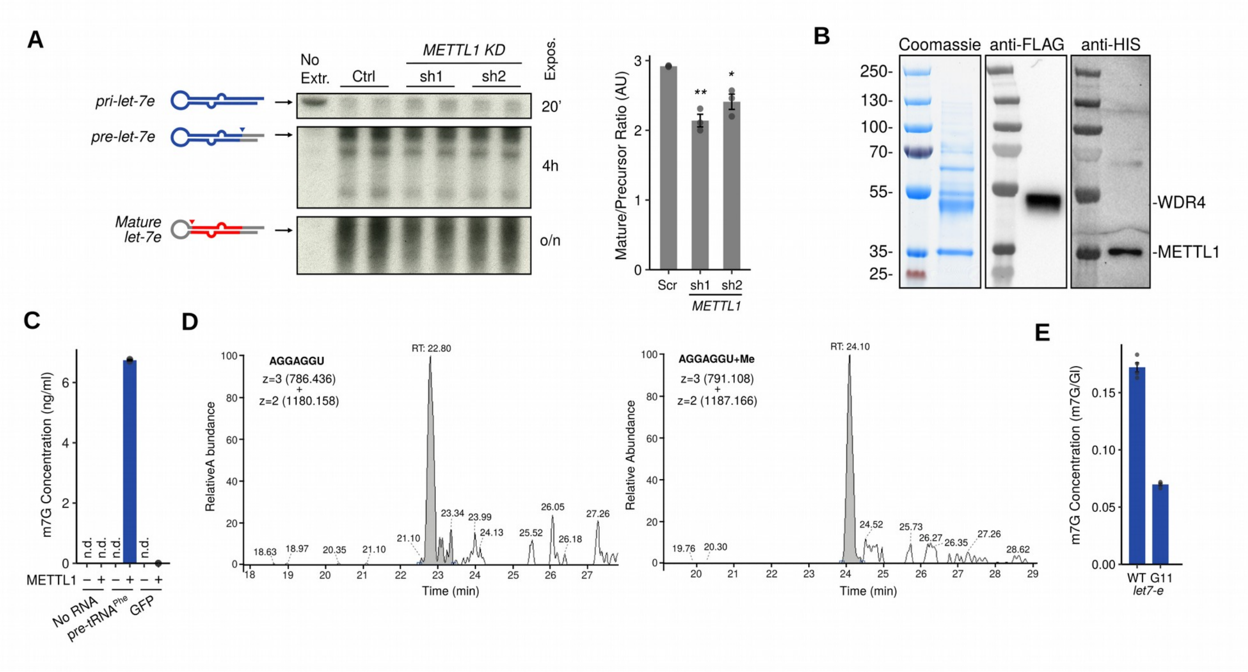

Figure S5 | METTL1-mediated miRNA methylation. Related to Figure 4 and 5.
A, Autoradiography and digital quantification of the results from *in vitro* processing of a radioactively labelled *pri-let-7e* incubated with cell extracts from control (Ctrl) or *METTL1* KD A549 cells. The average of three independent experiments ± S.D. is shown (*P<0.05, **P<0.01, two tailed t-test). Autoradiography images are composite of different molecular weight regions and exposure times. Full, unprocessed images are deposited on Mendeley Data. **B,** Coomassie staining and Western blot of co-expressed recombinant 6xHis-tagged METTL1 and FLAG-tagged WDR4 from baculovirus-infected insect cells. **C,** *In vitro* methylation reaction using recombinant METTL1/WDR4 pre-assembled complex on pre-tRNA^{Phe} or a hairpin shRNA targeting GFP (negative control). Mass spectrometry analysis shows specific m7G methylation of pre-tRNA^{Phe}. **D,E,** Representative extracted ion chromatograms of collated charge states of unmodified (left chromatogram) and methylated AGGAGGU (right chromatogram) oligonucleotides. Precursor ion areas were quantified from technical triplicates (quantified area shown in grey). Chromatograms shown are from a single replicate. **E,** *in vitro* methylation reaction using recombinant METTL1/WDR4 pre-assembled complex on WT and G11-DAG *let-7* oligonucleotides.

Figure S6

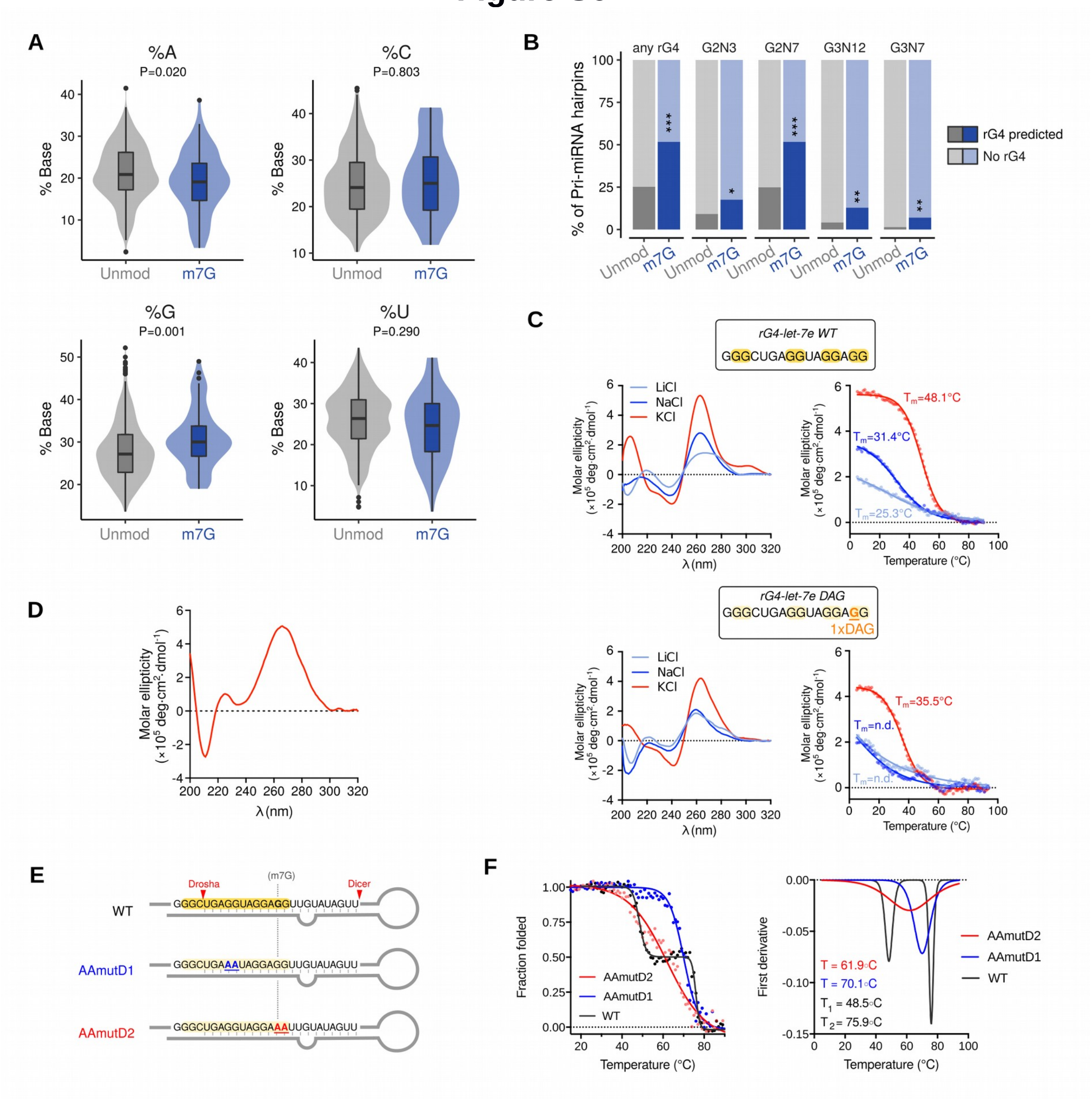

Figure S6 | m7G modified miRNAs are prone to G-quadruplex formation. Related to Figure 5.
A, Box plot showing the base content in primary hairpins of either unmodified or m7G containing miRNAs (P-values are calculated with Wilcoxon test). **B,** Proportion of G-quadruplex forming motifs detected within primary hairpins of either unmodified or m7G containing miRNAs (see STAR Methods for details; *P<0.05, **P<0.01, ***P<0.001; Fisher's exact test). **C,** Circular dichroism (CD) of the minimal *rG4-let-7e* sequence reveals that its structure is cation-dependent and its spectra display a minimum and maximum at 240 and 263 nm, respectively. These are feature of G-quadruplex formation. The stability of *rG4-let-7e* WT structure is cation-dependent, with a thermal stability (T_m) of 48.1 °C in the presence of 100 mM KCl. While G11 to DAG substitution in *rG4-let-7e DAG* does not completely abolishes rG4 formation, it greatly destabilizes it. The thermal stability decreases from 48.1 °C to 35.5 °C in the presence of 100 mM KCl and the oligonucleotide is no longer folded in the presence of LiCl or NaCl. **D,** CD spectra of *pri-let-7e* hairpin. The minimum at 210 nm indicates the expected stem-loop structure. **E,** Cartoon depicting the pri-miRNA hairpins employed in the following panel. **F,** Thermal denaturation studies of GG to AA mutants at the D1 or D2 position, supporting the formation of a G-quadruplex within *pri-let-7e*. Melting profiles of both mutants display a single transition at high temperature characteristic of the canonical hairpin structure. The transition attributed to the rG4 motif is no longer observed. Both mutations destabilize the hairpin structure as expected (decreased GC content).

Table S8

shRNA sequences	
***METTL1* sh1**	CCGGGATGACCCAAAGGATAAGAAACTCGAGTTTCTTATCCTTTGGGTCATCTTTTTG
***METTL1* sh2**	CCGGCCCACATTTCAAGCGGACAAACTCGAGTTTGTCCGCTTGAAATGTGGGTTTTTG
**Scramble shRNA**	CCGGCAACAAGATGAAGAGCACCAACTCGAGTTGGTGCTCTTCATCTTGTTGTTTTTG

Cloning Primers		
***METTL1* cDNA**	**Fw**	GTAGGCGGCCGCACCATGGCAGCCGAGACTCGGA
	**Rev**	CACTGTCTAGATCAGTGACCAGGCAGGCTGG

Gene Synthesis sequences	
***METTL1* WT**	CACACTGGCGGCCGCACCATGGCAGCCGAGACTCGGAACGTGGCCGGAGCAGAGGCCCCACCGCCCCAGAAGCGCTACTACCGGCAACGTGCTCACTCCAACCC<br>CATGGCGGACCACACGCTGCGCTACCCTGTGAAGCCAGAGGAGATGGACTGGTCTGAGCTATACCCAGAGTTCTTCGCTCCACTCACTCAAAATCAGAGCCACG<br>ACGATCCTAAAGACAAAAAAGAAAAGAGAGCTCAGGCCCAAGTGGAGTTTGCAGACATAGGCTGTGGCTATGGTGGCCTGTTAGTGGAACTGTCACCGCTGTTC<br>CCAGACACACTTATTCTGGGTCTGGAGATCCGGGTGAAGGTCTCAGACTATGTACAAGACCGGATTCGGGCCCTACGCGCAGCTCCTGCAGGTGGCTTCCAGAA<br>CATCGCCTGTCTCCGTAGCAATGCCATGAAGCACCTTCCTAACTTCTTCTACAAGGGCCAGCTGACAAAGATGTTCTTCCTCTTCCCCGATCCCCACTTTAAAC<br>GCACAAAGCACAAGTGGCGAATCATCAGTCCCACCCTGCTAGCAGAAT
***METTL1* EIR/AAA Mut**	CACACTGGCGGCCGCACCATGGCAGCCGAGACTCGGAACGTGGCCGGAGCAGAGGCCCCACCGCCCCAGAAGCGCTACTACCGGCAACGTGCTCACTCCAACCC<br>CATGGCGGACCACACGCTGCGCTACCCTGTGAAGCCAGAGGAGATGGACTGGTCTGAGCTATACCCAGAGTTCTTCGCTCCACTCACTCAAAATCAGAGCCACG<br>ACGATCCTAAAGACAAAAAAGAAAAGAGAGCTCAGGCCCAAGTGGAGTTTGCAGACATAGGCTGTGGCTATGGTGGCCTGTTAGTGGAACTGTCACCGCTGTTC<br>CCAGACACACTTATTCTGGGTCTGGCGGCAGCCGTGAAGGTCTCAGACTATGTACAAGACCGGATTCGGGCCCTACGCGCAGCTCCTGCAGGTGGCTTCCAGAA<br>CATCGCCTGTCTCCGTAGCAATGCCATGAAGCACCTTCCTAACTTCTTCTACAAGGGCCAGCTGACAAAGATGTTCTTCCTCTTCCCCGATCCCCACTTTAAAC<br>GCACAAAGCACAAGTGGCGAATCATCAGTCCCACCCTGCTAGCAGAAT

qPCR Taqman Primers			
**Primer**		**Sequence**	**Universal ProbeLibrary Number (Roche Cat# 4683633001)**
GAPDH	**Fw**	TCCACTGGCGTCTTCACC	45
	**Rv**	GGCAGAGATGATGACCCTTTT	
HMGA2	**Fw**	ACCATTTCTGCAAGTTAGGTATGTT	2
	**Rv**	GAACAGGGAGAAAGTCAACTGC	
IL11	**Fw**	GGTGCATCTGTGGATAGAACG	28
	**Rv**	TCCTTAGCCTCCCTGAATGA	

qPCR Sybr Green Primers		
**Primer**		**Sequence**
LET7A3	**Fw**	GGGTGAGGTAGTAGGTTGTATAG
	**Rv**	TATAGTTATCCCATAGCAGGGCAGA
LET7B	**Fw**	CGGGGTGAGGTAGTAGGTTGT
	**Rv**	CAGGGAAGGCAGTAGGTTGTATAG
LET7E/125A	**Fw**	GGGCTGAGGTAGGAGGTTGT
	**Rv**	GGGAAAGCTAGGAGGCCGTA
MIR23B	**Fw**	TGGCATGCTGATTTGTGACT
	**Rv**	GTGGTAATCCCTGGCAATGT
MIR148A	**Fw**	GGCAAAGTTCTGAGACACTCC
	**Rv**	TTCTGTAGTGCACTGACTTCTATCA
pre-let-7e	**Fw**	TGTATAGTTGAGGAGGACACCC
	**Rv**	GCTAGGAGGCCGTATAGTGA
pre-miR-125a	**Fw**	TCCCTGAGACCCTTTAACCT
	**Rv**	CAAGAACCTCACCTGTGACC
pre-miR-148a	**Fw**	GGCAAAGTTCTGAGACACTCC
	**Rv**	TTCTGTAGTGCACTGACTTCTATCA
RNY1	**Fw**	GCTGGTCCGAAGGTAGTGAG
	**Rv**	GGGGGAAAGAGTAGAACAAGG

qPCR Taqman Advanced miRNA Assay Ids (Thermo Fisher Cat# A25576)	
hsa-let-7e-5p	478579_mir
hsa-miR-125a-5p	477884_mir
hsa-miR-196a-5p	478230_mir
hsa-miR-375	478074_mir
hsa-miR-148a-3p	477814_mir
hsa-miR-381-3p	477816_mir
hsa-mir-101-3p	477863_mir

RNA Oligonucleotides	
pre-Phe-tRNA	GCGGAUUUAGCUCAGUUGGGAGAGCGCCAGACUGAAGAAAAAACUUCGGUCAAGUUAUCUGGAGGUCCUGUGUUCGAUCCACAGAAUUCGCACCA
cel-miR-67	CGUCGAUCCGCUCAUUCUGCCGGUUGUUAUGCUAUUAUCAGAUUAAGCAUCACAACCUCCUAGAAAGAGUAGAUCGAUU
pri-let-7e WT	CCCGGGCUGAGGUAGGAGGUUGUAUAGUUGAGGAGGACACCCAAGGAGAUCACUAUACGGCCUCCUAGCUUUCCCCAGG
pri-let-7e AAmutD1	CCCGGGCUGAAAUAGGAGGUUGUAUAGUUGAGGAGGACACCCAAGGAGAUCACUAUACGGCCUCCUAGCUUUCCCCAGG
pri-let-7e AAmutD2	CCCGGGCUGAGGUAGGAAAUUGUAUAGUUGAGGAGGACACCCAAGGAGAUCACUAUACGGCCUCCUAGCUUUCCCCAGG
pri-let-7e D1 (*=DAG)	CCCGGGCUGA**UAGGAGGUUGUAUAGUUGAGGAGGACACCCAAGGAGAUCACUAUACGGCCUCCUAGCUUUCCCCAGG
pri-let-7e D2 (*=DAG)	CCCGGGCUGAGGUAGGA**UUGUAUAGUUGAGGAGGACACCCAAGGAGAUCACUAUACGGCCUCCUAGCUUUCCCCAGG
pri-let-7e G11 (*=DAG)	CCCGGGCUGAGGUAGGA*GUUGUAUAGUUGAGGAGGACACCCAAGGAGAUCACUAUACGGCCUCCUAGCUUUCCCCAGG
rG4-let-7e WT	GGGCUGAGGUAGGAGG
rG4-let-7e DAG (*=DAG)	GGGCUGAGGUAGGA*G

Table S8 | DNA and RNA oligonucleotides employed in this study. Related to STAR Methods

Methods S1 – m7G Profiling Protocol

Related to STAR Methods

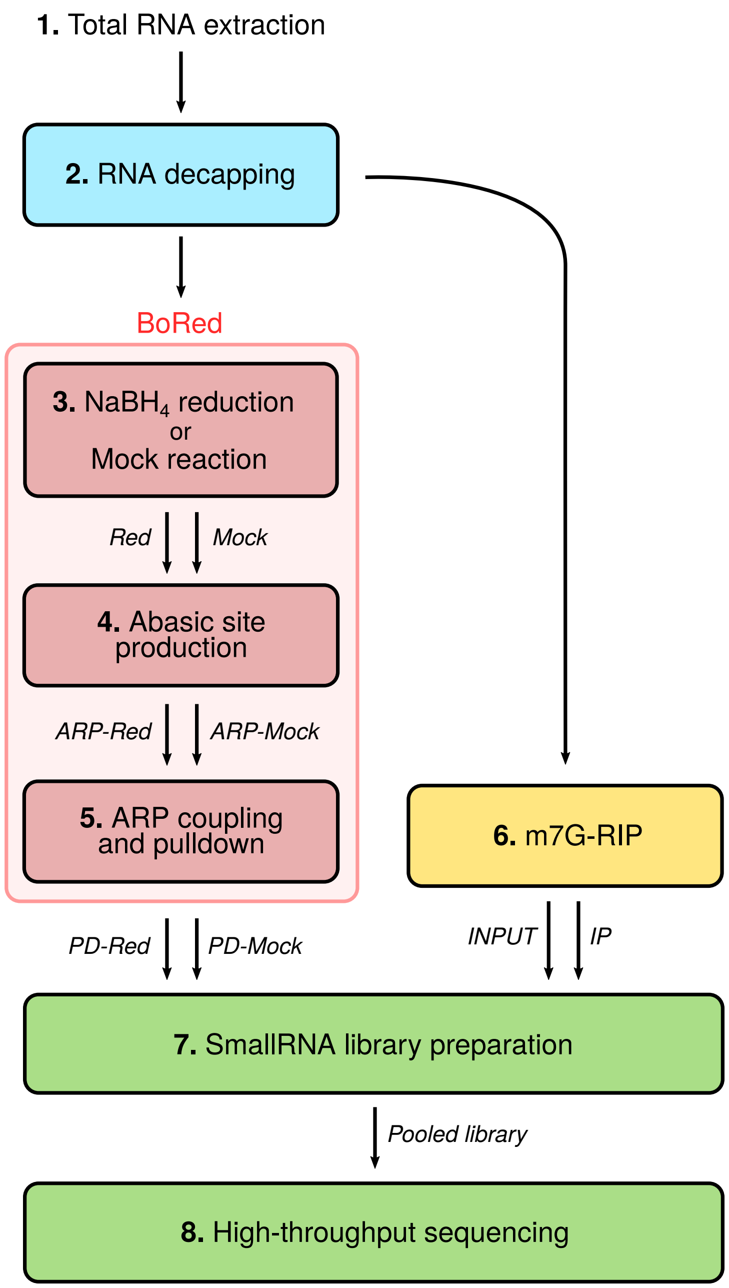

BoRed-Seq Protocol

1 RNA extraction from A549 cells

Total RNA, including miRNAs, is extracted from mammalian cells. To ensure optimal results, it is advisable to always start with freshly extracted RNA.

1. Culture up to 1.5×10^7 A549 cells to a confluency of ~80%.
2. Remove the medium and rinse the cells twice in PBS.
3. Extract RNA using the miRNeasy Mini Kit (Qiagen). Elute RNA in 30 µl RNAse-free water.

2 RNA decapping

Cap-Clip Acid Pyrophosphatase is used to remove the m7GpppG cap of eukaryotic mRNA in order to avoid cross-reactivity to non-internal m7G.

1. Prepare the RNA decapping mix according to Table 1.
2. Incubate for 1 h at 37 °C.
3. Purify RNA using the RNA Clean and Concentrator - 25 Kit (Zymo Research).

Table 1: Reaction mix for RNA decapping for ~20µg total RNA

Volume	Reagent
20 µg	RNA
2 µl	10× Reaction buffer
1 µl	CapCLIP (5 U; CellScript)
1 µl	RNAsin Plus (Thermo Fisher)
20 µl	Final vol. with ddH_2O

3 $NaBH_4$ Reduction

m7G residues are specifically reduced by treatment with $NaBH_4$ in the presence of high concentrations of free m7GTP. Control samples are treated with acid-quenched $NaBH_4$. The $NaBH_4$ treatment also reduces aldehydes in RNA (e.g. 5-formylcytosine or 5-formyluracil), thereby preventing unwanted covalent binding of ARP with them in the downstream stages.

1. Throughout the entire protocol, always use nuclease-free not DEPC-treated water (Ambion) as even small traces of DEPC lead to RNA degradation after $NaBH_4$ reduction.
2. Prepare 3 M Tris-HCl (pH 8.2) and 1 M sodium acetate (NaOAc; pH 4.5). Filter through 0.22 µm filter and autoclave. Store aliquots at -20 °C.
3. Prepare the RNA mix according to Table 2. For each sample prepare n×2 reaction mixes: one reduction reaction (RED) and one control reaction (CTRL). At least three parallel reactions are recommended to obtain sufficient reduced RNA for subsequent steps.
4. Prepare a fresh 1 M sodium borohydride solution ($NaBH_4$; Sigma-Aldrich) immediately before use. The $NaBH_4$ powder should be stored at 4 °C in a desiccator.

Table 2: Reaction mix for 4 µg of RNA

Volume	Reagent
up to 4 µg	RNA
3 µl	37 mM m7GTP (Sigma-Aldrich)
5 µl	3 M Tris-HCl (pH 8.2)
15 µl	Final vol. with ddH_2O

5. Add 15 µl 1 M $NaBH_4$ solution to the reduction reaction. Vortex and spin briefly. Do not add $NaBH_4$ to the CTRL reaction and keep it on ice until quenching.
6. Incubate on ice for 40 min. Open the lid of the tube, debubble and spin down every 10 min.
7. Meanwhile prepare the quenched $NaBH_4$ solution (QB1) for the control reaction (non-reduced RNA) according to Table 3.
8. Incubate QB1 at 90 °C for 30 min.
9. Place QB1 on ice and add 1 µl of glycogen (20 µg/µl; Roche).

Table 3: Preparation of QB1 solution

Volume	Reagent
180 µl	1 M NaOAc (pH 4.5)
120 µl	ddH_2O
15 µl	1 M $NaBH_4$

Table 4: Preparation of QB2 solution

Volume	Reagent
180 µl	1 M NaOAc (pH 4.5)
120 µl	ddH_2O
1 µl	20µg/µl glycogen

10. Prepare the quenching solution for the reduction reaction (QB2) according to Table 4. Keep it on ice until used.
11. After the incubation of the reduction reaction (RED) on ice for 40 min, add 300 µl of QB2 quenching solution drop-wise, to avoid a violent reaction.
12. Add 990 µl of ice-cold absolute ethanol.
13. Add 315 µl of QB1 solution and 990 µl of ice-cold absolute ethanol to the control reaction (CTRL).
14. Incubate both reactions (RED and CTRL) for 1 h at -20 °C to precipitate RNA.
15. Centrifuge at ≥15,000 g for 30 min at 4 °C. Carefully decant the supernatant.
16. Wash the RNA with 1 ml of 70% ethanol. Vortex to detach the pellet for 5 min at room temperature.
17. Centrifuge at ≥15,000 g for 5 min at 4 °C.
18. Carefully remove the supernatant and air-dry the pellet.
19. Dissolve the pellet in 10 µl ddH_2O and quantify by Qubit RNA HS Assay (Thermo Fisher).

4 RNA abasic site production

The reduced m7G residues are subjected to nucleoside hydrolysis at low pH.

1. Prepare a sodium acetate solution (pH 3.9) by mixing 3.5 ml ddH_2O, 1.5 ml acetic acid and

300 µl 10 N sodium hydroxide (NaOH).

2. Add 1 µl 7-methyl-GTP (37 mM; Sigma-Aldrich) and 11 µl of acetate solution to the RNA dissolved in 10 µl of ddH_2O (ctrl or red).
3. Incubate the mix for 15 min at 37 °C.
4. Precipitate by adding 200 µl ddH_2O, 20 µl NaOAc (pH 4.5), 660 µl of ice-cold absolute ethanol and 1 µl glycogen.
5. Incubate for 1 h at -20 °C.
6. Centrifuge at ≥15,000 g for 30 min at 4 °C. Carefully decant the supernatant.
7. Wash the RNA with 1 ml of 70% ethanol. Vortex to detach the pellet.
8. Centrifuge at ≥15,000 g for 5 min at 4 °C.
9. Carefully remove the supernatant and air-dry the pellet.
10. Dissolve the pellet in 10 µl ddH_2O and quantify by Qubit RNA HS Assay.

5 ARP coupling and pulldown

A biotin-coupled aldehyde reactive probe covalently binds to the abasic RNA sites resulting from m7G reduction and hydrolysis. This allows pull-down with streptavidin-conjugated beads.

1. Prepare a 22.5 mM stock solution (4.5×) of Aldehyde Reactive Probe (ARP) by adding 1 ml ddH_2O to 10 mg of ARP powder (N-(aminooxyacetyl)-N'-(D-Biotinoyl) hydrazine, Trifluoroacetic Acid Salt; Thermo Fisher).
2. Prepare the 10× Reaction buffer containing 50 mM Tris-HCl (pH 6.8) and 10 mM EDTA.
3. Denature the RNA at 80 °C for 2 min.
4. Incubate RNA on ice for 5 min.
5. Prepare the ARP reaction according to Table 5.

Table 5: Preparation of ARP reaction mix

Volume	Reagent
4.5 µg	RNA (crtl or red)
8 µl	22.5 mM ARP (3 mM final)
6 µl	10× Reaction buffer
60 µl	Final vol. with ddH_2O

Table 6: Preparation of pulldown reaction

Volume	Reagent
2-3 µg	total RNA
200 µl	5× PD buffer
1 ml	Final vol. with ddH_2O

6. Incubate for 1 h at 30 °C.
7. Meanwhile prepare 1.33 M formaldehyde solution in water (dilution of 1:10 from 37% formaldehyde stock; Sigma-Aldrich).
8. To stop the reaction, add 2.25 µl 1.33 M formaldehyde solution, then add 40 µl ddH_2O, 10 µl 3 M sodium acetate and 300 µl absolute ethanol to the ARP samples.
9. Precipitate RNA at -20 °C for 1 h.
10. Pellet RNA by centrifugation at ≥15,000 g for 30 min at 4 °C.
11. Decant supernatant carefully and wash the pellet twice with 70% ethanol (step 7 of section 4).
12. Dissolve RNA in 50 µl ddH_2O.

13. Filter through an Illustra MicroSpin G-25 spin column (GE Healthcare) to remove the remaining unreacted ARP.
14. Prepare the 5× Pulldown (PD) buffer containing 50 mM Tris-HCl (pH 7.4), 750 mM sodium chloride (NaCl) and 0.5% NP-40 (v/v).
15. Wash 50 µl of Dynabeads MyOne C1 Streptavidin beads (Thermo Fisher) three times with 1× PD buffer.
16. Resuspend the beads in 1 ml 1× PD buffer + 0.5 mg/ml BSA (NEB).
17. Incubate for 1 h at 4 °C in rotation.
18. Prepare the PD reaction according to Table 6.
19. Add the blocked beads to the PD reaction.
20. Incubate at room temperature for 25 min in rotation.
21. Wash four times with ice-cold 1× PD buffer.
22. Add 700 µl QIAzol (Qiagen) and 140 µl chloroform to the beads.
23. Purify using the RNA Clean and Concentrator - 25 Kit (Zymo Research) according to the manufacturer's instructions.
24. Elute in 10-15 µl ddH_2O and quantify by Qubit RNA HS Assay.

6 7-Methylguanosine RNA Immunoprecipitation

Heat-denatured RNA is immunoprecipitated with a specific antibody against m7G. Both Input and IP samples are then purified using the same strategy in order to avoid any bias.

1. Denature decapped RNA for 2 min at 80 °C and place immediately on ice.
2. Save a 1-2.5 µg aliquot of RNA as INPUT control.
3. Prepare the 5× RIP buffer according to Table 7.
4. Prepare the RIP reaction according to Table 8.
5. Incubate the RIP reaction for 2 h at 4 °C in rotation.

Table 7: Preparation of 5× RIP reaction buffer

Volume	Reagent
0.5 ml	1 M Tris-HCl (pH 7.4)
1.5 ml	5 M NaCl
0.5 ml	10% NP-40 (v/v)
7.5 ml	ddH_2O

Table 8: Preparation of RIP reaction

Volume	Reagent
25 µg	total RNA
10 µg	anti-m7G antibody (RNO17M)
10 µl	RNAseOUT (Thermo Fisher)
200 µl	5× RIP buffer
1 ml	Final vol. with ddH_2O

6. Wash n×80 µl Dynabeads G (Thermo Fisher) twice with 1 ml 1× RIP buffer.
7. To block the Dynabeads, resuspend them in 1 ml 1× RIP buffer and add 50 µl 20 µg/µl BSA (NEB), then incubate for 2 h at 4 °C in rotation.
8. Wash the beads twice with 1× RIP buffer and resuspend them in n×100 µl 1× RIP buffer.
9. Add 100 µl beads to each RIP reaction.
10. Incubate for 2 h at 4 °C in rotation.

11. Wash three times with 1 ml ice-cold 1× RIP buffer, changing the 1.5 ml tube in the first wash.
12. Resuspend the beads in 100 µl 1× RIP buffer + 2 µl RNAseOUT (Thermo Fisher) + 6.7 mM 7-methyl-GTP (Sigma-Aldrich).
13. Incubate for 30 min at 37 °C to specifically elute m7G-harbouring RNAs, then collect the supernatant.
14. Bring the volume of the INPUT RNA control to 100 µl in 1× RIP buffer + 2 µl RNAseOUT + 6.7 mM 7-methyl-GTP.
15. Purify both INPUT and RIP samples with the RNA Clean and Concentrator - 25 Kit.
16. Elute in 10-15 µl ddH_2O and quantify by Qubit RNA HS Assay.

7 SmallRNA Sequencing Library Preparation

Sequencing libraries are prepared from resulting BoRed or m7G-RIP RNA with a commercial kit optimized to generate size-selected Small RNA libraries.

Prepare high-throughput libraries using NEBNext Small RNA Library Preparation kit for Illumina (NEB) starting from 1 µg of RNA for each sample and employing gel extraction as the final purification step, according to the manufacturer's protocol.

8 High Throughput Sequencing

Multiplexed libraries are sequenced by high-throughput sequencing-by-synthesis (Illumina).

Sequence the pooled libraries on Illumina platform running in 50-bp single-read mode. Plan the multiplexing strategy in order to get at least 15-20M raw reads per sample for BoRed-Seq and 5-10M reads for m7G-RIP.
