# Supplementary material for: METTL1 Promotes let-7 MicroRNA Processing via m7G Methylation
Source: Mol Cell. 2019 Jun 20;74(6):1278–1290.e9. doi: 10.1016/j.molcel.2019.03.040 (PMC6591002; doi:10.1016/j.molcel.2019.03.040)
Supplement: Document S2. Article plus Supplemental Information [file mmc9.pdf]

Molecular Cell

Article

METTL1 Promotes *let-7* MicroRNA Processing via m7G Methylation

Graphical Abstract

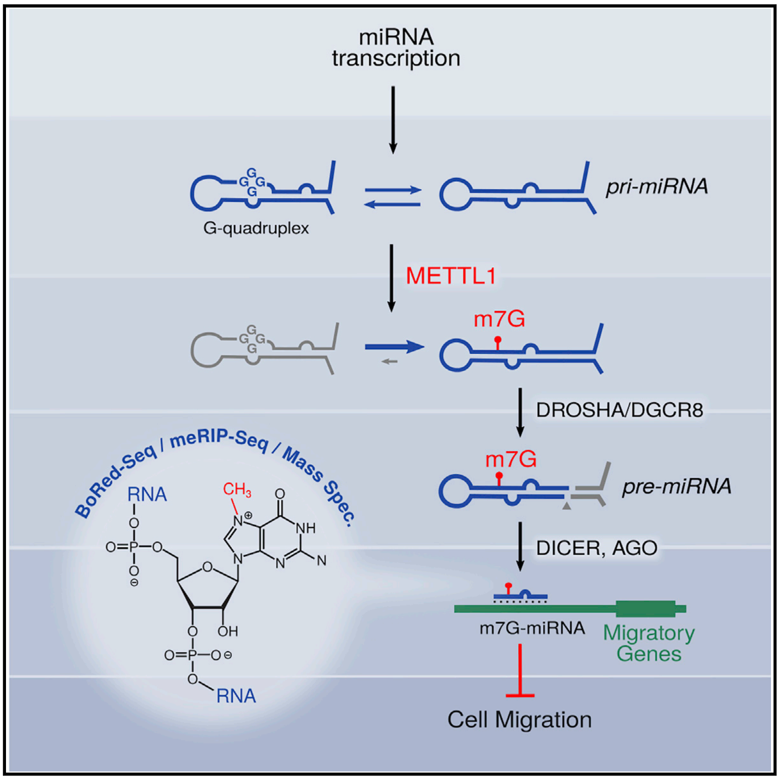

Highlights

- Internal m7G is identified in miRNAs by two independent sequencing techniques
- Methyltransferase METTL1 mediates m7G modification of specific miRNAs
- METTL1 promotes miRNA maturation and suppresses lung cancer cell migration
- m7G promotes processing by antagonizing G-quadruplex structures in miRNA precursors

Authors

Luca Pandolfini, Isaia Barbieri, Andrew J. Bannister, ..., Mara d'Onofrio, Shankar Balasubramanian, Tony Kouzarides

Correspondence

tony.kouzarides@gurdon.cam.ac.uk

In Brief

Pandolfini, Barbieri, et al. show that a subgroup of tumor suppressor microRNAs, including *let-7e*, contain 7-methylguanosine (m7G). Methyltransferase METTL1 is required for m7G modification of miRNAs, their efficient processing, and the inhibition of lung cancer cell migration. Structurally, m7G in miRNA precursors antagonizes RNA secondary structures that would otherwise inhibit their maturation.

Data Resource

GSE112182
GSE112180
GSE112181
GSE120454
GSE120455

Pandolfini et al., 2019, Molecular Cell *74*, 1278–1290
June 20, 2019 © 2019 The Author(s). Published by Elsevier Inc.
https://doi.org/10.1016/j.molcel.2019.03.040

Molecular Cell
Article

METTL1 Promotes *let-7* MicroRNA Processing via m7G Methylation

Luca Pandolfini,[1,9] Isaia Barbieri,[1,2,9] Andrew J. Bannister,[1] Alan Hendrick,[3] Byron Andrews,[3] Natalie Webster,[3] Pierre Murat,[4,7] Pia Mach,[1] Rossella Brandi,[5] Samuel C. Robson,[1,8] Valentina Migliori,[1] Andrej Alendar,[1] Mara d'Onofrio,[5,6] Shankar Balasubramanian,[4] and Tony Kouzarides[1,10,*]

[1]The Gurdon Institute and Department of Pathology, University of Cambridge, Tennis Court Road, Cambridge CB2 1QN, UK
[2]Division of Cellular and Molecular Pathology, Department of Pathology, University of Cambridge, Addenbroke's Hospital, Cambridge CB2 0QQ, UK
[3]Storm Therapeutics, Ltd., Moneta Building (B280), Babraham Research Campus, Cambridge CB22 3AT, UK
[4]Department of Chemistry, University of Cambridge, Lensfield Road, Cambridge CB2 1EW, UK
[5]Fondazione EBRI Rita Levi-Montalcini, Genomics Laboratory, Viale Regina Elena 295, 00161 Rome, Italy
[6]IFT-CNR, Via del Fosso del Cavaliere 100, 00133 Rome, Italy
[7]Present address: MRC Laboratory of Molecular Biology, Francis Crick Avenue, Cambridge CB2 0QH, UK
[8]Present address: School of Pharmacy & Biomedical Science, St. Michael's Building, University of Portsmouth, White Swan Road, Portsmouth PO1 2DT, UK
[9]These authors contributed equally
[10]Lead Contact
*Correspondence: tony.kouzarides@gurdon.cam.ac.uk
https://doi.org/10.1016/j.molcel.2019.03.040

SUMMARY

7-methylguanosine (m7G) is present at mRNA caps and at defined internal positions within tRNAs and rRNAs. However, its detection within low-abundance mRNAs and microRNAs (miRNAs) has been hampered by a lack of sensitive detection strategies. Here, we adapt a chemical reactivity assay to detect internal m7G in miRNAs. Using this technique (Borohydride Reduction sequencing [BoRed-seq]) alongside RNA immunoprecipitation, we identify m7G within a subset of miRNAs that inhibit cell migration. We show that the METTL1 methyltransferase mediates m7G methylation within miRNAs and that this enzyme regulates cell migration via its catalytic activity. Using refined mass spectrometry methods, we map m7G to a single guanosine within the *let-7e-5p* miRNA. We show that METTL1-mediated methylation augments *let-7* miRNA processing by disrupting an inhibitory secondary structure within the primary miRNA transcript (pri-miRNA). These results identify METTL1-dependent N7-methylation of guanosine as a new RNA modification pathway that regulates miRNA structure, biogenesis, and cell migration.

INTRODUCTION

Post-synthesis covalent modification of biological molecules is a key aspect of intracellular signaling, and it is critically important in many biological processes. RNA molecules, similar to proteins, are subject to a vast array of post-synthesis covalent modifications, which together constitute the epitranscriptome. To date, >100 RNA modifications have been identified, which are spread throughout every class of RNA and are evolutionarily conserved throughout all kingdoms of life (Carell et al., 2012; Machnicka et al., 2013).

RNA modifications have the potential to affect all RNA processes, including splicing, stability, and localization (Roundtree et al., 2017). Many RNA modifications have been identified by mass spectrometry (MS), and complex epitranscriptomes of tRNA and rRNA have been thoroughly studied. However, this represents a mere snapshot of a much bigger picture, with the clear majority of modifications remaining uncharacterized. This is predominantly due to a lack of sensitive methodologies with which to detect the modifications at a high resolution. Even now, MS methodologies are largely unable to generate transcriptome-wide modification profiles. However, a few very recent analyses have used anti-modification antibodies (e.g., against N1-methyladenosine [Dominissini et al., 2016], N6-methyladenosine [Dominissini et al., 2012], and 5-hydroxymethylcytosine [Delatte et al., 2016]) or chemical reactivity of the modification (for pseudouridine [Carlile et al., 2014; Schwartz et al., 2014], m_5C [Schaefer, 2015], and 2′-O-methylation [Dai et al., 2017]). Their results clearly suggest that many of the modifications identified on rRNA and tRNA are also present on other RNA classes. Therefore, the development of epitranscriptomic methodologies (e.g., new antibody and chemical methods coupled to next-generation sequencing [NGS]) represents a bottleneck in deciphering the function of new RNA modifications.

Certain nucleotides, such as 7-methylguanosine (m7G), display specific modification-dependent chemistries that can be exploited to study their prevalence and transcript location. m7G is present in eukaryotic mRNA 5′ caps and at defined internal positions within tRNAs and rRNAs across all domains of life. The best-characterized enzyme mediating internal m7G methylation is the TRMT8 yeast enzyme homolog METTL1 (methyltransferase-like 1), which, together with its co-factor WDR4

 © 2019 The Author(s). Published by Elsevier Inc.
This is an open access article under the CC BY license (http://creativecommons.org/licenses/by/4.0/).

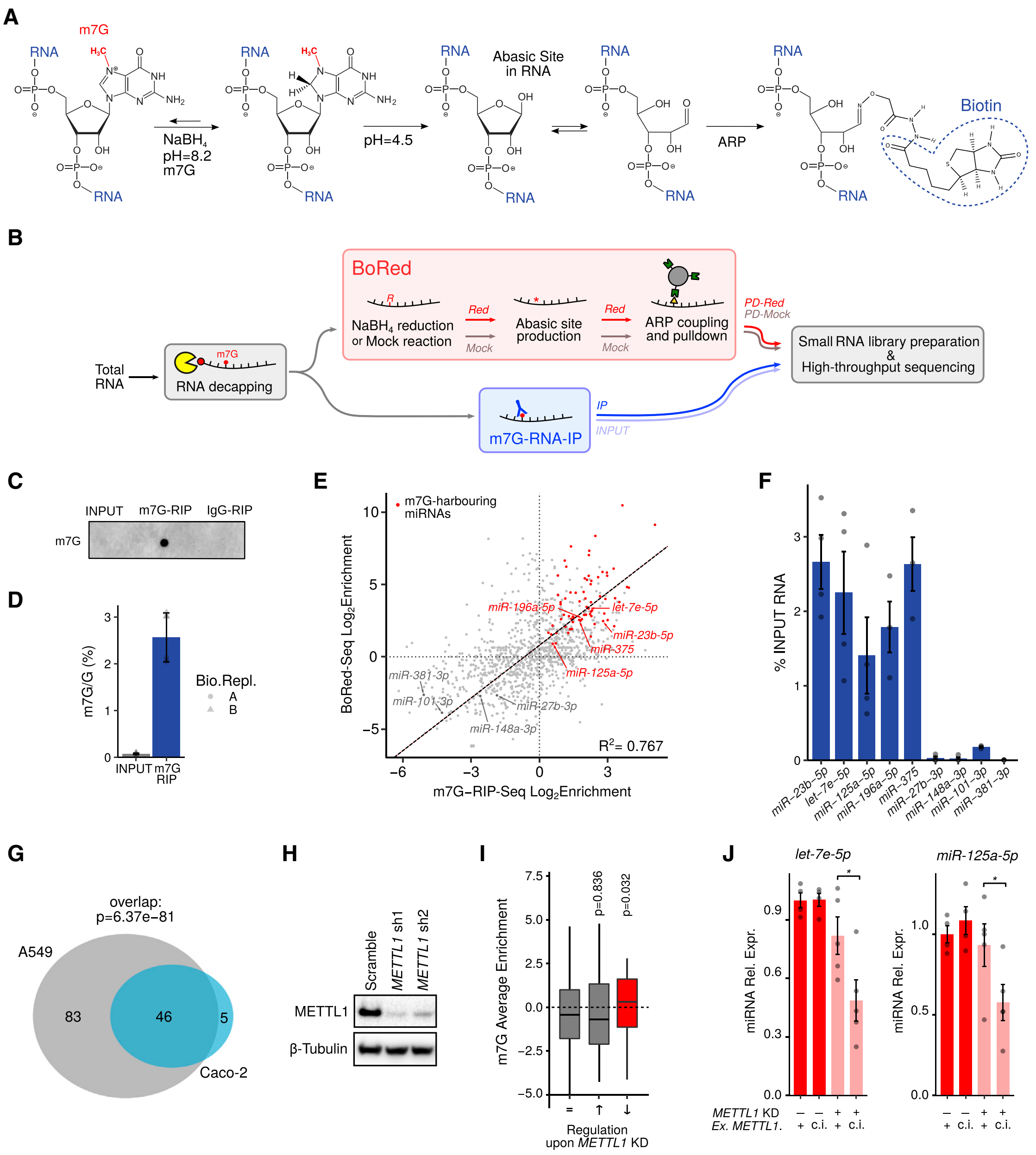

Figure 1. Detection of m7G in Specific miRNAs in A549 Cells

(A) Schematic of a novel chemical method to detect internal m7G RNA modification.

(B) Schematic representation of the procedure used to identify the m7G modified miRNAs in A549 cells.

(C) Immuno-dot blot of total decapped INPUT RNA (10%) or RNA immunoprecipitated with anti-m7G antibody or control immunoglobulin G (IgG).

(D) Immunoprecipitation with anti-m7G antibody enriches for m7G-containaing RNAs as determined by mass spectrometry (MS; see also Figure S1). The average of two biological replicates ± SDs is shown.

(E) Scatterplot showing a high degree of consistency between the BoRed-seq approach and RIP-seq in detecting miRNAs harboring m7G (upper right quadrant). Goodness of fit is calculated as R^2 Pearson correlation coefficient.

(F) RNA immunoprecipitation with the anti-m7G antibody coupled to qRT-PCR was used to validate five m7G-containing miRNAs and four negative miRNAs, which are identified in (E). The average of four biological replicates ± SDs is shown. The distributions of mean enrichments in m7G$^+$ and m7G$^-$ miRNAs are significantly different, as evaluated by the two-tailed Wilcoxon text (*$p < 0.05$).

(G) Venn diagram showing the overlap between miRNAs significantly enriched in m7G-RIP of A549 and Caco-2 cells, respectively (see also Figure S2). The p value is obtained by Fisher's exact test.

(H) Western blot showing METTL1 protein levels in A549 cells infected with *METTL1*-specific (sh1, sh2) or control (Scramble) tetracycline (TET)-inducible shRNAs 5 days after doxycycline treatment. A representative experiment of four independent biological replicates is shown.

(legend continued on next page)

(WD repeat domain 4), catalyzes m7G at G46 of specific tRNAs, such as $tRNA^{Phe}$ (Alexandrov et al., 2002).

In contrast to deoxy-m7G, m7G in RNA is highly stable in neutral aqueous solution (Kriek and Emmelot, 1964). The methylation significantly alters the charge density of RNA, potentially serving as a molecular handle, but it does not impair Watson-Crick G:C base complementarity. It does, however, interfere with non-canonical base pairing (i.e., Hoogsteen pairs), possibly affecting the secondary structure of RNA. Although relatively abundant, m7G has proved very difficult to study so far. Being neutral to Watson-Crick base pairing, it does not interfere with reverse transcription, rendering it invisible to detection by standard sequencing-based technologies.

microRNAs (miRNAs) are short single-stranded RNA molecules (18–24 nucleotides [nt]) that target the RNA interference silencing complex (RISC) to specific mRNAs. Their specificity is mediated by partial base pairing to sequences predominantly found in the 3′ UTR of mRNAs (Bartel, 2009). This interaction results in the decreased translation of the proteins they encode and/or in the degradation of the mRNAs themselves (Fabian et al., 2010; Ghildiyal and Zamore, 2009). To date, >1,000 human miRNAs have been identified, and they are key regulators of numerous physiological and pathological processes.

miRNA biosynthesis is complex and involves a multistep pathway that can be regulated at many levels (Bartel, 2018), including post-transcriptional modification of miRNA precursors (Alarcón et al., 2015; Xhemalce et al., 2012). miRNAs are synthesized from larger transcripts by RNA polymerase II or III. These primary miRNA transcripts (pri-miRNAs) are then cleaved by DROSHA to release hairpin-shaped RNAs called pre-miRNAs (Lee et al., 2003), and further cleaved by DICER to generate a miRNA duplex (Chendrimada et al., 2005). Certain miRNAs can form alternative secondary structures, such as G-quadruplexes, that can interfere with their processing (Mirihana Arachchilage et al., 2015; Pandey et al., 2015). However, little is known about the biological relevance of these structures in a physiological context.

Here, we develop two different but complementary high-throughput sequencing strategies to identify miRNAs harboring internal m7G modification. We show that METTL1 methylates a specific subset of tumor suppressor miRNAs, including *let-7*, to promote their processing from primary transcript to precursor miRNA. Depletion of METTL1 causes gene expression and phenotypic changes in a miRNA-dependent manner. We show that m7G-modified miRNAs have a propensity to form G-quadruplexes. We identify guanosine 11 as the m7G methylated residue within *let-7e-5p*, and we show that methylation at this position affects G-quadruplex formation, thereby promoting processing of the precursor miRNA.

RESULTS

To detect N7-methylguanosine within low-abundant RNAs, we adapted an existing strategy (Zueva et al., 1985) to allow the profiling of internal m7G in eukaryotic RNAs. In this reaction, m7G residues are prone to nucleoside hydrolysis when reduced by treatment with $NaBH_4$. The resulting abasic sites can be revealed by aniline-induced cleavage of the RNA chain by β-elimination. This reaction is the basis of direct RNA sequencing (RNA-seq) by the Maxam and Gilbert method (Peattie, 1979) and has been used for mapping m7G residues in highly abundant rRNAs and tRNAs at single nucleotide resolution (Zueva et al., 1985). We optimized the reaction conditions for the reduction of mammalian total RNA in the absence of methylated carrier RNA, which would interfere with the NGS analysis. As proof of principle, reduced *18S* rRNA was cleaved by aniline treatment into two fragments, in agreement with the known position of m7G (Piekna-Przybylska et al., 2008; Figure S1A).

The above strategy is not suitable for very short RNAs such as miRNAs, because the resulting cleavage fragments would be too small to be unequivocally mapped to the human transcriptome. Therefore, we developed a new protocol, based on the above, to detect m7G within miRNAs, which we refer to as Borohydride Reduction (BoRed-seq) (Figure 1A). Total RNA from a human lung cancer cell line (A549 cells) was decapped, treated with $NaBH_4$, and exposed to low pH to generate abasic sites at positions harboring m7G. These sites were exposed to a biotin-coupled aldehyde reactive probe (*N*-(aminooxyacetyl)-*n*′-(D-biotinoyl) hydrazine; ARP) that covalently binds to abasic RNA sites (Tanaka et al., 2011). Modified RNAs were then pulled down using streptavidin beads, small RNA libraries were prepared, and RNAs were identified by high-throughput sequencing. Using this approach (Figure 1B), a number of mature miRNAs likely to contain m7G were identified (Table S1).

To confirm the validity of this technique and to provide an independent verification of m7G-methylated miRNAs, we performed an RNA immunoprecipitation sequencing (RIP-seq) experiment using an antibody that recognizes m7G in RNA (Figure 1C). This antibody immunoprecipitates m7G-containing RNAs, but not other methylated G-containing RNAs (as judged by MS; Figures 1D and S1B–S1D), and it specifically enriches m7G-containing *18S* rRNA and tRNAs (Figures S2A and S2B). RIP-seq with this antibody identified a second cohort of mature miRNAs containing m7G (Table S2).

We then compared the results from the BoRed-seq and RIP-seq approaches and found there was a significant overlap of m7G-modified miRNAs detected by each technique (Figures 1E, upper right quadrant, S2C, and S2D). We regard these miRNAs as high-confidence m7G-modified miRNAs (Table S3), five of which were validated by RIP-qPCR analysis (Figure 1F). m7G is found on miRNAs of any abundance, bearing no correlation with any particular expression level (Figure S2E).

We extended these analyses to an unrelated colorectal cancer cell line (Caco-2 cells), which expresses METTL1 at levels comparable to those observed in A549 cells. This identified significantly overlapping m7G-modified miRNAs (Figures 1G and

(I) Boxplot showing increased m7G signal (as an average enrichment in both BoRed-seq and m7G-RIP-seq; E) in miRNAs that are significantly downregulated (↓) upon inducible *METTL1* knockdown, but not in miRNAs that are unchanged (=) or upregulated (↑). Statistical significance was calculated by the Wilcoxon test.
(J) qRT-PCR showing the levels of *let-7e-5p* and *miR-125a-5p* in WT and *METTL1* knockdown A549 cells in the presence of either active (+) or catalytically inactive (c.i.) exogenous METTL1 (Ex. METTL1).

Table 1. miRNAs Harboring METTL1-Dependent m7G

	BoRed-Seq		m7G-RIP-Seq	
miRNA	Log2Enrich	FDR	Log2Enrich	FDR
hsa-let-7a-5p *(im)*	3.793	1.47E−35	0.923	7.05E−12
hsa-let-7b-3p	3.650	5.36E−20	0.923	1.26E−11
hsa-let-7b-5p *(im)*	3.848	5.38E−40	1.756	1.25E−27
hsa-let-7c-5p *(im)*	4.049	6.87E−39	1.128	4.61E−15
hsa-let-7e-5p *(im)*	3.357	5.77E−30	2.149	6.00E−32
hsa-miR-125a-5p *(im)*	0.921	3.76E−03	0.575	3.46E−03
hsa-miR-149-3p	3.227	2.37E−02	2.306	3.03E−14
hsa-miR-193a-5p	1.673	8.71E−03	0.743	9.27E−04
hsa-miR-23b-5p *(im)*	2.490	6.78E−06	2.789	3.32E−54
hsa-miR-320a *(im)*	1.924	4.26E−11	1.336	1.07E−14
hsa-miR-320b *(im)*	2.568	6.54E−16	1.740	7.94E−54
hsa-miR-320c *(im)*	3.465	1.81E−20	2.066	3.88E−50
hsa-miR-320d	2.968	1.84E−09	2.217	4.53E−59
hsa-miR-320e	2.794	1.15E−02	1.460	1.89E−09
hsa-miR-328-3p	1.669	8.85E−03	1.260	2.17E−15
hsa-miR-505-5p	2.830	3.12E−02	2.274	1.52E−13
hsa-miR-663a *(im)*	6.671	3.23E−43	1.106	7.60E−04
hsa-miR-760	3.142	5.18E−04	2.258	1.93E−31
hsa-miR-92b-3p	2.416	7.64E−14	1.459	1.72E−27
hsa-miR-92b-5p	4.302	1.06E−30	0.805	1.84E−07

Table shows m7G-modified miRNAs (from Figure 1E) whose expression is downregulated upon *METTL1* knockdown. miRNAs highlighted with superscript *(im)* have been linked to the inhibition of cellular migration (Zhang et al., 2011). FDR, false discovery rate.

S2F; Table S4), suggesting that m7G modification of miRNAs is a general and conserved phenomenon.

Deposition of m7G in tRNA is catalyzed, at least in part, by METTL1. We therefore asked whether any of our high-confidence m7G-containing miRNAs are affected by METTL1 depletion. Knockdown of *METTL1* in A549 cells (Figures 1H and S2G) followed by small RNA-seq revealed that significantly downregulated miRNAs are more enriched in m7G, compared to miRNAs that are upregulated or unchanged (Figure 1I; Table S5). Similar effects were also observed in Caco-2 cells (Figure S2H). The reduced levels of m7G-containing miRNAs are rescued by the expression of wild-type (WT) METTL1 but not by a catalytically inactive version (Figures S2I and S2J) of the enzyme (Figure 1J).

Interrogation of the m7G-containing miRNAs downregulated upon *METTL1* knockdown (Table 1) shows that 50% (10/20) of them have been previously functionally linked to the inhibition of cell migration (Zhang et al., 2011). This raised the possibility that METTL1 may control cell migration via regulation of a subset of miRNAs, including the *let-7* family (Lee and Dutta, 2007). To explore this possibility, we first tested whether METTL1 affects the migration of A549 cells. Knockdown of *METTL1* significantly increases their migratory capacity (Figures 2A and 2B) without affecting cellular proliferation (Figure 2C) or overall mRNA translation levels (Figure S2K). Notably, the increased migration is rescued by the expression of WT METTL1, but not by a catalytically inactive version of the enzyme (Figure S3A). These results suggest that METTL1 specifically influences cell migration via m7G methylation of miRNAs.

To further explore this possibility, we performed a global gene expression analysis to identify transcripts affected by the depletion of METTL1 (Figure 2D; Table S6). This revealed 254 upregulated and 60 downregulated transcripts. Gene Ontology analysis indicated the upregulation of pathways involved in cellular migration (Figures 2E, and S4B–S4E; Table S7), in agreement with our phenotypic characterization of *METTL1* knockdown cells (Figures 2A and 2B).

We then asked whether METTL1-regulated transcripts are also targets of the m7G-modified miRNAs. *In silico*-predicted mRNA targets for these miRNAs are differentially expressed upon *METTL1* knockdown, whereas mRNA targets of control miRNAs are not (Figure 2F). We confirmed these findings using an unbiased approach that identified *let-7(5p)* seed sequence as the most significantly enriched in upregulated mRNAs (Figure S3F), a number of which are individually shown in Figure S3G. The presence of the *let-7* target sequence within the 3′UTR of mRNAs represents the strongest predictive factor for their upregulation upon *METTL1* knockdown (Figure S3H).

The above results indicate that many of the genes involved in cell migration and upregulated upon METTL1 depletion are targets of METTL1-dependent miRNAs. This suggests that METTL1 regulates gene expression via the control of miRNA function. To investigate this possibility, we focused on *HMGA2,* one of the most upregulated transcripts following METTL1 depletion, and whose 3′ UTR is significantly enriched for evolutionarily conserved target sites of several m7G-containing miRNAs, including *let-7(5p)*, *miR-125(5p)*, and *miR-92(3p)* (Figure 3A; odds ratio [OR] = 5.46, p = 0.001). First, we confirmed that *METTL1* knockdown increases *HMGA2* mRNA expression and protein levels in both A549 cells (Figures 3B and 3C) and Caco-2 cells (Figures S4A and S4B). Second, we confirmed that *HMGA2* mRNA is not m7G modified itself by performing BoRed-qPCR and RIP-qPCR (Figure S4C).

To demonstrate that the effect of METTL1 was mediated, at least in part, by a miRNA pathway, we generated a stable A549 cell line containing a reporter construct consisting of the 3′ UTR of *Hmga2* linked to the coding sequence of luciferase. Knock down of *METTL1* in this reporter line increases luciferase activity, confirming that the 3′ UTR of *HMGA2* confers responsiveness to METTL1 (Figures 3D and 3E). To confirm that METTL1 regulation of the 3′ UTR of *HMGA2* is mediated through the action of miRNAs, we concentrated on *let-7*, which has seven binding sites within this 3′ UTR. Deletion of the *let-7* seed sequences from the 3′ UTR leads to increased luciferase activity and uncouples it from METTL1 regulation (Figures 3E and S4D).

Figure 3F shows that the introduction of mature *let-7e-5p* miRNA into A549 cells reduced HMGA2 protein expression as expected (Mayr et al., 2007). Transfection of mature *let-7e* miRNA reverts the upregulation of HMGA2 protein caused by METTL1 depletion (Figure 3F). Expression of a short hairpin RNA (shRNA)-resistant version of WT METTL1 also reverts HMGA2 upregulation due to METTL1 depletion, and this requires the catalytic activity of the methyltransferase (Figure 3G). These data confirm that METTL1 methyltransferase activity regulates the expression of *HMGA2* in a *let-7*-dependent manner.

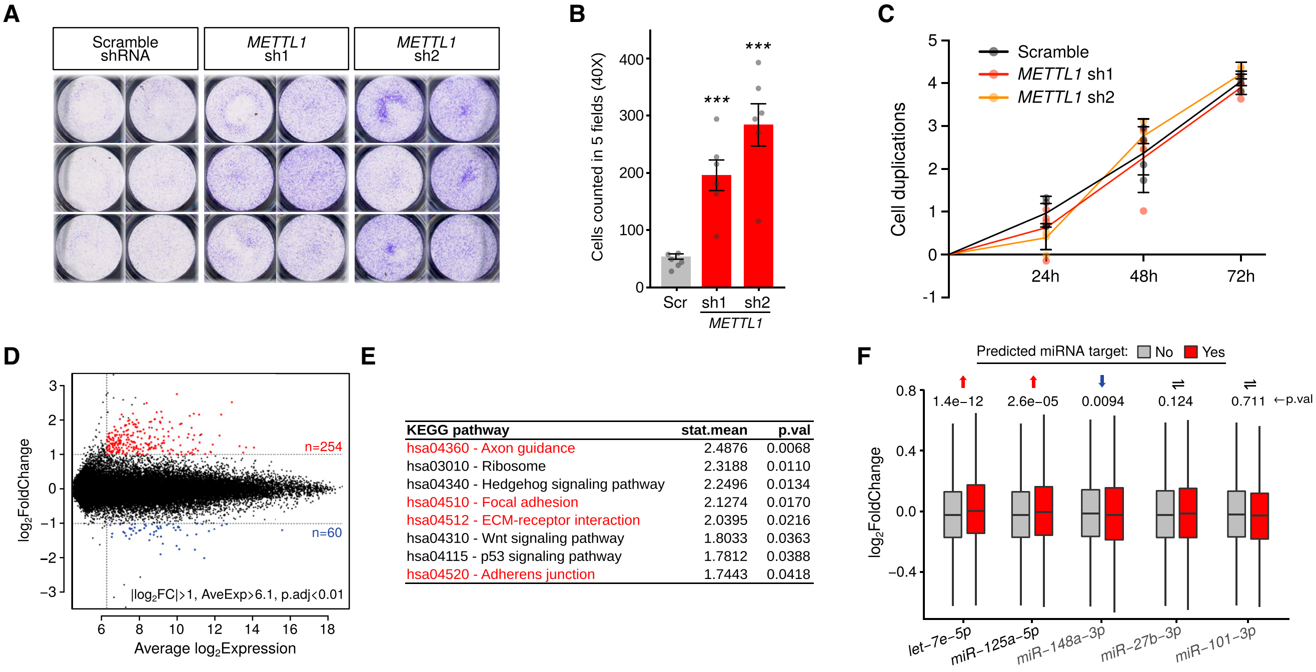

KEGG pathway	stat.mean	p.val
hsa04360 - Axon guidance	2.4876	0.0068
hsa03010 - Ribosome	2.3188	0.0110
hsa04340 - Hedgehog signaling pathway	2.2496	0.0134
hsa04510 - Focal adhesion	2.1274	0.0170
hsa04512 - ECM-receptor interaction	2.0395	0.0216
hsa04310 - Wnt signaling pathway	1.8033	0.0363
hsa04115 - p53 signaling pathway	1.7812	0.0388
hsa04520 - Adherens junction	1.7443	0.0418

Figure 2. METTL1 Inhibits Cellular Migration of A549 Cells

(A) A migration assay was performed for 7 h using cells infected with *METTL1*-specific (sh1, sh2) or control (Scramble) TET-inducible shRNAs 5 days after doxycycline treatment.

(B) Results from (A) were quantitated and plotted, as indicated. The plot shows the average of six biological replicates ± SDs (***$p < 0.001$, two-tailed t test).

(C) A proliferation assay was initiated 4 days after doxycycline treatment of cells infected with *METTL1*-specific (sh1, sh2) or control (Scramble) TET-inducible shRNAs. The average of four biological replicates ± SDs is shown.

(D) Global gene expression analysis of cells infected with *METTL1*-specific (sh1) or control (Scramble) TET-inducible shRNAs 5 days after doxycycline treatment. $\log_2$ fold change was plotted against average $\log_2$ expression. Significantly upregulated (red) and significantly downregulated (blue) transcripts are indicated. See also Figure S3.

(E) Gene Ontology analysis of gene expression changes following *METTL1* knockdown identifying upregulated Kyoto Encyclopedia of Genes and Genomes (KEGG) pathways involved in cellular migration (red).

(F) $\log_2$ fold change in the expression of predicted targets of the indicated miRNAs upon *METTL1* knockdown. Each pair of boxplots compares the fold change of mRNAs that are targets (red) or not (gray) of a single specific miRNA. Statistical significance was calculated by the Wilcoxon test.

We next explored the functional role of *let-7* methylation by METTL1. We used UV cross-linking and immunoprecipitation (CLIP) to confirm in our system previous findings (Bao et al., 2018) that METTL1 binds directly to miRNA precursors, including *pri-let-7e* and *pri-miR-125a* hairpins (Figures 4A and S4E). To date, there are two known methylations of miRNA: m6A catalyzed by METTL3 (Alarcón et al., 2015) and 5′-phosphate methylation catalyzed by BCDIN3D (Xhemalce et al., 2012). In both cases, methylation regulates miRNA processing. To test whether METTL1 may also be involved in such a pathway, we asked whether it regulates the processing of m7G-containing miRNAs. Depletion of METTL1 significantly reduced the levels of the premature and mature forms of *let-7e-5p* and *miR-125a-5p*, whereas their primary transcripts were unaffected (Figures 4B, 4C, and S4F). This is unlikely to be due to a general defect in processing since METTL1 depletion does not affect the levels of various miRNA processing factors (Figure S4G).

The above findings indicate that METTL1 activity is required for pri- to pre-processing of miRNAs, implying that pri-miRNAs are directly m7G modified by METTL1 and that the modification is subsequently retained through to the mature miRNA (Figure S4H). m7G RIP experiments show that pri-miRNAs are enriched in m7G, which decreases upon *METTL1* knockdown (Figures 4D, S4I, and S4J).

To further demonstrate the METTL1-dependent effect on miRNA processing, we used an *in vitro* assay (Lee et al., 2002) to test the efficiency of cellular extracts to process a radioactive *pri-let-7e* transcript into precursor and mature miRNAs. Cellular extracts devoid of METTL1 process precursor transcripts less efficiently than control extracts (Figure S5A). Thus, METTL1 is required for the efficient processing of target miRNAs such as *let-7*.

To confirm that *pri-let-7e* is directly methylated by METTL1, we performed an *in vitro* methyltransferase assay using a preassembled recombinant METTL1/WDR4 complex (Figure S5B) and *pri-let-7e* hairpin oligonucleotides, $tRNA^{Phe}$, and an unrelated negative control miRNA (*cel-miR-67* hairpin) as substrates. Using MS, we detected m7G in *let-7e* RNA and $tRNA^{Phe}$, but not in the control miRNA (Figures 4E and S5C).

To directly assess whether m7G affects miRNA processing, we prepared radiolabeled m7G containing *pri-let-7e* RNA, as shown in Figure 4F. Briefly, radioactive *pri-let-7e* hairpin

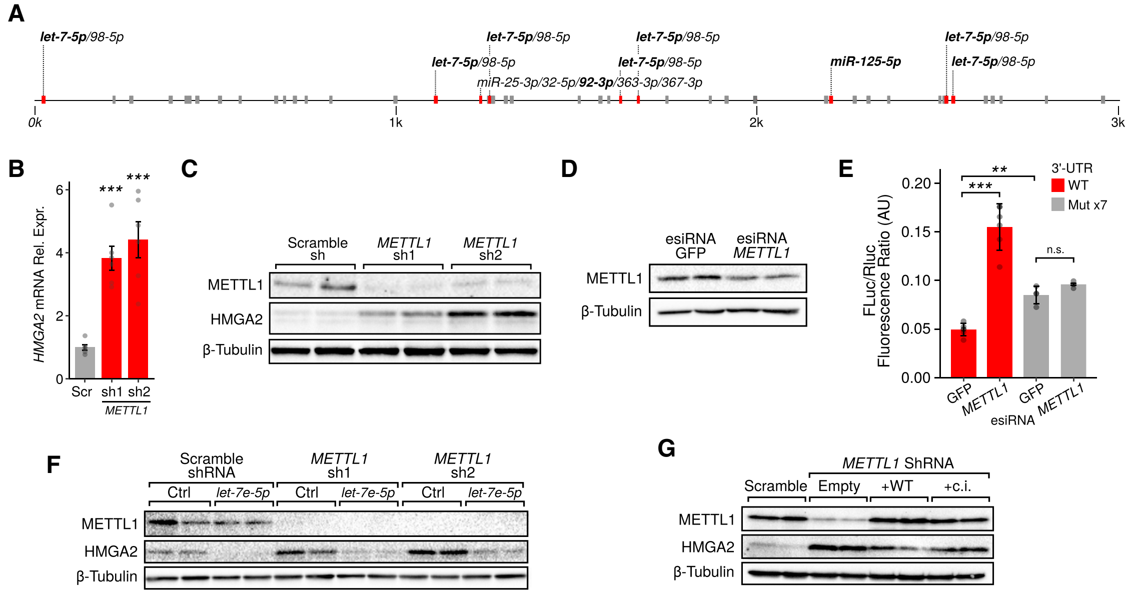

Figure 3. METTL1 Catalytic Activity Regulates *HMGA2* Expression in a *let-7*-Dependent Manner

(A) Schematic of *HMGA2* 3′ UTR showing the enrichment of evolutionarily conserved target sites of several m7G-containing miRNAs (OR = 5.46, p = 0.001).

(B) *HMGA2* expression was measured by qRT-PCR in A549 cells infected with *METTL1*-specific (sh1, sh2) or control (Scr) TET-inducible shRNAs 5 days after doxycycline treatment. The average of six biological replicates ± SDs is shown (***$p < 0.001$, two-tailed t test).

(C) Western blot showing METTL1, HMGA2, and β-tubulin protein levels in A549 cells infected with *METTL1*-specific (sh1, sh2) or control (Scramble) TET-inducible shRNAs 5 days after doxycycline treatment. Two representative biological replicates of a total of four are shown.

(D) Western blot showing METTL1 downregulation upon transfection with *METTL1*-specific siRNAs in A549 cells stably expressing a luciferase cDNA with *Hmga2* 3′ UTR. Two independent transfections of a total of four replicates are shown.

(E) Luciferase fluorescence levels upon *METTL1* downregulation in A549 cells stably expressing a luciferase cDNA with *Hmga2* 3′ UTR as a reporter. Red and gray bars indicate luciferase levels in the presence of either WT *Hmga2* 3′ UTR or of a variant in which all 7 *let-7* seed sequences have been mutated, respectively. The plot shows the average of four independent transfections ± SDs (***$p < 0.001$, two-tailed t test).

(F) Western blot showing the rescue of HMGA2 upregulation upon transfection with *let-7e-5p* mature miRNA in *METTL1* knockdown A549 cells. Two independent transfection replicates of a total of four are shown.

(G) Western blot showing the rescue of HMGA2 upregulation upon the overexpression of WT, but not catalytically inactive METTL1, in A549 *METTL1* knockdown cells. Two representative biological replicates of a total of five independent infections are shown.

See also Figure S4.

RNA was methylated *in vitro* by incubation with the METTL1/WDR4 complex, and m7G containing RNA was enriched via RIP. The resulting methylated and non-methylated (control) pri-miRNAs were then subjected to a DROSHA processing assay. The results indicate that m7G methylated *pri-let-7e* RNA was more efficiently processed by DROSHA *in vitro* (Figure 4G).

To probe the mechanism by which m7G methylation of *let-7e* affects its processing *in vivo*, we sought to identify the position of the methylation site within the *let-7e* miRNA. We set up spectral sequencing of RNA methylation and applied it to mature miRNAs purified from A549 cells. This targeted MS method allows the mapping of modification sites within a specific sequence at single base resolution. This approach highlighted one methylated guanosine at position 11 (G11) of *let-7e-5p* (Figures 5A, 5B, and S5D), which is also required for efficient *in vitro* methylation of *let-7e* (Figure S5E).

G11 is part of a short 16-nt-long G-rich sequence of the form $G_{2+}N_4G_{2+}N_4G_{2+}N_4G_{2+}$, where N is any base. This type of motif is known to fold into the alternative Hoogsteen base-paired G-quadruplex structure (Kwok et al., 2016a). It is noteworthy that the formation of a G-quadruplex structure has been documented in three miRNA precursors, namely *miR-92b* (Mirihana Arachchilage et al., 2015), *miR-149* (Kwok et al., 2016b), and *let-7e* (Pandey et al., 2015). According to our previous results, all of these miRNAs are both m7G modified and METTL1 dependent (Table 1), suggesting that G-quadruplex formation may be involved in the regulation of m7G-modified miRNAs.

To explore the connection between G-quadruplexes and m7G, we analyzed the base composition of m7G harboring miRNAs, assessing the potential enrichment of G-quadruplex motifs. We found that m7G-modified miRNAs are characterized by a bias in nucleotide content toward increased G-richness (Figure S6A) and G-skewness (Figure 5C). These observations suggest that m7G harboring miRNAs display sequences with the propensity to form G-quadruplexes. Moreover, using a G-quadruplex-predicting algorithm, we found that miRNAs containing at least one predicted G-quadruplex are significantly enriched in m7G (Figures 5D and S6B). The G-quadruplexes are predicted to fold at a very similar relative position within different m7G-containing miRNA hairpins (Figure 5E), which overlaps the 5′ site of pri-miRNA (DROSHA) cleavage. Overall, these analyses suggest

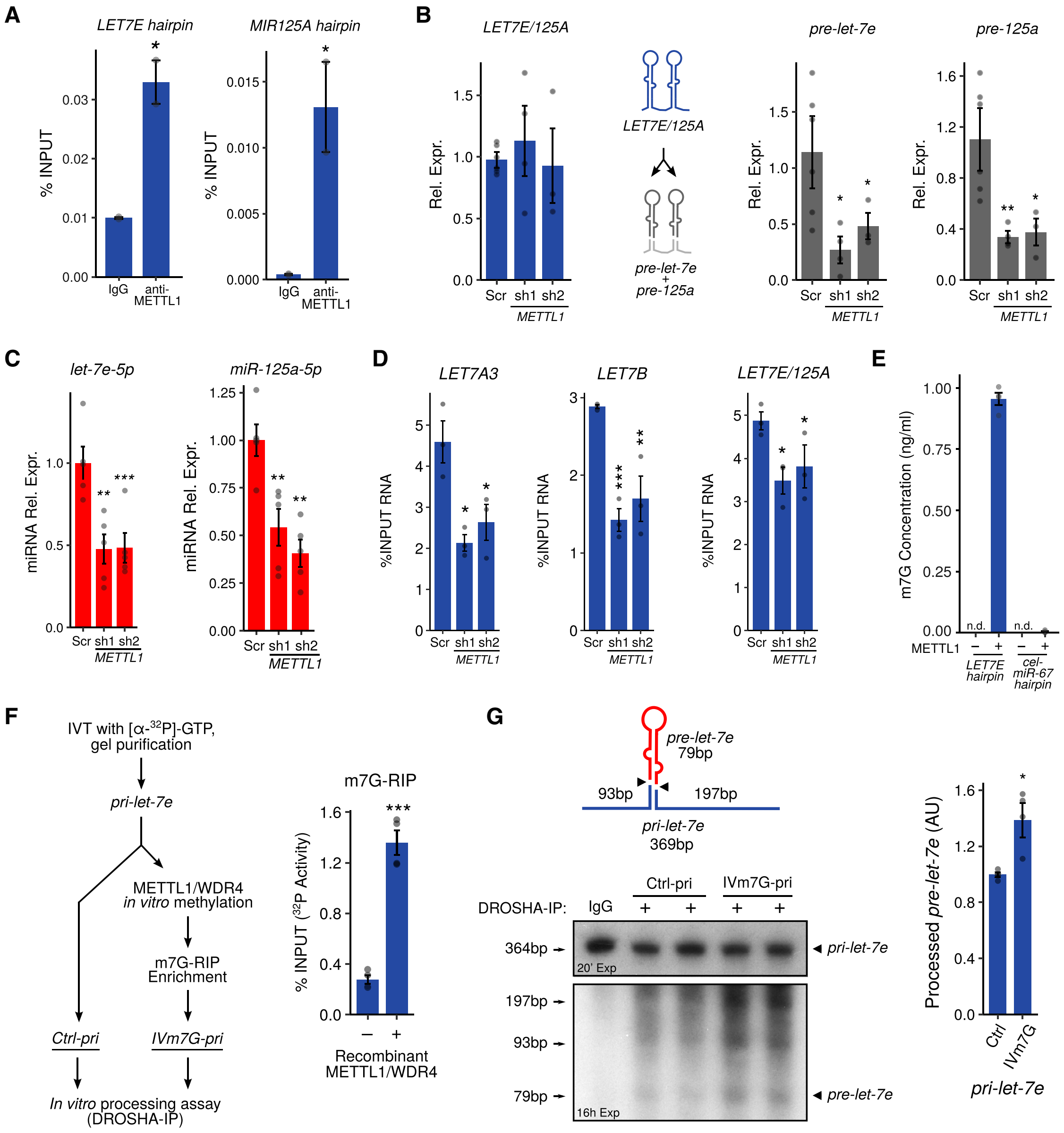

Figure 4. METTL1 Directly Modifies *let-7e* pri-miRNA and Regulates Its Processing

(A) CLIP-qPCR using a METTL1-specific antibody or a non-specific IgG. The levels of immunoprecipitated *pri-let-7e* and *pri-mir-125a* hairpins are shown. The average of two independent immunoprecipitation reactions ± SEMs is shown (*$p < 0.05$, two-tailed t test). *miR-148a* is shown in Figure S4E as a negative control.

(B) qRT-PCR showing the levels of either *LET7E/125A* primary transcript (blue) or *let-7e* and *miR-125a* precursors (gray) upon *METTL1* knockdown in A549 cells. The average of five to six independent biological replicates ± SDs is shown (*$p < 0.05$, **$p < 0.01$, two-tailed t test).

(C) qRT-PCR quantification of *let-7e-5p* and *miR-125a-5p* upon *METTL1* knockdown. The average of five independent biological replicates ± SDs is shown (**$p < 0.01$, ***$p < 0.001$, two-tailed t test). *miR-148a-3p* is shown in Figure S4F as a negative control.

(D) m7G RNA immunoprecipitation and qRT-PCR of *LET7A3*, *LET7B*, and *LET7E/125A* primary transcripts in A549 cells upon *METTL1* knockdown. The average of three independent biological replicates ± SEMs is shown (*$p < 0.05$, **$p < 0.01$, ***$p < 0.001$, two-tailed t test).

(E) *In vitro* methylation reaction using recombinant METTL1/WDR4 pre-assembled complex on *let-7e* or *cel-miR-67* primary hairpin (negative control). MS analysis shows specific m7G methylation of the *let-7e* hairpin. The average of three independent experiments ± SDs is shown.

(F) Experimental strategy to obtain radiolabeled, m7G-modified *pri-let-7e* (*IVm7G-pri*). The histogram shows the fraction of RNA recovered by m7G-RIP after *in vitro* methylation with METTL1/WDR4, as evaluated by scintillation counting (***$p < 0.001$, two-tailed t test).

(G) *In vitro* processing assay of *pri-let-7e*: control (Ctrl) or *in vitro* methylated pri-miRNAs were incubated in the presence of immunoprecipitated DROSHA. Autoradiography reveals that *IVm7G-pri* undergoes more efficient processing, yielding the expected cleavage pattern shown in the illustration. The histogram shows the relative quantification of the resulting *pre-let-7e* from four samples obtained in two independent experiments (*$p < 0.05$, two-tailed t test). Autoradiography images are composites of different molecular weight regions and exposure times. Full, unprocessed images are deposited on Mendeley Data.
See also Figure S5.

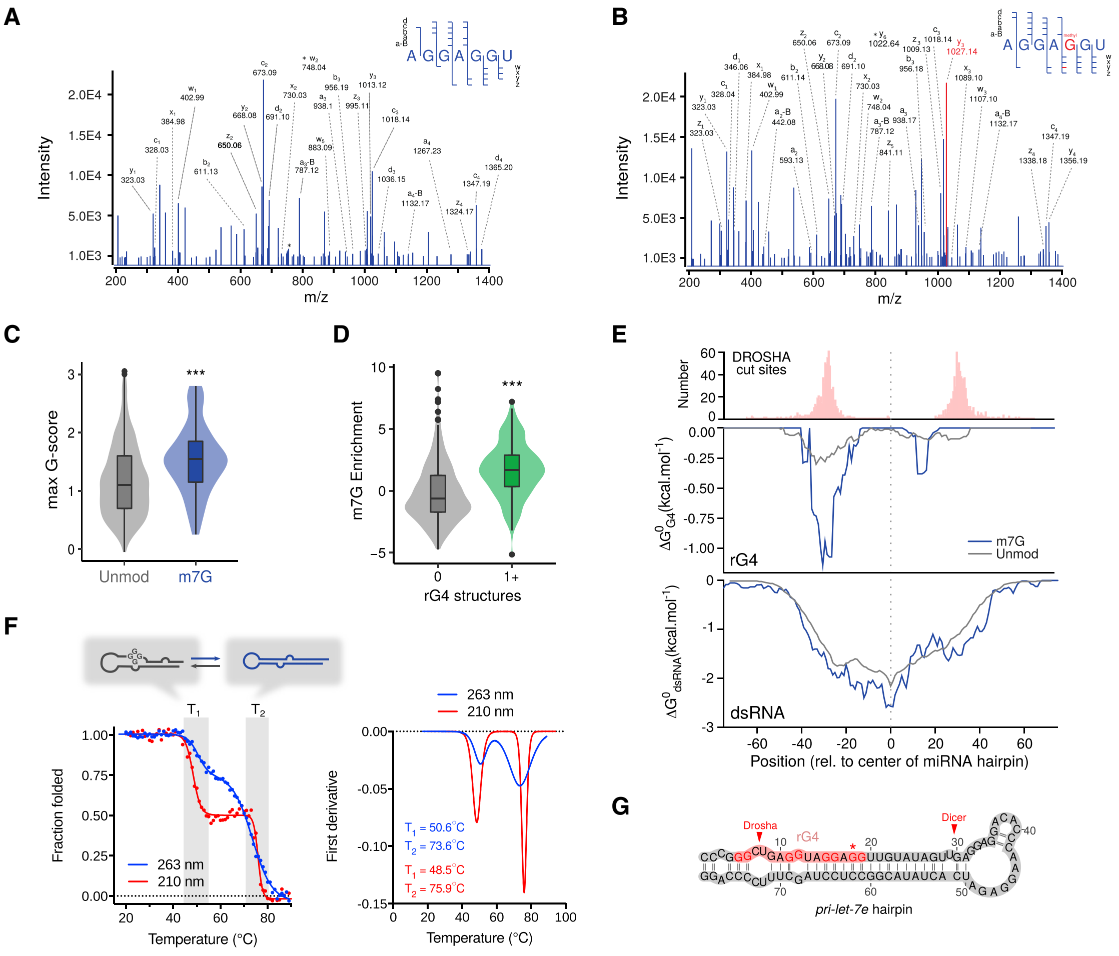

Figure 5. G-Quadruplexes Mark m7G-Containing miRNAs

(A and B) Spectral sequencing of *in vivo let-7e-5p* showing unmodified (A) and methylated 5′-AGGAGGU-3′ (B) fragments, obtained following RNase A digestion of a miRNA fraction isolated from A549 cells (see also Figure S5).

(C) Boxplot showing the maximum G-score, a quantitative estimation of G-richness and G-skewness (see Method Details for definition), in primary hairpins of either unmodified or m7G containing miRNAs (***p < 0.001, Wilcoxon test).

(D) Boxplot showing the enrichment of miRNAs, grouped according to the propensity of their primary hairpins to form G-quadruplexes (***p < 0.001, Wilcoxon test).

(E) Metagene plot showing pri-miRNA cleavage site distribution (top) and the predicted stability of G-quadruplexes (center), and double strand (bottom) across primary hairpins of unmodified (gray) or m7G-modified miRNAs (blue).

(F) Denaturation experiments of *let-7e* primary hairpin in the presence of 100 mM KCl followed by circular dichroism at 263 or 210 nm show two transitions demonstrating that *let-7e* exists as a mixture of two distinct structures in equilibrium in solution (top). The first structure melts at 48.5°C–50.6°C, while the second one is more stable (75.9°C–73.6°C).

(G) Scheme showing the predicted G-quadruplex (rG4, pink) within the *pri-let-7e* hairpin. In red are shown the guanosines predicted to be involved in the formation of the quadruplex motif. Arrows mark the cleavage sites of *let-7e-5p* processing. The asterisk indicates the position of m7G.

See also Figure S6.

that G-quadruplexes contribute to the METTL1-mediated regulation of miRNA activity.

To support a role for G-quadruplex formation in the processing of *pri-let-7e*, we carried out a biophysical analysis of the short 16-nt-long G-rich sequence, referred to as *rG4-let-7e*. Using circular dichroism (CD), we found that the CD spectrum of *rG4-let-7e* is cation dependent and is characterized by a maximum ellipticity at 263 nm and a minimum ellipticity at 240 nm (Figure S6C). This observation is consistent with G-quadruplex formation (Kypr et al., 2009). Denaturation experiments revealed a potassium-dependent transition at 48.1°C (Figure S6C). We then assessed the ability of this sequence to fold into a G-quadruplex in the context of *pri-let-7e*. The CD spectrum of *pri-let-7e* also displayed a maximum at 263 nm and a local minimum at 240 nm, but with an additional minimum at 210 nm indicative of a more complex structure (Figure S6D). Denaturation experiments of *pri-let-7e*, followed by CD spectroscopy at 263 or 210 nm in the presence of KCl, revealed two transitions indicating the presence of two structures in equilibrium (Figure 5F). While the second transition is centered

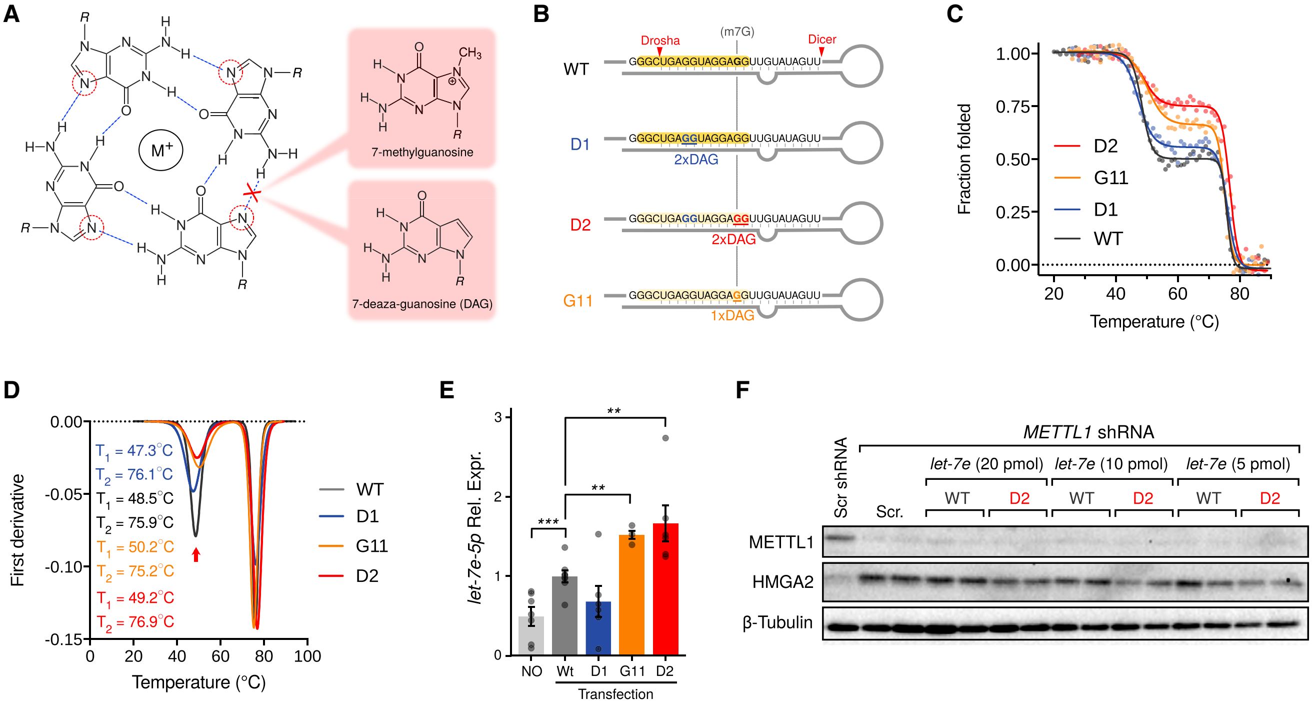

Figure 6. m7G Position Is Essential for *let-7e* Quadruplex:Stem-Loop Equilibrium and Promotes miRNA Processing

(A) Schematic representation of a guanine tetrad, highlighting Hoogsteen base pairing involving the N7 of guanosine that stabilizes the G-quadruplex structure, together with a stabilizing monovalent cation (M^+, usually potassium). Both 7-methylguanosine and 7-deaza-guanosine are able to destabilize the hydrogen bond involving N7.

(B) Illustration depicting the pri-miRNA hairpins used in the following experiments.

(C) Thermal denaturation studies of RNA oligonucleotides as described in (B). While GG-to-DAG-DAG mutation at the D1 position does not significantly affect the contribution of G4 in the G4:stem-loop equilibrium, GG-to-DAG-DAG mutation at the D1 position and a single G11-to-DAG mutation affect the contribution of rG4 in the structural equilibrium by shifting it toward the hairpin form.

(D) First derivative plot of the denaturation experiment in (C) helps visualize the decrease in rG4 contribution to the equilibrium (red arrow).

(E) qRT-PCR showing the levels of *let-7e-5p* 72 h after transfection with either WT, D1, D2, or G11 oligonucleotides. The average of six independent transfections ± SDs is shown (**$p < 0.01$, ***$p < 0.001$, two-tailed t test).

(F) Western blot showing the rescue of HMGA2 upregulation upon transfection of D2, but not WT *let-7e* primary hairpin in A549 *METTL1* knockdown cells. Two representative biological replicates of a total of three independent experiments are shown.

around ~75°C and correspond to the expected hairpin structure, a first transition was observed at ~48°C, similar to the melting transition of *rG4-let-7e*, and may correspond to the formation of the *rG4-let-7e*-quadruplex structure. To support this point, we introduced G-to-A mutations in *pri-let-7e* at positions that are expected to destabilize the *rG4-let-7e*-quadruplex (Figure S6E). We found that G-to-A mutants display single-phase melting curves with transitions >70°C (Figure S6F). This result demonstrates that G-to-A mutations in *pri-let-7e* impede G-quadruplex formation.

We hypothesized that m7G may affect the stability of G-quadruplexes *in vivo* by disrupting the N7 H-bonds and Hoogsteen base pairing while preserving Watson-Crick base pairing (Figure 6A). To support this hypothesis, we used 7-deaza-deoxyguanosine (DAG) as a mimic of m7G, since synthesis of m7G-containing oligonucleotides is currently unavailable and because, like m7G, DAG weakens secondary structures supported by Hoogsteen base pairing (Figure 6A; Römmler et al., 2013). We found that the *rG4-let-7e* quadruplex structure containing a single G-to-DAG substitution at the G11 position was significantly less stable than the WT sequence (Figure S6C). We then generated G-to-DAG mutant versions of *pri-let-7e* (Figure 6B). While the D1 and D2 oligonucleotides displayed two G-to-DAG mutations at G4-G5 and G11-G12, respectively, one oligonucleotide bears a single G-to-DAG mutation at position G11. Denaturation experiments followed by CD spectroscopy were performed to assess the quadruplex:stem-loop equilibrium within *pri-let-7e* and the contribution of each guanosine to *pri-let-7e* folding (Figures 6C and 6D). We observed that mutation of G11 (in both D2 and G11 mutants) affects the folding of *pri-let-7e* by shifting the structural equilibrium toward the canonical stem-loop structure. In contrast, mutating G4 and G5 (D1 mutant) did not significantly affect the structural equilibrium. These results suggest that the methylation of G11 favors the stem-loop structure of *let-7e*.

We next used the DAG-containing *pri-let-7e* hairpin oligonucleotides to establish whether the induced change in structure affects the processing of the precursor RNAs *in vivo*. We transfected either WT or the DAG-containing oligonucleotides into A549 cells and we measured the levels of mature *let-7e-5p* by

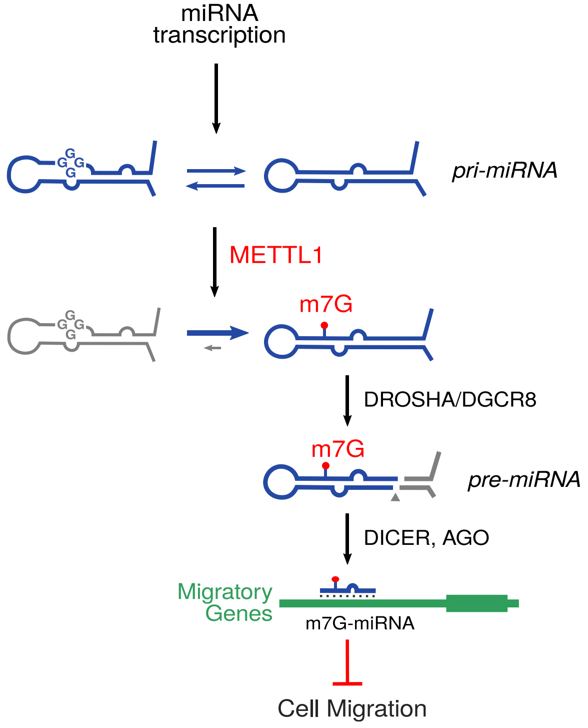

Figure 7. Role of m7G in miRNA Biogenesis
Proposed model of the role of METTL1-mediated m7G in promoting miRNA processing and suppressing migration phenotype.

qRT-PCR 72 h after transfection. Furthermore, we transfected D2 and WT oligonucleotides into A549 *METTL1* knockdown cells and measured the levels of HMGA2 by immunoblotting. In agreement with the biophysical observations, the D2 oligonucleotides, containing G11 DAG, were more efficiently processed than either WT *let-7e* or D1 oligonucleotides (Figure 6E). Finally, the D2 oligonucleotide effectively rescued HMGA2 expression in the absence of METTL1, whereas the WT did not (Figure 6F). Overall, our data suggest that the methylation of *pri-let-7e* at G11 promotes its processing via disruption of local G-quadruplex structures.

DISCUSSION

In the present study, we use two independent unbiased techniques to demonstrate that a subset of miRNAs harbors internal m7G modification. We find that these are functionally related, tumor-suppressive miRNAs, and they include the *let-7* family. We show that m7G promotes the processing of their precursor RNAs and that METTL1-dependent methylation is required to suppress cellular migration. Furthermore, we developed a new MS approach to identify m7G within a sequence-specific context using RNA purified from cells. This allowed us to pinpoint the modification to G11 of the mature *let-7e-5p*. We show that *pri-let-7e* can adopt two alternative conformations, which are consistent with a G-quadruplex structure and a canonical stem-loop. The G11 position has previously been implicated in the formation of a G-quadruplex structure (Pandey et al., 2015), and we confirm the presence of this G-quadruplex in *let-7e.* We show that the substitution of G11 with DAG affects the quadruplex:stem-loop equilibrium within *pri-let-7e* and mimics the effects due to m7G.

The presence of m7G in miRNAs strongly correlates with their predicted tendency to adopt G-quadruplex structures. Such structures are known to be inhibitory to miRNA processing (Mirihana Arachchilage et al., 2015; Pandey et al., 2015), which is consistent with our findings that the G-quadruplex motif in *let-7e* overlaps the DROSHA cleavage site. Our data suggest a model in which METTL1-mediated deposition of m7G within G-rich regions destabilizes G-quadruplexes, thereby promoting their processing from pri- to pre-miRNA (Figure 7).

While this manuscript was under revision, Gregory and colleagues (Lin et al., 2018) reported that *METTL1* knockout mouse embryonic stem cells possess defective mRNA translation at a global level. In our system, by using an inducible knockdown approach to reduce the levels of METTL1, we did not dramatically affect the levels of m7G in tRNAs (Figure S2G). This is consistent with our ribosome profiling results in normal versus METTL1-depleted cells, which showed no significant effect on overall translation (Figure S2K). Therefore, our inducible approach allowed us to dissect new, orthogonal, m7G-dependent pathways, uncoupling them from the effects of tRNA.

G-quadruplexes and other alternative structures involving non-Watson-Crick base pairing have been described in other classes of RNA, including mRNA (Bugaut and Balasubramanian, 2012); there they are proposed to induce ribosome stalling, thereby inhibiting translation (Endoh et al., 2013). We speculate that m7G represents a general way of destabilizing such structures, counteracting their effects. Thus, a comprehensive understanding of the m7G modification pathways will be instrumental in deciphering the roles that Hoogsteen-based structures play in physiological and pathological settings.

Only two other miRNA methylations have been identified in miRNAs, m6A and 5′-methyl phosphate (Alarcón et al., 2015; Xhemalce et al., 2012). However, these two modifications show features that are different from the m7G features characterized here. On the one hand, m6A enhances processing of many, if not all, miRNAs in breast cancer cells, and can be regarded as a general mechanism; this methylation promotes the binding of DROSHA to the primary miRNA. On the other hand, 5′-methyl phosphate represses the processing of *miR-145* by inhibiting the binding of DICER to the pre-miRNA. Here, we show that m7G promotes miRNA processing in a unique manner, by directly affecting the secondary structure of a specific set of pri-miRNAs that share a common functional signature: suppression of cell migration.

The model proposed here could represent a widespread molecular mechanism to safeguard the levels and activity of important tumor-suppressive G-rich miRNAs. In this scenario, the formation of G-quadruplexes is a “side effect” of the miRNA sequence, and the m7G pathway is required to maintain pre-miRNAs in a functional state. Alternatively, but not mutually exclusively, the presence of G-quadruplexes itself may represent a novel additional layer of regulation to control functionally related miRNAs, which, for example, inhibit cell migration.

Many mechanisms control cell migration and tumor invasiveness, and one of the most important drivers is the AKT oncogenic signaling pathway (Irie et al., 2005). Notably, AKT has been shown to directly phosphorylate METTL1 to inhibit its enzymatic activity (Cartlidge et al., 2005). Given the findings presented here, it is likely that the hyperactivation of AKT in cancer would reduce the levels of m7G-containing tumor-suppressive miRNAs, including the *let-7* miRNA family. This family in particular inhibits the progression and invasiveness of numerous tumors, including lung cancer, by regulating the expression of key oncogenes such as *RAS*, *MYC*, and *HMGA2* (Balzeau et al., 2017). The control of *let-7* family members by the m7G pathway may represent a common mechanism to modulate their expression and therefore activity. Beyond cancer, *let-7* is implicated in neurodegenerative diseases, such as Alzheimer's disease, in which it is significantly upregulated (Lehmann et al., 2012). Furthermore, low levels of *let-7* have been shown to improve tissue repair through reprogramming cellular metabolism (McDaniel et al., 2016; Shyh-Chang et al., 2013). Therefore, direct targeting of METTL1 could represent a valid and unexplored therapeutic strategy in these pathological contexts.

This report identifies the m7G pathway as a novel regulator of miRNA function. Considering the interest in miRNA as targets and tools in therapeutic intervention (Chakraborty et al., 2017), our findings could be exploited in many miRNA-related disease settings to open up new therapeutic avenues.

STAR★METHODS

Detailed methods are provided in the online version of this paper and include the following:

- KEY RESOURCES TABLE
- CONTACT FOR REAGENT AND RESOURCE SHARING
- EXPERIMENTAL MODEL AND SUBJECT DETAILS
  - ○ Cell lines
  - ○ Lentiviral vector preparation and cell transduction
  - ○ Generation of conditional knockdown cells
- METHOD DETAILS
  - ○ BoRed-Seq and m7G-RIP-Seq
  - ○ RNA immunoblots and dot blots
  - ○ Global gene expression profiling
  - ○ RT-qPCR
  - ○ Western blotting
  - ○ Migration assays
  - ○ Proliferation assays
  - ○ Mature miRNA/hairpin transfection
  - ○ Luciferase assay
  - ○ METTL1 rescue experiments
  - ○ METTL1 UV-CLIP
  - ○ Expression and purification of recombinant METTL1/WDR4 (Evotec)
  - ○ *In vitro* RNA methylation assays
  - ○ Mass spectrometry analysis of RNA nucleoside m7G modification
  - ○ Mature miRNA isolation
  - ○ Context-specific mass spectrometry analysis of miRNA m7G modification
  - ○ Preparation of naive and *in vitro* methylated [α-^{32}P]-pri-let-7e
  - ○ Isolation of DROSHA by IP
  - ○ *In vitro* miRNA Processing Assays
  - ○ Circular dichroism spectroscopy
  - ○ Polysome fractionation
- QUANTIFICATION AND STATISTICAL ANALYSIS
  - ○ Bioinformatic analysis of smallRNA sequencing
  - ○ Global gene expression analysis
  - ○ Bioinformatic prediction of G-quadruplexes
  - ○ Statistical analysis
- DATA AND SOFTWARE AVAILABILITY
  - ○ Data Resource

SUPPLEMENTAL INFORMATION

Supplemental Information can be found online at https://doi.org/10.1016/j.molcel.2019.03.040.

ACKNOWLEDGMENTS

The authors would like to thank Dr. Tommaso Leonardi for providing bioinformatics advice regarding small RNA-seq analysis and Dr. Pieter van Delft for insightful discussions. We also thank Dr. Gonzalo Millan-Zambrano and Dr. Angela Rubio for help with polysome fractionation. Work in the Kouzarides laboratory is supported by grants from Cancer Research UK (grant reference RG72100) and the ERC (project number 268569), in addition to benefiting from core support from the Wellcome Trust (core grant reference WT203144) and Cancer Research UK (grant reference C6946/A24843). I.B. is funded by a Kay Kendall Leukaemia Fund project grant (grant reference RG88664) and Cancer Research UK (grant reference RG86786). The Balasubramanian lab is supported by an ERC Advanced grant (337778) and by core funding from Cancer Research UK. R.B. and M.d.O. are funded by a Horizon 2020 grant (grant reference 732678).

AUTHOR CONTRIBUTIONS

Conceptualization, L.P., I.B., A.J.B., and T.K.; Methodology, L.P., I.B., A.H., and B.A.; Software & Formal Analysis, L.P., P. Murat, and S.C.R.; Investigation, L.P., I.B., A.H., B.A., N.W., P. Murat, P. Mach, R.B., V.M., and A.A.; Resources, M.d.O.; Writing – Original Draft, A.J.B.; Writing – Review & Editing, L.P., I.B., A.J.B., S.B., and T.K.; Visualization, L.P. and I.B.; Supervision & Project Administration, A.J.B., S.B., and T.K.; Funding Acquisition, I.B., A.J.B., and T.K.

DECLARATION OF INTERESTS

T.K. is a co-founder of Abcam and Storm Therapeutics, Cambridge, UK. A.H., B.A., and N.W. are employees of Storm Therapeutics, Cambridge, UK. S.B. is a founder of and shareholder in Cambridge Epigenetix, Cambridge, UK.

Received: April 18, 2018
Revised: March 6, 2019
Accepted: March 27, 2019
Published: April 25, 2019

REFERENCES

Alarcón, C.R., Lee, H., Goodarzi, H., Halberg, N., and Tavazoie, S.F. (2015). N6-methyladenosine marks primary microRNAs for processing. Nature *519*, 482–485.

Alexandrov, A., Martzen, M.R., and Phizicky, E.M. (2002). Two proteins that form a complex are required for 7-methylguanosine modification of yeast tRNA. RNA *8*, 1253–1266.

Balzeau, J., Menezes, M.R., Cao, S., and Hagan, J.P. (2017). The LIN28/let-7 pathway in cancer. Front. Genet. *8*, 31.

Bao, X., Guo, X., Yin, M., Tariq, M., Lai, Y., Kanwal, S., Zhou, J., Li, N., Lv, Y., Pulido-Quetglas, C., et al. (2018). Capturing the interactome of newly transcribed RNA. Nat. Methods *15*, 213–220.

Bartel, D.P. (2009). MicroRNAs: target recognition and regulatory functions. Cell *136*, 215–233.

Bartel, D.P. (2018). Metazoan microRNAs. Cell *173*, 20–51.

Bedrat, A., Lacroix, L., and Mergny, J.-L. (2016). Re-evaluation of G-quadruplex propensity with G4Hunter. Nucleic Acids Res. *44*, 1746–1759.

Bolger, M., Lohse, M., and Usadel, B. (2014). Trimmomatic: a flexible trimmer for Illumina sequence data. Bioinformatics *30*, 2114–2120.

Bugaut, A., and Balasubramanian, S. (2012). 5′-UTR RNA G-quadruplexes: translation regulation and targeting. Nucleic Acids Res. *40*, 4727–4741.

Carell, T., Brandmayr, C., Hienzsch, A., Müller, M., Pearson, D., Reiter, V., Thoma, I., Thumbs, P., and Wagner, M. (2012). Structure and function of noncanonical nucleobases. Angew. Chem. Int. Ed. Engl. *51*, 7110–7131.

Carlile, T.M., Rojas-Duran, M.F., Zinshteyn, B., Shin, H., Bartoli, K.M., and Gilbert, W.V. (2014). Pseudouridine profiling reveals regulated mRNA pseudouridylation in yeast and human cells. Nature *515*, 143–146.

Cartlidge, R.A., Knebel, A., Peggie, M., Alexandrov, A., Phizicky, E.M., and Cohen, P. (2005). The tRNA methylase METTL1 is phosphorylated and inactivated by PKB and RSK in vitro and in cells. EMBO J. *24*, 1696–1705.

Chakraborty, C., Sharma, A.R., Sharma, G., Doss, C.G.P., and Lee, S.-S. (2017). Therapeutic miRNA and siRNA: moving from bench to clinic as next generation medicine. Mol. Ther. Nucleic Acids *8*, 132–143.

Chendrimada, T.P., Gregory, R.I., Kumaraswamy, E., Norman, J., Cooch, N., Nishikura, K., and Shiekhattar, R. (2005). TRBP recruits the Dicer complex to Ago2 for microRNA processing and gene silencing. Nature *436*, 740–744.

Dai, Q., Moshitch-Moshkovitz, S., Han, D., Kol, N., Amariglio, N., Rechavi, G., Dominissini, D., and He, C. (2017). Nm-seq maps 2′-O-methylation sites in human mRNA with base precision. Nat. Methods *14*, 695–698.

Davis, C.A., Hitz, B.C., Sloan, C.A., Chan, E.T., Davidson, J.M., Gabdank, I., Hilton, J.A., Jain, K., Baymuradov, U.K., Narayanan, A.K., et al. (2018). The Encyclopedia of DNA elements (ENCODE): data portal update. Nucleic Acids Res. *46* (D1), D794–D801.

Delatte, B., Wang, F., Ngoc, L.V., Collignon, E., Bonvin, E., Deplus, R., Calonne, E., Hassabi, B., Putmans, P., Awe, S., et al. (2016). RNA biochemistry. Transcriptome-wide distribution and function of RNA hydroxymethylcytosine. Science *351*, 282–285.

Dobin, A., Davis, C.A., Schlesinger, F., Drenkow, J., Zaleski, C., Jha, S., Batut, P., Chaisson, M., and Gingeras, T.R. (2013). STAR: ultrafast universal RNA-seq aligner. Bioinformatics *29*, 15–21.

Dominissini, D., Moshitch-Moshkovitz, S., Schwartz, S., Salmon-Divon, M., Ungar, L., Osenberg, S., Cesarkas, K., Jacob-Hirsch, J., Amariglio, N., Kupiec, M., et al. (2012). Topology of the human and mouse m6A RNA methylomes revealed by m6A-seq. Nature *485*, 201–206.

Dominissini, D., Nachtergaele, S., Moshitch-Moshkovitz, S., Peer, E., Kol, N., Ben-Haim, M.S., Dai, Q., Di Segni, A., Salmon-Divon, M., Clark, W.C., et al. (2016). The dynamic N(1)-methyladenosine methylome in eukaryotic messenger RNA. Nature *530*, 441–446.

Dweep, H., and Gretz, N. (2015). miRWalk2.0: a comprehensive atlas of microRNA-target interactions. Nat. Methods *12*, 697.

Endoh, T., Kawasaki, Y., and Sugimoto, N. (2013). Translational halt during elongation caused by G-quadruplex formed by mRNA. Methods *64*, 73–78.

Fabian, M.R., Sonenberg, N., and Filipowicz, W. (2010). Regulation of mRNA translation and stability by microRNAs. Annu. Rev. Biochem. *79*, 351–379.

Freund, Y., and Schapire, R.E. (1997). A decision-theoretic generalization of on-line learning and an application to boosting. J. Comput. Syst. Sci. *55*, 119–139.

Ghildiyal, M., and Zamore, P.D. (2009). Small silencing RNAs: an expanding universe. Nat. Rev. Genet. *10*, 94–108.

Heo, I., Joo, C., Cho, J., Ha, M., Han, J., and Kim, V.N. (2008). Lin28 mediates the terminal uridylation of let-7 precursor MicroRNA. Mol. Cell *32*, 276–284.

Huppert, J.L., and Balasubramanian, S. (2005). Prevalence of quadruplexes in the human genome. Nucleic Acids Res. *33*, 2908–2916.

Irie, H.Y., Pearline, R.V., Grueneberg, D., Hsia, M., Ravichandran, P., Kothari, N., Natesan, S., and Brugge, J.S. (2005). Distinct roles of Akt1 and Akt2 in regulating cell migration and epithelial-mesenchymal transition. J. Cell Biol. *171*, 1023–1034.

Kozomara, A., and Griffiths-Jones, S. (2014). miRBase: annotating high confidence microRNAs using deep sequencing data. Nucleic Acids Res. *42*, D68–D73.

Kriek, E., and Emmelot, P. (1964). Methylation of deoxyribonucleic acid by diazomethane. Biochim. Biophys. Acta *91*, 59–66.

Kwok, C.K., Marsico, G., Sahakyan, A.B., Chambers, V.S., and Balasubramanian, S. (2016a). rG4-seq reveals widespread formation of G-quadruplex structures in the human transcriptome. Nat. Methods *13*, 841–844.

Kwok, C.K., Sahakyan, A.B., and Balasubramanian, S. (2016b). Structural analysis using SHALiPE to reveal RNA G-quadruplex formation in human precursor microRNA. Angew. Chem. Int. Ed. Engl. *55*, 8958–8961.

Kypr, J., Kejnovská, I., Renciuk, D., and Vorlícková, M. (2009). Circular dichroism and conformational polymorphism of DNA. Nucleic Acids Res. *37*, 1713–1725.

Landthaler, M., Yalcin, A., and Tuschl, T. (2004). The human DiGeorge syndrome critical region gene 8 and Its D. melanogaster homolog are required for miRNA biogenesis. Curr. Biol. *14*, 2162–2167.

Lee, Y.S., and Dutta, A. (2007). The tumor suppressor microRNA let-7 represses the HMGA2 oncogene. Genes Dev. *21*, 1025–1030.

Lee, Y., Jeon, K., Lee, J.-T., Kim, S., and Kim, V.N. (2002). MicroRNA maturation: stepwise processing and subcellular localization. EMBO J. *21*, 4663–4670.

Lee, Y., Ahn, C., Han, J., Choi, H., Kim, J., Yim, J., Lee, J., Provost, P., Rådmark, O., Kim, S., and Kim, V.N. (2003). The nuclear RNase III Drosha initiates microRNA processing. Nature *425*, 415–419.

Lehmann, S.M., Krüger, C., Park, B., Derkow, K., Rosenberger, K., Baumgart, J., Trimbuch, T., Eom, G., Hinz, M., Kaul, D., et al. (2012). An unconventional role for miRNA: let-7 activates Toll-like receptor 7 and causes neurodegeneration. Nat. Neurosci. *15*, 827–835.

Liao, Y., Smyth, G.K., and Shi, W. (2014). featureCounts: an efficient general purpose program for assigning sequence reads to genomic features. Bioinformatics *30*, 923–930.

Lin, S., Liu, Q., Lelyveld, V.S., Choe, J., Szostak, J.W., and Gregory, R.I. (2018). Mettl1/Wdr4-mediated m^7G tRNA methylome is required for normal mRNA translation and embryonic stem cell self-renewal and differentiation. Mol. Cell *71*, 244–255.e5.

Lorenz, R., Bernhart, S.H., Höner zu Siederdissen, C., Tafer, H., Flamm, C., Stadler, P.F., and Hofacker, I.L. (2011). ViennaRNA Package 2.0. Algorithms Mol. Biol. *6*, 26.

Love, M.I., Huber, W., and Anders, S. (2014). Moderated estimation of fold change and dispersion for RNA-seq data with DESeq2. Genome Biol. *15*, 550.

Luo, W., Friedman, M.S., Shedden, K., Hankenson, K.D., and Woolf, P.J. (2009). GAGE: generally applicable gene set enrichment for pathway analysis. BMC Bioinformatics *10*, 161.

Luo, W., Pant, G., Bhavnasi, Y.K., Blanchard, S.G., Jr., and Brouwer, C. (2017). Pathview Web: user friendly pathway visualization and data integration. Nucleic Acids Res. *45* (W1), W501–W508.

Machnicka, M.A., Milanowska, K., Osman Oglou, O., Purta, E., Kurkowska, M., Olchowik, A., Januszewski, W., Kalinowski, S., Dunin-Horkawicz, S., Rother, K.M., et al. (2013). MODOMICS: a database of RNA modification pathways–2013 update. Nucleic Acids Res. *41*, D262–D267.

Mayr, C., Hemann, M.T., and Bartel, D.P. (2007). Disrupting the pairing between let-7 and Hmga2 enhances oncogenic transformation. Science *315*, 1576–1579.

McDaniel, K., Hall, C., Sato, K., Lairmore, T., Marzioni, M., Glaser, S., Meng, F., and Alpini, G. (2016). Lin28 and let-7: roles and regulation in liver diseases. Am. J. Physiol. Gastrointest. Liver Physiol. *310*, G757–G765.

Mirihana Arachchilage, G., Dassanayake, A.C., and Basu, S. (2015). A potassium ion-dependent RNA structural switch regulates human pre-miRNA 92b maturation. Chem. Biol. *22*, 262–272.

Panda, A.C., Martindale, J.L., and Gorospe, M. (2017). Polysome fractionation to analyze mRNA distribution profiles. Bio Protoc. *7*, e2126.

Pandey, S., Agarwala, P., Jayaraj, G.G., Gargallo, R., and Maiti, S. (2015). The RNA stem-loop to G-quadruplex equilibrium controls mature microRNA production inside the cell. Biochemistry *54*, 7067–7078.

Peattie, D.A. (1979). Direct chemical method for sequencing RNA. Proc. Natl. Acad. Sci. USA *76*, 1760–1764.

Piekna-Przybylska, D., Decatur, W.A., and Fournier, M.J. (2008). The 3D rRNA modification maps database: with interactive tools for ribosome analysis. Nucleic Acids Res. *36*, D178–D183.

Römmler, F., Jurk, M., Uhlmann, E., Hammel, M., Waldhuber, A., Pfeiffer, L., Wagner, H., Vollmer, J., and Miethke, T. (2013). Guanine modification of inhibitory oligonucleotides potentiates their suppressive function. J. Immunol. *191*, 3240–3253.

Roundtree, I.A., Evans, M.E., Pan, T., and He, C. (2017). Dynamic RNA modifications in gene expression regulation. Cell *169*, 1187–1200.

Schaefer, M. (2015). RNA 5-methylcytosine analysis by bisulfite sequencing. Methods Enzymol. *560*, 297–329.

Schwartz, S., Bernstein, D.A., Mumbach, M.R., Jovanovic, M., Herbst, R.H., León-Ricardo, B.X., Engreitz, J.M., Guttman, M., Satija, R., Lander, E.S., et al. (2014). Transcriptome-wide mapping reveals widespread dynamic-regulated pseudouridylation of ncRNA and mRNA. Cell *159*, 148–162.

Shyh-Chang, N., Zhu, H., Yvanka de Soysa, T., Shinoda, G., Seligson, M.T., Tsanov, K.M., Nguyen, L., Asara, J.M., Cantley, L.C., and Daley, G.Q. (2013). Lin28 enhances tissue repair by reprogramming cellular metabolism. Cell *155*, 778–792.

Smyth, G.K., and Speed, T. (2003). Normalization of cDNA microarray data. Methods *31*, 265–273.

Smyth, G.K., Michaud, J., and Scott, H.S. (2005). Use of within-array replicate spots for assessing differential expression in microarray experiments. Bioinformatics *21*, 2067–2075.

Tanaka, M., Han, S., Küpfer, P.A., Leumann, C.J., and Sonntag, W.E. (2011). An assay for RNA oxidation induced abasic sites using the aldehyde reactive probe. Free Radic. Res. *45*, 237–247.

van Delft, P., Akay, A., Huber, S.M., Bueschl, C., Rudolph, K.L.M., Di Domenico, T., Schuhmacher, R., Miska, E.A., and Balasubramanian, S. (2017). The profile and dynamics of RNA modifications in animals. ChemBioChem *18*, 979–984.

van Dongen, S., Abreu-Goodger, C., and Enright, A.J. (2008). Detecting microRNA binding and siRNA off-target effects from expression data. Nat. Methods *5*, 1023–1025.

Xhemalce, B., Robson, S.C., and Kouzarides, T. (2012). Human RNA methyltransferase BCDIN3D regulates microRNA processing. Cell *151*, 278–288.

Zhang, H., Hao, Y., Yang, J., Zhou, Y., Li, J., Yin, S., Sun, C., Ma, M., Huang, Y., and Xi, J.J. (2011). Genome-wide functional screening of miR-23b as a pleiotropic modulator suppressing cancer metastasis. Nat. Commun. *2*, 554.

Zueva, V.S., Mankin, A.S., Bogdanov, A.A., and Baratova, L.A. (1985). Specific fragmentation of tRNA and rRNA at a 7-methylguanine residue in the presence of methylated carrier RNA. Eur. J. Biochem. *146*, 679–687.

STAR★METHODS

KEY RESOURCES TABLE

REAGENT or RESOURCE	SOURCE	IDENTIFIER
Antibodies		
Anti-m7G Mouse Monoclonal	MBL (RN017M)	RRID: AB_2725740
Anti-METTL1 Rabbit Polyclonal	Abcam (ab157097)	RRID: AB_2725741
Anti-METTL1 Sheep Polyclonal (for IP)	MRC PPU (588192)	RRID: AB_2725742
Anti-HMGA2 Rabbit Polyclonal	Abcam (ab202387)	RRID: AB_2725743
Anti β-Tubulin Rabbit Polyclonal	Abcam (ab6046)	RRID: AB_2210370
IgG Rabbit Isotype Control	Abcam (ab171870)	RRID: AB_2687657
Anti-FLAG tag Mouse Monoclonal	Sigma-Aldrich (F1804)	RRID: AB_262044
Anti-6xHIS tag Rabbit Polyclonal	Abcam (ab9108)	RRID: AB_307016
Anti-Myc tag Mouse Monoclonal	Abcam (ab32)	RRID: AB_303599
Anti-Rabbit IgG HRP-conjugated Goat Polyclonal	Abcam (ab6721)	RRID: AB_955447
Anti-Mouse IgG HRP-conjugated Goat Polyclonal	Dako (P0447)	RRID: AB_2617137
Bacterial and Virus Strains		
TOP10 Chemically Competent *E. coli*	Thermo Fisher	Cat# C404003
Chemicals, Peptides, and Recombinant Proteins		
1,1,1,3,3,3-Hexafluoropropan-2-ol	Apollo Scientific	Cat# PC0877
1,4-Dithiothreitol	Thermo Fisher	Cat# P2325
2-Mercaptoethanol	Sigma-Aldrich	Cat# M3148
7-methylguanosine triphosphate	Sigma-Aldrich	Cat# M6133
Acetic Acid	Fisher Scientific	Cat# A/0400/PB17
Acetonitrile	Fisher Scientific	Cat# 10407440
Antarctic Phosphatase	NEB	Cat# M0289S
Adenosine 5′-Triphosphate	NEB	Cat# P0756S
Benzonase	Sigma-Aldrich	Cat# E1014-25KU
Boric Acid	BDH Lab. Supplies	Cat# 100584S
Bovine Serum Albumin	NEB	Cat# B9000S
Cacodylic acid	Sigma-Aldrich	Cat# C0125
CapCLIP Acid Pyrophosphatase	CellScript	Cat# C-CC15011H
Chloroform	Fisher Scientific	Cat# C/4960/15
Cycloheximide	Sigma-Aldrich	Cat# C7698
Deoxycholic acid	Sigma-Aldrich	Cat# D2510
DNAse I	Qiagen	Cat# 79254
Doxycycline	Clontech	Cat# 8634-1
Ethylenediaminetetraacetic Acid	Fisher Scientific	Cat# D/0700/53
Ethanol	VWR	Cat# 20820.327
Ethidium Bromide	Sigma-Aldrich	Cat# E1510
Fibronectin	Sigma-Aldrich	Cat# F2006
Formaldehyde 37%	Sigma-Aldrich	Cat# 252549
Formic acid	Fisher Scientific	Cat# 10596814
G418 disulphate	Melford	Cat# G0175
Glycogen	Roche	Cat# 10901393001
HEPES	Melford	Cat# B2001
KCl	Sigma-Aldrich	Cat# P9333
LiCl	Sigma-Aldrich	Cat# L-4408

(*Continued on next page*)

Continued

REAGENT or RESOURCE	SOURCE	IDENTIFIER
Lipofectamine 2000	Thermo Fisher	Cat# 11668019
Lithium Hydroxide	Sigma-Aldrich	Cat# 442410
Methanol	VWR	Cat# 20846.326
METTL1/WDR4 Recombinant complex	Evotec	(*Ad hoc* preparation)
$MgCl_2$	Sigma-Aldrich	Cat# M-0250
N-(aminooxyacetyl)-N'-(D-Biotinoyl) hydrazine	Thermo Fisher	Cat# A10550
NaCl	Sigma-Aldrich	Cat# S7653
NheI	NEB	Cat# R3131S
NotI	NEB	Cat# R3189S
NP40	Sigma-Aldrich	Cat# I3021
Phenylmethanesulfonyl fluoride	Sigma-Aldrich	Cat# P7626
Phosphocreatine disodium salt hydrate	Sigma-Aldrich	Cat# P7936
Phosphodiesterase 1	Sigma-Aldrich	Cat# P3243-1VL
Polybrene	Sigma-Aldrich	Cat# 107689
Protease Inhibitor Complete tablets, EDTA-free	Roche	Cat# 11836170001
Proteinase K	NEB	Cat# P8107S
Puromycin	Invivogen	Cat# ant-pr-1
Qiazol	Qiagen	Cat# 79306
RNAse A	Thermo Fisher	Cat# EN0531
RnaseOUT Ribonuclease Inhibitor	Thermo Fisher	Cat# 10777019
RNAsin Plus Ribonuclease Inhibitor	Promega	Cat# N2611
S-Adenosyl-Methionine	NEB	Cat# B9003S
Sodium borohydride	Sigma-Aldrich	Cat# 480886
Sodium dodecyl sulfate	ICN	Cat# 811030
Sodium hydroxide	Sigma-Aldrich	Cat# S8045
Spermidine trihydrochloride	Sigma-Aldrich	Cat# S2501
Sucrose	Fisher Scientific	Cat#S/8600/60
Triethylamine	VWR	Cat# 84883.180
TRIS Base	Melford	Cat# T60040-1000.0
Triton X-100	Sigma-Aldrich	Cat# X100
Tween-20	Sigma-Aldrich	Cat# P1379
Uridine-^{13}C9,^{15}N2 5′-triphosphate	Sigma-Aldrich	Cat# 645672-1MG
XbaI	NEB	Cat# R0145S
[α-^{32}P]-GTP / 3000Ci/mmol - 10mCi/ml	Perkin-Elmer	Cat# BLU006H250UC
Critical Commercial Assays		
Agilent SurePrint G3 Human Gene Expression Array v3 (8x60K)	Agilent Technologies	Cat# G4851C
Dual-Glo Luciferase Assay System	Promega	Cat# E2920
ECL Prime detection reagent kit	GE Healthcare	Cat# RPN2232
Fast SybrGreen PCR mastermix	Applied Biosystems	Cat# 4385612
High-capacity cDNA reverse transcription kit	Applied Biosystems	Cat# 4368814
Low Input QuickAmp Labeling Kit, One-Color	Agilent Technologies	Cat# 5190-2305
miRNEasy mini kit	Qiagen	Cat# 217004
miScript II RT kit	Qiagen	Cat# 218161
NEBNext SmallRNA kit	NEB	Cat# E7300S
Qubit dsDNA HS Assay Kit	Thermo Fisher	Cat# Q32851
Qubit RNA HS Assay	Thermo Fisher	Cat# Q32852
RNA Clean & Concentrator - 25	Zymo	Cat# R1017

(*Continued on next page*)

Continued

REAGENT or RESOURCE	SOURCE	IDENTIFIER
RNA Clean & Concentrator - 5	Zymo	Cat# R1013
RNeasy MinElute Cleanup Kit	Qiagen	Cat# 74204
SuperScript III Reverse Transcriptase	Thermo Fisher	Cat# 18080044
Tapestation RNA ScreenTape	Agilent Technologies	Cat# 5067-5576
TaqMan Advanced miRNA cDNA Synthesis Kit	Thermo Fisher	Cat# A28007
TaqMan Fast Advanced Master Mix	Applied Biosystems	Cat# 4444556
TaqMan Advanced miRNA Assay (A25576)	Thermo Fisher	Listed in Table S8
TranscriptAid T7 High Yield Kit	Thermo Fisher	Cat# K0441
Universal ProbeLibrary (4683633001)	Roche	Listed in Table S8
Deposited Data		
METTL1 Knockdown Expression Microarray data	This study	GEO: GSE112180
BoRed-seq and m7G-RIP-seq in A549	This study	GEO: GSE112181
m7G-RIP-Seq in Caco-2	This study	GEO: GSE120454
m7G-RIP-Seq in A549 METTL1 Knockdown	This study	GEO: GSE120455
Unprocessed imaging data	This study	https://data.mendeley.com/datasets/yscng45zgj/1
Experimental Models: Cell Lines		
HEK-293T (Human embryonic kidney)	ATCC	RRID: CVCL_0063
A549 (Human lung adenocarcinoma)	ATCC	RRID: CVCL_0023
Caco-2 (Human colorectal adenocarcinoma)	ATCC	RRID: CVCL_0025
Oligonucleotides		
DNA and RNA oligonucleotides are listed in Table S8	This study	N/A
GFP MISSION esiRNAs	Sigma-Aldrich	Cat# EHUEGFP-50UG
METTL1 MISSION esiRNAs	Sigma-Aldrich	Cat# EHU076851-50UG
miRIDIAN Control miRNA mimic	Dharmacon	Cat# CN-001000-01-05
miRIDIAN *hsa-let-7e-5p* miRNA mimic	Dharmacon	Cat# C-300479-05-0002
Recombinant DNA		
Hmga2-Luc-m7	Addgene	#14788
Hmga2-Luc-wt	Addgene	#14785
PAX2	Addgene	#12260
pcDNA3-pri-let-7e	Addgene	#51380
pcDNA4/TO/cmycDrosha	Addgene	#10828
pHIV-ZsGreen	Addgene	#18121
pLKO-TETon-Puro	Addgene	#21915
pMD2.G	Addgene	#12259
pMirGlo	Promega	Cat# E1330
Software and Algorithms		
Bioconductor	https://www.bioconductor.org/	N/A
DESeq2	Love et al., 2014	N/A
FastQC	https://github.com/s-andrews/FastQC	N/A
featureCounts	Liao et al., 2014	N/A
G4Hunter	Bedrat et al., 2016	N/A
Gage	Luo et al., 2009	N/A
gbm	Freund and Schapire, 1997	N/A
limma	Smyth et al., 2005	N/A
miRWalk 2.0	Dweep and Gretz, 2015	N/A
Pathwiew	Luo et al. 2017	N/A

(*Continued on next page*)

Continued		
REAGENT or RESOURCE	SOURCE	IDENTIFIER
Quadparser	Huppert and Balasubramanian, 2005	N/A
R statistical environment	https://www.r-project.org/	N/A
RNAfold	Lorenz et al. 2011	N/A
STAR	Dobin et al., 2013	N/A
Sylamer	van Dongen et al., 2008	N/A
Trimmomatic	Bolger et al., 2014	N/A
Feature Extraction Software	Agilent Technologies	G4463AA
XCalibur	Thermo Fisher	OPTON-30487
Other		
ACQUITY UPLC HSS T3 Column	Waters Corp	Cat# 186005614
Amicon 30kDa MWCO spin-column	Merck Millipore	Cat# Z717185
Amersham Hybond-C Extra Nitrocellulose membrane	GE Healthcare	Cat# RPN203D
Amersham Hybond-N+ Nylon Membrane	GE Healthcare	Cat# RPN2020B
Amersham Hyperfilm HS autoradiography film	GE Healthcare	Cat# 28906836
Bradford assay	Bio-Rad	Cat# 5000006
Denhart's solution	Thermo Fisher	Cat# 750018
Dulbecco's Modified Essential Medium	Gibco	Cat# 41965-039
Dynabeads MyOne Streptavidin C1	Thermo Fisher	Cat# 65001
Dynabeads Protein G	Thermo Fisher	Cat# 10004D
Eagle's Modified Essential Medium	Sigma-Aldrich	Cat# M2279
Fetal Bovine Serum	Gibco	Cat# 10270-106
Illustra MicroSpin G-25 spin column	GE Healthcare	Cat# 27532501
Migration assay transwell inserts (8 μm)	Corning	Cat# 3422
Novex TBE 6% precast gel	Thermo Fisher	Cat# EC6265BOX
Novex TBE-Urea 10% precast gel	Thermo Fisher	Cat# EC6875BOX
Novex TBE-Urea 15% precast gel	Thermo Fisher	Cat# EC6885BOX
Novex TBE-Urea 6% precast gel	Thermo Fisher	Cat# EC6865BOX
Novex TBE-Urea Sample Buffer	Thermo Fisher	Cat# LC6876
Penicillin/Streptomycin/Glutamine	Gibco	Cat# 10378016
Protein G Sepharose 4 Fast Flow beads	GE Healthcare	Cat# 17-0618-01

CONTACT FOR REAGENT AND RESOURCE SHARING

Queries and reagent requests may be directed and will be fulfilled by the lead contact, Tony Kouzarides (tony.kouzarides@gurdon.cam.ac.uk).

EXPERIMENTAL MODEL AND SUBJECT DETAILS

Cell lines

HEK293T (RRID:CVCL_0063) and A549 cells (RRID:CVCL_0023) were cultured in DMEM (Invitrogen), supplemented with 10% fetal bovine serum (FBS) and 1% penicillin/streptomycin/glutamine (PSQ). Caco-2 cells (RRID:CVCL_0025) were cultured in Eagle's Minimum Essential Medium, supplemented with 20% FBS and 1% PSQ. Cell lines were obtained from the ATCC and tested negative for mycoplasma contamination. Human cell lines used are not listed in the cross-contaminated or misidentified cell lines database curated by the International Cell Line Authentication Committee (ICLAC).

Lentiviral vector preparation and cell transduction

For virus production, HEK293T cells were transfected with the lentiviral vector pLKO-TETon-Puro for *METTL1* knockdown, or Zs-Green-HIV for *METTL1* rescue experiments, together with the packaging plasmids PAX2 (Addgene Plasmid #12260) and pMD2.G (Addgene Plasmid #12259) at a 1:1.5:0.5 ratio using Lipofectamine 2000 reagent (Invitrogen) according to the

manufacturer's instructions. Supernatants were harvested 48 h and 72 h after transfection. Cells (5×10^5) were mixed in 2 ml viral supernatant supplemented with 8 μg/ml polybrene (Millipore), followed by spinfection (60 min, 900 g, 25°C) and further incubated overnight at 37°C. For *METTL1* knockdown experiments, cells were replated in fresh medium containing 1 μg/ml puromycin and kept in selection medium for 7 days. For METTL1 rescue experiments, GFP+ cells were isolated using a SONY SH800 cell sorter 48 h after infection.

Generation of conditional knockdown cells

A549 or Caco-2 cells were infected with pLKO-TETon-Puro lentiviral vectors (Addgene Plasmid #21915) expressing shRNAs against the coding sequence of human *METTL1* or a scrambled control as described above. The shRNA sequences are listed in Table S8. shRNA was induced by treatment with 200 ng/ml doxycycline for the indicated times.

METHOD DETAILS

BoRed-Seq and m7G-RIP-Seq

The detailed protocol of all the procedures required to perform BoRed-Seq and m7G RNA immunoprecipitation experiments on small RNAs is described in Methods S1. Single-end 50-bp stranded smallRNA libraries were prepared using the NEBNext SmallRNA kit (NEB) according to the manufacturer's recommendations and sequenced on a HiSeq 4000 (Illumina).

RNA immunoblots and dot blots

RNA was resolved by denaturing polyacrylamide gel electrophoresis using Novex TBE-urea 15% precast gels (Thermo Fisher). Equal loading was checked by staining with ethidium bromide (Sigma-Aldrich), then RNA was transferred to a nylon membrane (Amersham Hybond-N+, GE Healthcare) by wet electro-blotting in TBE (45 min at 400 mA).

For dot-blot analysis, input RNA or RNA immunoprecipitated with either anti-m7G or isotypic non-specific antibodies was spotted onto a nitrocellulose membrane and UV cross-linked at 254 nm (120 mJ/cm^2). The membranes were blocked in Denhart's solution (1% Ficoll, 1% polyvinylpyrrolidone, 1% bovine serum albumin; Thermo Fisher) for 1 h at room temperature and incubated with m7G antibody for 1 h at room temperature. Signal was detected using HRP conjugated secondary antibodies and ECL (GE Healthcare) and developed on a Chemidoc MP machine (BioRad).

Global gene expression profiling

Cells were lysed in Qiazol (QIAGEN) and total RNA was extracted with miRNeasy mini kit (QIAGEN). RNA quality was assessed using an Agilent Tapestation RNA; 50 ng of RNA were labeled with Low Input QuickAmp Labeling Kit, One-Color (Agilent Technologies), purified and hybridized overnight onto an Agilent SurePrint G3 Human Gene Expression Array v3 (8x60K) before detection according to the manufacturer's instructions. An Agilent DNA microarray scanner (model G2505C) was used for slide acquisition.

RT-qPCR

Cells were lysed in Qiazol (QIAGEN) and total RNA was purified using the miRNEasy mini kit (QIAGEN) according to the manufacturer's instructions. For mRNA detection, 1 μg of purified total RNA was reverse transcribed using the high-capacity cDNA reverse transcription kit (Applied Biosystems). To quantify gene expression, we used probes from Universal ProbeLibrary (UPL; Roche) with TaqMan Fast Advanced Master Mix (Thermo Fisher).

For specific pri- and pre-miRNA quantification, we size-fractionated large (> 200nt, containing the pri-miRNAs) and small RNAs (< 200nt, containing pre-miRNAs) using RNA Clean & Concentrator 5 column kits (Zymo), as per the manufacturer's instructions. Pri-miRNAs were reverse transcribed using the High-Capacity cDNA reverse transcription kit (Applied Biosystems), which employs random nonamer priming and favors long molecules. Pre-miRNAs were reverse transcribed with miScript II RT kit (QIAGEN), which is more efficient on short RNAs. Primers were designed to anneal either within the stem loop (pre-miRNAs) or to overlap the DROSHA cleavage sites (pri-miRNAs). Both pri- and pre-miRNAs were quantified using Fast SybrGreen PCR mastermix (Applied Biosystems) according to the manufacturer's instructions.

For mature miRNA detection, total RNA was reverse transcribed and amplified using the Taqman Advanced miRNA cDNA Synthesis Kit from Thermo Fisher Scientific. The levels of specific miRNAs were measured with Taqman advanced miRNA Assays from Thermo Fisher Scientific. All the RT-qPCR experiments were run on an ABI 7900 real-time PCR machine (Applied Biosystems). *GAPDH* and *RNY1* were used as housekeeping genes for RT-qPCR normalization of long and small RNAs, respectively. Primer sequences, UPL probe numbers and assay IDs are listed in Table S8.

Western blotting

For total cell protein extraction and western blot analysis, cells were lysed in a buffer containing 50 mM Tris-HCl pH 7.5, 150 mM NaCl, 1% Triton X-100, 0.5% deoxycholic acid, 0.1% sodium dodecyl sulfate, Complete protease inhibitor cocktail (Roche) and cleared by centrifugation. The protein concentration was determined by Bradford assay (Bio-Rad). Proteins were resolved by sodium dodecyl sulfate-polyacrylamide gel electrophoresis and transferred to a nitrocellulose membrane (Hybond-C Extra, GE Healthcare). Membranes were blocked with 5% milk proteins in TBST (50 mM Tris-HCl pH 7.6, 150 mM NaCl, 0.05% Tween-20), and probed with

primary antibodies overnight. Membranes were then washed three times with TBST (15 min each) and probed with a HRP-conjugated secondary anti-rabbit antibody for 1 h. After three more washes, signal was detected using HRP conjugated secondary antibodies and ECL (GE Healthcare) and developed on a BioRad Chemidoc MP machine.

Migration assays

For transwell migration assays, the lower surface of the transwell inserts (8 μm; Corning) was coated with human recombinant fibronectin (1 μg/ml, 1 h at RT; Sigma-Aldrich). A549 cells were serum-starved overnight and seeded (3×10^4) in serum-free medium on transwell inserts. After 7 h incubation in wells containing DMEM+20% FBS, inserts were stained with crystal violet and cells on the lower surface were counted (blindly).

Proliferation assays

4 days after shRNA induction, 10^5 A549 *METTL1* knockdown or Ctrl cells were plated in each well of 6-well cell culture plates in normal culture medium. Cells were counted daily for the following three days using a Countess II cell counter (Thermo Fisher).

Mature miRNA/hairpin transfection

20 pmol of *hsa-let-7e-5p* miRNA mimic or control miRNAs (miRIDIAN, Dharmacon) were transfected into 2.5×10^5 A549 cells using 2 μL of Lipofectamine 2000 (Thermo Fisher). 10 pmol of *pri-let-7e* hairpins (Integrated DNA Technologies) were transfected into 1.5×10^5 A549 cells using the same amount of Lipofectamine as above.

Luciferase assay

A dual luciferase reporter harboring the 3′-UTR of mouse *Hmga2* was generated extracting the XbaI-NotI digestion fragments from either Hmga2-Luc-wt or Hmga2-Luc-m7 (Mayr et al., 2007; Addgene plasmids #14785 and #14788) and subcloning them into pMirGlo (Promega). In order to generate stable reporter lines, A549 cells were transfected with the pMirGlo-hmga2(3′-UTR) constructs. 24 h after transfection, cells were selected using 300 μg/ml G418 for 7 days. Subsequently, cells were transfected with 20 pmol of MISSION esiRNAs (Sigma-Aldrich) against human *METTL1* or GFP. 24 h after transfection, Firefly and Renilla luciferase activities were measured using the Dual-Luciferase Reporter Assay System (Promega) on a CLARIOstar microplate reader (BMG Labtech).

METTL1 rescue experiments

cDNA was obtained by reverse transcription of A549 RNA with Superscript III (Thermo Fisher), then the *METTL1* full-length coding sequence was amplified by PCR and cloned into pHIV-ZsGreen plasmid (Addgene plasmid #18121) using restriction sites XbaI and NotI. In order to generate an shRNA-resistant *METTL1* sequence, synonymous substitutions were introduced in the codons corresponding to shRNA binding sites by long DNA fragment synthesis (GeneArt Strings; Thermo Fisher) of the N-terminal portion of *METTL1* (up to NheI site). The *in vitro* synthesized fragment was swapped into pHIV-ZsGreen-METTL1 using restriction sites NotI and NheI. The same approach was combined to codon mutagenesis to generate the catalytically inactive METTL1 variant EIR/AAA (amino acids 107-109, that form the SAM-binding pocket of the enzyme; see Figure S2I). Primer and long oligonucleotide sequences are listed in Table S8.

METTL1 UV-CLIP

Adherent cells in a 15 cm dish were rinsed twice in ice-cold PBS, cross-linked at 254 nm (120 mJ/cm^2), scraped and lysed on ice for 10 min in 1 mL of fresh lysis buffer (25 mM Tris-HCl pH 8.0, 150 mM NaCl, 2 mM $MgCl_2$, 0.5% NP-40, 5 mM DTT) supplemented with protease inhibitors (Complete tablets, Roche) and RNase inhibitors (RNaseOUT; Thermo Fisher). Lysates were vortexed, then centrifuged at max speed at 4°C for 5 min. The supernatants were used in immunoprecipitation (IP) assay with 5 μg of anti METTL1-antibody (sheep polyclonal obtained by MRC Protein Phosphorylation Unit, #588192; Cartlidge et al., 2005) or control isotypic IgG at 4°C for 90 min, with rotation. 80 μL of Protein G Dynabeads (Invitrogen) per IP reaction were rinsed 2 times with lysis buffer and blocked with 1 mg/ml BSA (NEB) for 2 h at 4°C. Beads were resuspended in 100 μL lysis buffer, then added to the IP tube. The reaction was incubated at 4°C for 2 h with rotation. Beads were then washed twice with high-salt buffer (25 mM Tris-HCl pH 8.0, 500 mM NaCl, 1 mM $MgCl_2$, 1% NP-40, 5 mM DTT) and three times with lysis buffer. Immunoprecipitated samples were treated with DNase I (QIAGEN) followed by digestion with Proteinase K (NEB). RNA was purified using an RNeasy MinElute column kit (QIAGEN).

Expression and purification of recombinant METTL1/WDR4 (Evotec)

The constructs EV4866 (his-METTL1) and EV4868 (flag-WDR4) were cloned into plasmid pTriIJ-HV (Evotec). Recombinant virus was produced by co-transfecting transfer plasmid DNA and bacmid DNA in insect cells. 100 ng bacmid DNA and 500 ng transfer plasmid DNA were mixed with 2 μl Cellfectin II transfection reagent (Invitrogen) in 200 μl TC100 media (Sigma) and incubated at room temperature for 2-3 h. Sf21 insect cells, grown to 80%–90% confluency in 24 well plates were washed with TC100 before adding 0.2 mL TC100 and 0.2 mL co-transfection mix. After overnight incubation, 0.6 mL Sf900 II SFM media containing 5 μg/ml gentamicin was added and the cells were incubated at 27°C for six days with humidity. The cells were observed under an inverted microscope

and compared to the mock-transfected control. BluoGal (2%) was added to the LacZ positive control well and blue coloration was observed within 1 h. Following confirmation of successful transfection, the medium containing the recombinant virus (P0) was harvested into a sterile deep well block and stored in the dark at 4°C. P1 BIICs (baculovirus-infected insect cells) were amplified in a sterile 24-deep well block by infecting Sf21 cells grown in Sf900 II SFM media containing 5 μg/ml gentamicin with P0 virus. The infected cultures were incubated for 72-120 h at 27°C with shaking at 360 rpm. P1-BIICs were harvested by centrifugation of the block and virus supernatant was removed to a fresh block and stored at 4°C. The cells were then re-suspended in freezing mix (Sf900 II + 10% heat inactivated FBS + 10% DMSO) and frozen gradually to −80°C, in the block. Working P2-BIICs were amplified by infecting Sf21 cells grown in shake flasks at MOI-0.1 using P1-BIICs and incubated for 72 h. P2-BIICs were harvested by centrifugation and infected cells were resuspended in freezing media and stored at −80°C. Sf21 cells grown in Sf900 II SFM media plus 5 μg/ml gentamicin were infected with both EV4866 and EV4868 P2 BIICs at an MOI of 2 (1+1). The infected culture was incubated for 72 h at 27°C with shaking at 110 rpm, before harvesting by centrifugation and storing at −80°C. Thawed cells were lysed in 25 mM Tris-HCl pH 8.0, 300 mM NaCl, 1 mM TCEP, 5% glycerol, 0.25% CHAPS supplemented with Complete EDTA-free protease inhibitor tablets (Roche). Samples were homogenized for 20-30 s with an IKA Ultra-Turrax and sonicated in a Branson probe sonicator (cycles of 30 s on, 30 s off for 5 min at 40% amplitude). Samples were centrifuged at 45000 rpm for 50 min to remove insoluble material. Purification was carried out by sequential Ni-affinity and size-exclusion chromatography on an ÄKTA Xpress system (GE). Samples were bound to 1 mL HisTrap FF column, washed with 25 mM Tris-HCl pH 8.0, 300 mM NaCl, 1mM TCEP, 5% glycerol, 20 mM imidazole and eluted with a step elution over 20 CV of 25 mM Tris-HCl pH 8.0, 300 mM NaCl, 1 mM TCEP, 5% glycerol and 500 mM imidazole at a flow rate of 0.8 ml/min. This was followed by size-exclusion on a 16/60 S200 column (25 mM Tris-HCl pH 8.0, 300 mM NaCl, 1 mM TCEP, 5% glycerol) at a flow rate of 1 ml/min. Purified protein was analyzed by SDS-PAGE, western blotting and measurement of A260/A280 to estimate levels of contaminating nucleosides. Aliquoted protein was snap-frozen in liquid nitrogen and stored at −80°C.

In vitro RNA methylation assays

Recombinant METTL1/WDR4 (300 nM; Evotec) was incubated for 2 h with S-adenosylmethionine (15 μM) and oligonucleotide (1 μM) in a Tris-HCl pH 8.0 buffer (20 mM) supplemented with 1 mM DTT and 0.01% Triton X-100.

Mass spectrometry analysis of RNA nucleoside m7G modification

Nucleosides were prepared from enzyme-processed RNA by enzymatic digestion, using a cocktail of Benzonase (Merck), Phosphodiesterase 1 (Merck), and Antarctic Phosphatase (New England Biolabs) as described previously (van Delft et al., 2017). The reactions were filtered using an Amicon 30kDa MWCO spin-column (Merck) to remove protein and the filtrate was mixed with a 2x loading buffer containing 0.1% formic acid and an internal standard (^{13}C-labeled uridine generated from 645672-1MG Merck KGaA, previously treated with Antarctic Phosphatase). The samples were loaded onto an ACQUITY UPLC HSS T3 Column, 100 Å, 1.8 μm, 1 mm X 100 mm (Waters Corp., Milford, MA, USA) and resolved using a gradient of 2%–10% acetonitrile in 0.1% formic acid over 10 min. Mass spectrometric analysis was performed in positive ion mode on an Orbitrap QExactive HF (Thermo Fisher, Waltham, MA, USA) mass spectrometer. Standard dilutions of all experimental nucleosides were prepared and analyzed in parallel. There were three technical replicates of each sample and the analytical processing was performed using XCalibur Software (Thermo Fisher).

Mature miRNA isolation

Mature miRNA fraction was isolated from total RNA by gel extraction: RNA was denatured by incubation at 73°C for 3 min in 2X Urea Sample Buffer (Thermo Fisher) and run in a 10% TBE–urea precast polyacrylamide gel (Thermo Fisher) at 250V for 12 min. Gel was visualized by ethidium bromide staining and the region corresponding to the expected size of mature miRNAs was excised using a synthetic 20-nt RNA ruler. After breaking the gel, miRNAs were eluted in a sodium acetate buffer (0.3 M sodium acetate, pH 5.2, 5 mM EDTA, 0.1% sodium dodecylsulfate) by freeze-thawing once on dry ice and incubating at 4°C overnight. miRNAs were purified by ethanol precipitation.

Context-specific mass spectrometry analysis of miRNA m7G modification

Oligonucleotides were prepared from miRNA fraction using RNase A (Thermo Fisher) and chromatographically separated by ion pair reverse phase chromatography (200 mM Hexafluoroisopropanol [HFIP], 8.5 mM triethylamine [TEA] in water as eluent A, and 100 mM HFIP, 4.25 mM TEA in methanol as eluent B). The oligonucleotides were resolved by a gradient of 2.5% to 20% B at 200 nl/min on Acclaim PepMap C18 solid phase (Thermo Fisher) and characterized by negative ion tandem LC-MS in a hybrid quadrupole – orbitrap (QExactive HF, Thermo Fisher). Data were collected in data-dependent acquisition mode in pathfinding experiments before subsequent hybrid acquisition investigation. Full scan MS1 data were acquired between 700 and 3500 m/z and extracted ion chromatograms from these data were used for label-free quantification of oligonucleotides derived from *let-7*. MS2 data were collected in subsequent scan events for the same *let-7* oligonucleotides by targeted, multiplexed, data-independent acquisition on filtered precursor ion masses multiplexed from the double and triple charge states of unmodified and monomethylated AGGAGGU (m/z of 1180.158, 786.436, 1187.166 and 791.108, with a window of 3 m/z). Technical replicates of n = 3 were acquired, with MS2 ions matched with to within 5 ppm.

Preparation of naive and *in vitro* methylated [α-^{32}P]-pri-let-7e

Plasmid pcDNA3-pri-let-7e (Addgene #51380; Heo et al., 2008) linearized by digestion with XbaI (NEB) was used as a template for RNA *in vitro* transcription in the presence of [α-^{32}P]-GTP (Perkin Elmer) using TranscriptAid T7 High Yield Kit (Thermo Fisher). The resulting RNA (369bp) was denatured by incubation at 73°C for 3 min in 2X Urea Sample Buffer (Thermo Fisher) and run in a 6% TBE–urea precast polyacrylamide gel (Thermo Fisher). The band corresponding to the expected size of *pri-let-7e* transcript was excised using autoradiography and, after breaking the gel, RNA was eluted as described above for mature miRNA isolation.

2.5 μg of ^{32}P-labeled *pri-let-7e* (1 μM) were incubated for 3 h at 37°C in the presence of recombinant METTL1/WDR4 (300 nM) and S-adenosylmethionine (1 mM) in a methylation buffer (85 mM Tris-HCl pH 8.0, 1.4 mM DTT, 0.07 mM EDTA, 1 mM spermidine). Methylated *pri-let-7e* was isolated by immunoprecipitation using m7G-specific antibody, purified on RNA Clean & Concentrator - 5 columns (Zymo) and quantified by scintillation counting (Hidex 300 SL).

Isolation of DROSHA by IP

Immunopurification of DROSHA and *in vitro* processing assays were performed according to a published protocol (Lee et al., 2002). HEK293T cells transfected with pcDNA4/TO/cmycDrosha plasmid (Landthaler et al., 2004; Addgene plasmid #10828) were lysed after 48 h in buffer D (20 mM HEPES–KOH pH 7.9, 100 mM KCl, 0.2 mM EDTA, 0.5 mM DTT, 0.2 mM PMSF, 5% glycerol) by sonication (Bioruptor: 5 min; 30 s ON / 30 s OFF, 200W) followed by centrifugation. 2 mg of crude extract were incubated with 12 μg of anti-myc-tag antibody (Abcam) for 2 h at 4°C, then the recombinant enzyme was pulled-down with 30 μL Protein G Sepharose 4 Fast Flow beads (GE Healthcare) and washed 4 times in buffer D. Whole-cell extracts were prepared from control and *METTL1* knockdown A549 cells using the same lysis protocol as above.

In vitro miRNA Processing Assays

For comparing the pri- to pre-miRNA processing efficiency by DROSHA, 30000 cpm (50-100 ng) of either naive or *in vitro* methylated *pri-let-7e* were incubated with 15 μL of DROSHA-IP beads, 6.4 mM $MgCl_2$ and 1U/μl RNaseOUT RNase inhibitor (ThermoFisher) for 80 min at 37°C.

For evaluating the processing activity of METTL1 depleted cells, 10 μL of processing reaction containing 5 μL of whole-cell extract, 1 μL of solution A (32 mM $MgCl_2$, 5 mM ATP, 200 mM creatine phosphate), 1 μL 32 mM S-adenosyl methionine, 1 U/μl RNase inhibitor (Thermo Fisher) and the labeled transcripts (10^4-10^5 c.p.m.) were incubated at 37°C for 120 min.

All processing reactions were purified with RNA Clean & Concentrator - 5 column kit (Zymo), measured and equalized by scintillation counting, resolved on a 6% TBE–urea precast polyacrylamide gel (Thermo Fisher) and developed by autoradiography exposing the dried gel on Amersham Hyperfilm high-sensitivity film (GE Healthcare).

Circular dichroism spectroscopy

CD experiments were conducted on a Chirascan Plus spectropolarimeter. Oligonucleotide solutions were prepared at a final concentration of 10 μM (*rG4-let-7e* oligonucleotides) or 2.5 μM (*let-7e* hairpins) in 10 mM lithium cacodylate (pH 7.2) containing 1mM EDTA and supplemented with 100 mM of LiCl, NaCl or KCl. Oligonucleotides were annealed by heating at 95°C for 3 min and cooling the solutions at 4°C for 4 h. Scans were performed over the range of 200–320 nm at 5°C. Each trace was the result of the average of three scans taken with a step size of 1 nm, a time per point of 1 s and a bandwidth of 1 nm. A blank sample containing only buffer was treated in the same manner and subtracted from the collected data. The data were finally baseline corrected at 320 nm. Denaturation experiments were performed by heating the samples to 95°C using the stepped temperature ramping mode, a setting time of 10 s and with data collection every 1°C monitoring the CD signal at 263 and 210 nm. Differential melting curves (quantification of folded fractions) were obtained by subtracting the upper baseline to the signal and dividing by the difference between the upper and lower baseline. Melting temperatures ($T_{1/2}$) values were extracted as the local minima of the first derivatives of a Boltzman or bi-phasic dose-response fittings of the differential curves.

Polysome fractionation

Control or *METTL1* knockdown cells (n = 2) were treated 5 days after doxycycline induction with 0.1 mg/ml cycloheximide for 5 min at 37°C, then they were lysed and polysomes were fractionated on a sucrose gradient while measuring absorbance at 254 nm (Panda et al., 2017).

QUANTIFICATION AND STATISTICAL ANALYSIS

Bioinformatic analysis of smallRNA sequencing

Multiplexed reads were split on the basis of their barcodes using Illumina Basespace. Read quality was assessed using FastQC program (https://github.com/s-andrews/FastQC). Library adaptors were trimmed with Trimmomatic (Bolger et al., 2014), and reads were mapped to the human genome (NCBI GRCh38/hg38) with STAR (Dobin et al., 2013), using the parameters of ENCODE guidelines: *--runThreadN 15 --sjdbGTFfile /path/to/GENCODE_miRNA_subset.gtf --alignEndsType EndToEnd --outFilterMismatchNmax 1 --outFilterMultimapScoreRange 0 --outSAMtype BAM SortedByCoordinate --outFilterMultimapNmax 10 --outSAMunmapped Within --outFilterScoreMinOverLread 0 --outFilterMatchNminOverLread 0 --outFilterMatchNmin*

16 --alignSJDBoverhangMin 1000 --alignIntronMax 1 (Davis et al., 2018). Reads were summarized using featureCounts (Liao et al., 2014) according to miRbase22 annotation (Kozomara and Griffiths-Jones, 2014) of mature miRNAs. Differential miRNA enrichment in either m7G-RIP or BoRed-Pulldown over input/control sample was evaluated through negative binomial Wald test with the R package DESeq2 (Love et al., 2014; n = 2 for BoRed-Seq; n = 5 for m7G-RIP-Seq experiments).

Global gene expression analysis

Microarray spot analysis was performed with Feature Extraction software (Agilent Technologies). Data were background-corrected and quantile normalized among arrays using the Bioconductor package limma (Smyth and Speed, 2003; Smyth et al., 2005). The statistical significance of differential gene expression was calculated with the empirical Bayes method implemented in limma. KEGG pathway ontologies over-represented in the subset of genes upregulated or downregulated upon *METTL1* knockdown were evaluated using the R package Gage (Luo et al., 2009, 2017).

In order to identify the mRNAs that are predicted to be targets of selected miRNAs, we extracted the positive hits of at least 3 out of 6 *in silico* prediction algorithms (namely miRWalk, miRanda, miRDB, Pictar2, RNA22 and Targetscan) using miRWalk 2.0 web server (Dweep and Gretz, 2015).

For unbiased analysis of miRNA seed sequences enriched in the top upregulated genes upon *METTL1* knockdown, we took advantage of the Sylamer online software (van Dongen et al., 2008).

To produce a classification model predicting mRNA upregulation upon *METTL1* knockdown, we extracted the subset of genes expressed above the background (log_2 Average expression > 6). Genes displaying a log2FoldChange > 1 and a FDR < 0.05 were considered upregulated, and then a model employing the presence of putative miRNA target sites as predictors was generated by gradient boosting using R package gbm (Freund and Schapire, 1997).

Bioinformatic prediction of G-quadruplexes

Stem-loop and mature miRNA sequences were recovered from miRBase (Kozomara and Griffiths-Jones, 2014) release 22. These sequences were used to calculate the quantitative parameters used to describe the different miRNA features discussed in this manuscript. Base composition was assessed using a custom Python script. *G-score*, a quantitative estimation of G-richness and G-skewness, is based on the G4Hunter algorithm (Bedrat et al., 2016). Briefly, each position in a sequence is given a score between −4 and 4. To account for G-richness, a single G is given a score of 1, in a GG sequence each G is given a score of 2; in a GGG sequence each G is given a score of 3; and in a sequence of 4 or more Gs each G is given a score of 4. To account for G-skewness, Cs are scored similarly but their values are negative. The Gscore is the maximum value obtained while scanning miRNA sequences using a 20nt window and averaging the score of each nucleotide over the considered window.

G-quadruplex forming motifs, G2N7, G2N3, G3N7 and G3N12 are sequences of the form $G_{2+}N_{1-7}G_{2+}N_{1-7}G_{2+}N_{1-7}G_{2+}$, $G_{2+}N_{1-3}G_{2+}N_{1-3}G_{2+}N_{1-3}G_{2+}$, $G_{3+}N_{1-7}G_{3+}N_{1-7}G_{3+}N_{1-7}G_{3+}$ and $G_{3+}N_{1-12}G_{3+}N_{1-12}G_{3+}N_{1-12}G_{3+}$ respectively, where N is any base. G3N7 represents the strict definition of G4 forming sequences according to the Quadparser algorithm (Huppert and Balasubramanian, 2005). Other motifs represent the loose definition of G4 forming sequences (Kwok et al., 2016a). The presence of each motif within miRNA sequences was assessed using the *re.finditer* function in custom python scripts.

RNA secondary structures were predicted using the RNAfold 2.2.10 algorithm of the ViennaRNA package (Lorenz et al., 2011). RNAfold computes the minimum free energy (MFE) of optimal secondary structures via estimation of base pairing probabilities. MFEs of dsRNA secondary structures (ΔG^0_{dsRNA}) were computed at 37°C. MFEs of G4 secondary structures (ΔG^0_{G4}) were computed by subtracting MFEs obtained when considering G4 formation into the structure prediction algorithm to the previous values ($\Delta G^0_{G4} = \Delta G^0_{dsRNA} - \Delta G^0_{(dsRNA + G4)}$). RNAfold was used to assess the stability of predicted RNA structures within miRNA sequences using a 20nt sliding window. m7G-containing miRNAs are the subset of miRNAs that have been enriched from the total population of miRNAs using both the BoRed-Seq and m7G-RIP-Seq protocols. Background is the rest of annotated miRNAs. For assessing the local enrichment of structures within m7G-containing precursor miRNAs, pre-miRNA sequences were piled up and centered, MFE values were then averaged at each position. Data were compiled and plotted using R.

Statistical analysis

All general statistical analyses were performed using either a two-tailed Student's t test or a Wilcoxon test (when distributions were assessed not to be normal and homoscedastic) at a confidence interval of 95%, unless otherwise specified. No statistical methods were used to predetermine sample size.

DATA AND SOFTWARE AVAILABILITY

Data Resource

Raw genomic data have been deposited in the Gene Expression Omnibus database with accession number GSE112182 (Expression Microarray data: GSE112180; BoRed-seq and m7G-RIP-seq in A549: GSE112181; m7G-RIP-Seq in Caco-2: GSE120454; m7G-RIP-Seq in A549 *METTL1* knockdown: GSE120455). Unprocessed imaging data are deposited on Mendeley Data: (https://data.mendeley.com/datasets/yscng45zgj/1). All other data and analysis scripts are available from the corresponding author upon reasonable request.

Molecular Cell, Volume *74*

Supplemental Information

METTL1 Promotes *let-7* MicroRNA Processing via m7G Methylation

Luca Pandolfini, Isaia Barbieri, Andrew J. Bannister, Alan Hendrick, Byron Andrews, Natalie Webster, Pierre Murat, Pia Mach, Rossella Brandi, Samuel C. Robson, Valentina Migliori, Andrej Alendar, Mara d'Onofrio, Shankar Balasubramanian, and Tony Kouzarides

Figure S1

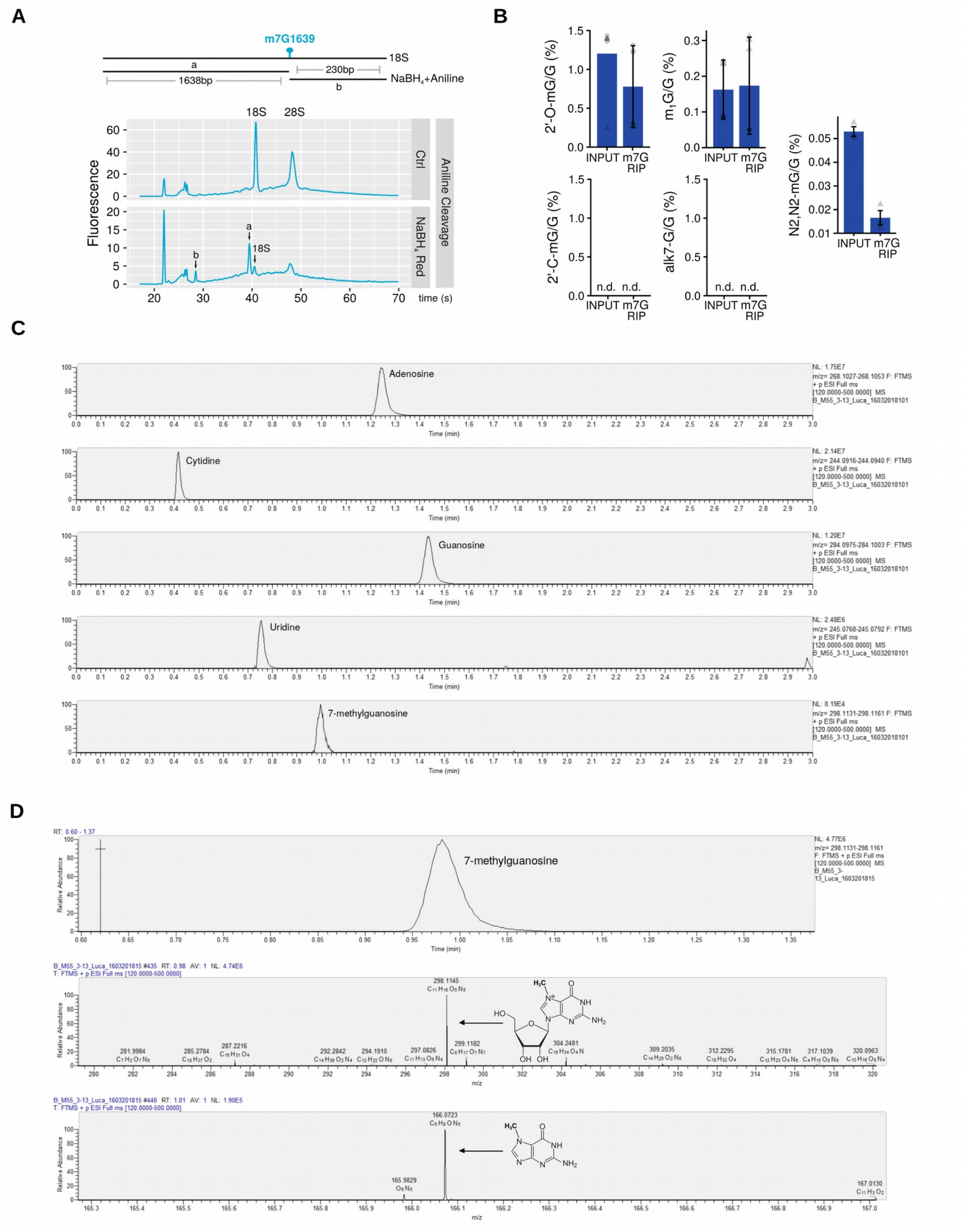

Figure S1 | High-throughput profiling of m7G modification in miRNAs. Related to Figure 1.
A, Schematic representation (top) of the expected cleavage of *18S* rRNA after $NaBH_4$ + aniline treatment and electropherogram analysis (bottom) of untreated and treated total RNA from A549 cells. **B,** Immunoprecipitation with anti-m7G antibody does not enrich RNAs containing five different guanine modifications (including four methylations) as determined by mass spectroscopy. Error bars represent S.E.M. **C,** HPLC trace of the four canonical RNA nucleosides along with 7-methylguanosine, showing retention times that are an exact match with pure nucleosides run as quantitation standards. **D,** Extracted ion chromatogram of 7-methylguanosine, with the accompanying mass spectra showing the intact protonated precursor ion of the nucleoside [M+H] (middle panel) and the 7-methylguanine ion generated by in-source fragmentation as an internal verification of its identity.

Figure S2

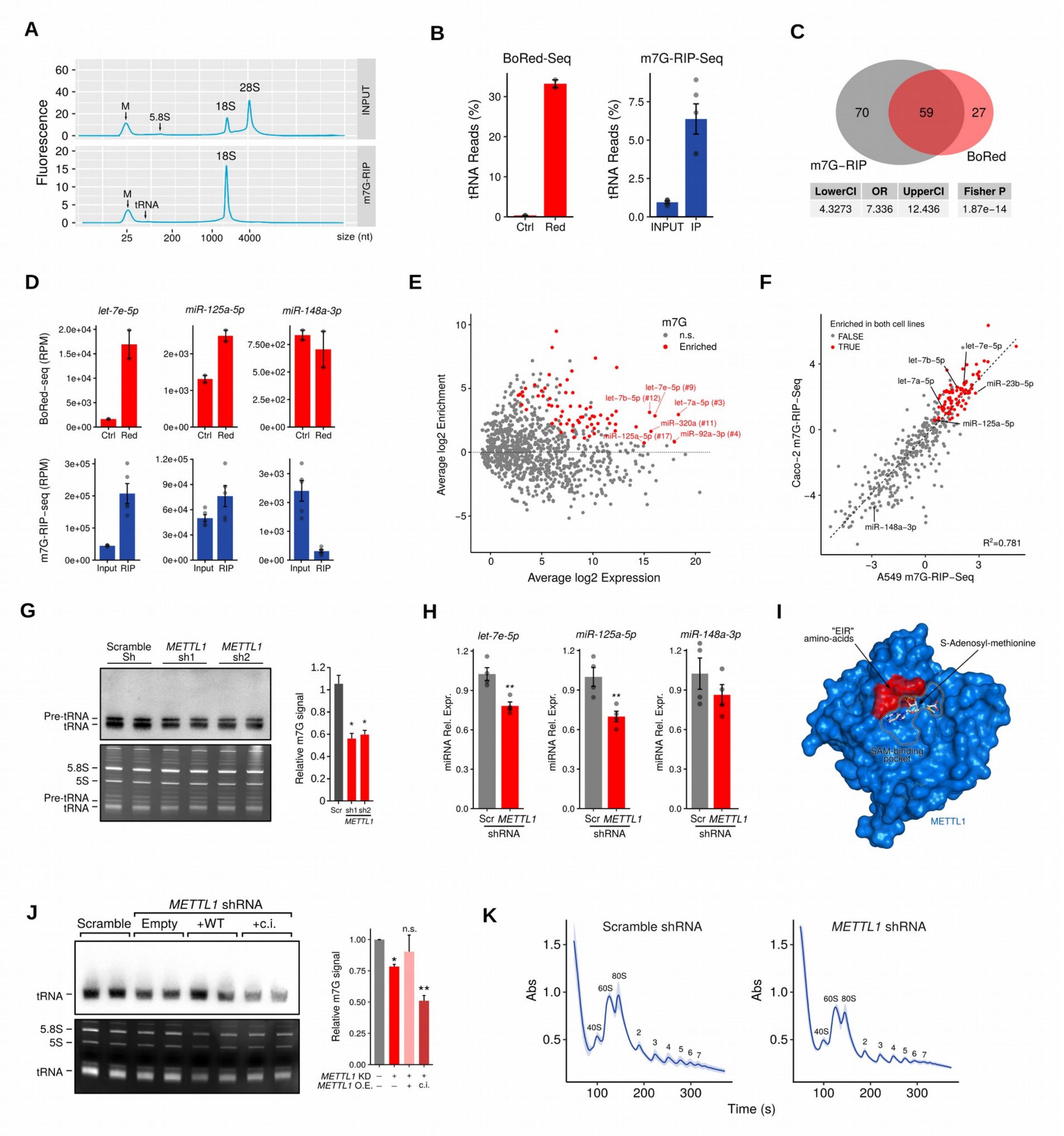

Figure S2 | Validation of BoRed-Seq and m7G-RIP-Seq. Related to Figure 1.
A, Electropherogram analyses of total RNA before and after immunoprecipitation with an m7G-specific antibody showing specific enrichment for *18S* rRNA in the immunoprecipitate. **B,** Read counts for tRNAs from the BoRed-Seq and m7G-RIP-Seq experiments are shown. Error bars represent S.E.M. **C,** Venn diagram showing the high overlap between miRNAs significantly enriched in either m7G-RIP-Seq or BoRed-Seq of A549 cells, respectively. Confidence interval of the odds ratio (OR) and P-value, as obtained by Fisher's exact test, are indicated in the table below. **D,** Read counts for the indicated miRNA from the BoRed-Seq and RIP-Seq experiments are shown. Error bars represent S.E.M. **E,** Scatter plot showing the absence of correlation between m7G and miRNA expression levels. Labels show the m7G containing miRNAs amongst the top 20 most expressed ones and their absolute ranking. **F,** Scatter plot of m7G-RIP-Seq miRNA enrichment in Caco-2 versus A549 cells, highlighting a high degree of correlation, as indicated by R^2 Pearson correlation coefficient. **G,** Immunoblot using an m7G-specific antibody shows a reduction of m7G levels in tRNAs upon *METTL1* knockdown. Right graph shows the average ± S.E.M. digital quantification of the m7G signal (*P<0.05, two tailed t-test). **H,** RT-qPCR quantification of *let-7e-5p, miR-125a-5p* and *miR-148a-3p* upon *METTL1* KD in Caco-2 cells. The average of four independent biological replicates ± S.D. is shown (**P<0.01, two tailed t-test). **I,** Crystal structure of METTL1 in complex with S-adenosylmethionine (PDB file: 3CKK). The EIR (107-109) amino acids mutated to alanine to generate the catalytically inactive mutant are shown in red. **J,** RNA immunoblot with an m7G-specific antibody on total RNA from *METTL1* knockdown or control cells over-expressing either WT METTL1 or a catalytically inactive METTL1 (c.i.). **K,** Polysome fractionation analysis. Cell extracts from control or *METTL1* knockdown cells were prepared and resolved in a 5–50% sucrose gradient. The absorbance at 254 nm was continuously measured. The peaks corresponding to free 40S and 60S subunits, 80S and polysomes are indicated. Shaded area in grey indicates the S.E.M. of two independent samples for each condition.

Figure S3

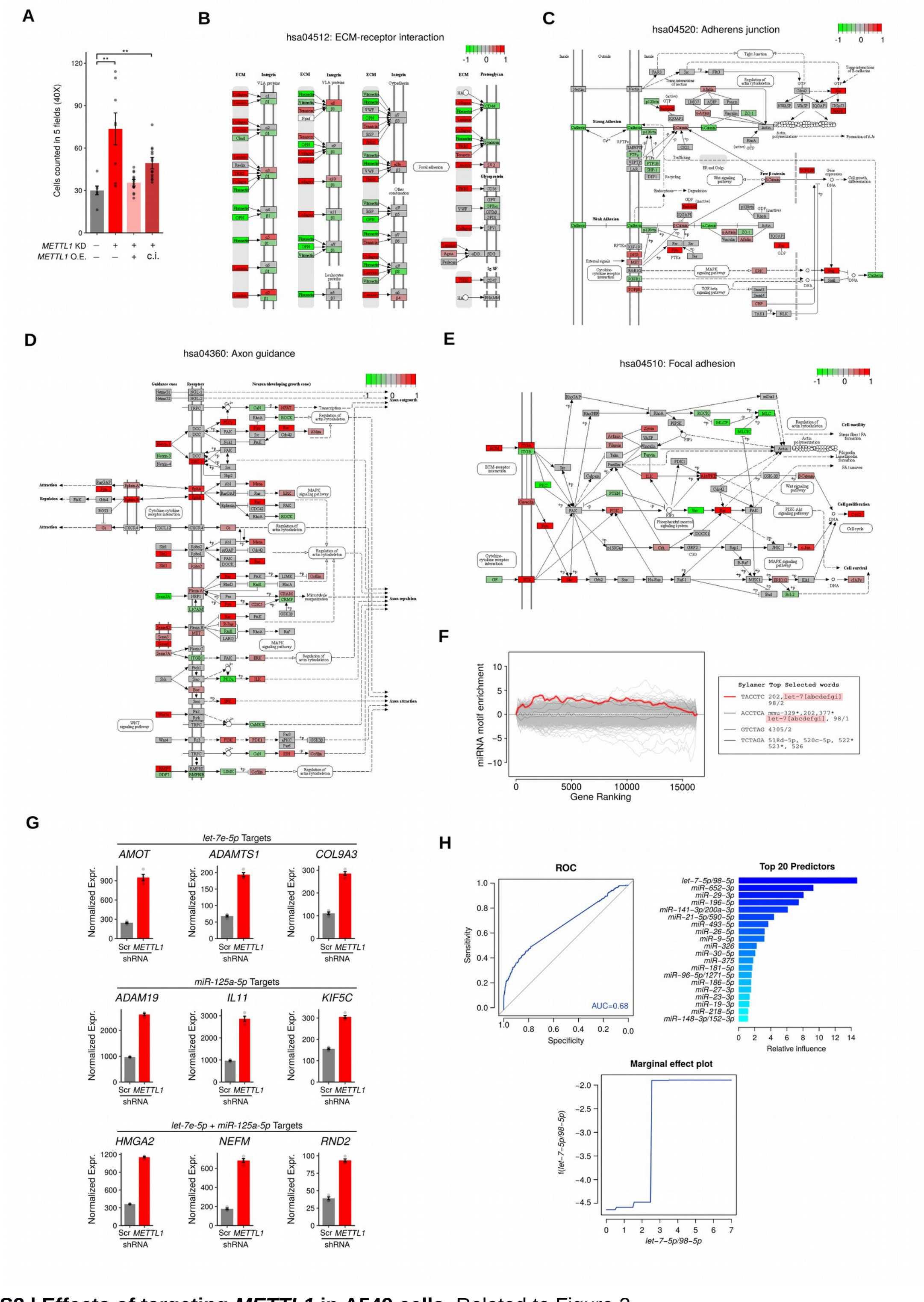

Figure S3 | Effects of targeting *METTL1* in A549 cells. Related to Figure 2.
A, migration assay of A549 cells. Increased migration due to *METTL1* KD is rescued by the expression of WT METTL1 (+), but not by a catalytically inactive version of the enzyme (c.i.). **B,C,D,E,** Graphical representation of KEGG pathway regulation, showing up-regulation of ECM-receptor interaction (B), adherens junction (C), axon guidance (D) and focal adhesion (E). Data were obtained by comparing RNA expression data from *METTL1* KD and control A549 cells (up-regulated genes, red; down-regulated genes, green). **F**, Sylamer miRNA target seed motif analysis on up-regulated mRNAs upon *METTL1* KD, showing a specific enrichment for the *let-7 (5p)* seed motif. **G,** representative cell migration genes whose expression is upregulated upon *METTL1* KD, which are targets of *let-7e-5p*, *miR-125a-5p* or both (for all of them FDR<0.05, as evaluated with *limma*). **H,** A gradient boosting classifier was used to model the mRNA expression fold changes observed by microarray analysis as a function of the presence of seed sites for different miRNAs in 3'-UTRs. Receiver operating characteristic (ROC) curve displays the goodness of the model, with an area under ROC (AUC) of 0.68. The presence of *let-7 (5p)* seed sequence within an mRNA represents the strongest predictive factor for its up-regulation upon *METTL1* KD, as shown by the top 20 predictor list. The marginal effect plot shows that there is a positive correlation between the number of seeds in the 3'-UTR and mRNA up-regulation.

Figure S4

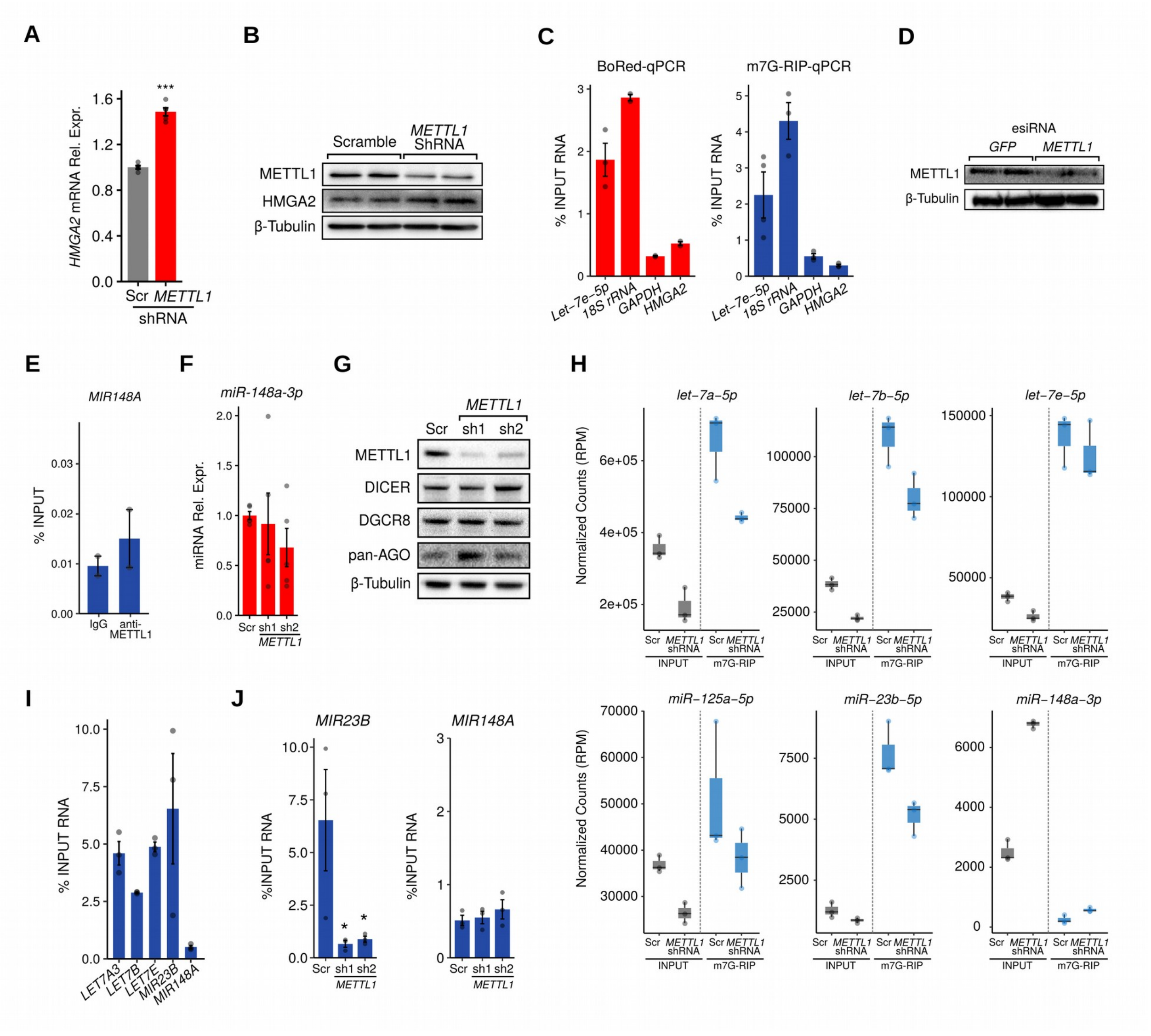

Figure S4 | miRNA processing is dependent on m7G. Related to Figure 3 and 4.
A, *HMGA2* expression measured by RT-qPCR in Caco-2 cells infected with *METTL1*-specific or control (Scr) TET-inducible shRNAs five days after doxycycline treatment. The average of five biological replicates ± S.D. is shown (***p<0.001, two tailed t-test). **B,** Western blot showing METTL1, HMGA2 and β-tubulin protein levels in Caco-2 cells infected with *METTL1*-specific or control (Scramble) TET-inducible shRNAs five days after doxycycline treatment. Two representative biological replicates of a total of four are shown. **C,** BoRed and m7G-RIP coupled to RT-qPCR of *HMGA2* in A549 cells. *18S* rRNA is used as a positive control; *GAPDH* is a negative control. *let-7e-5p* levels are replotted alongside as a positive reference. The average of three biological and technical replicates ± S.E.M. are shown. **D,** Western blot showing METTL1 down-regulation upon transfection with *METTL1* specific siRNAs in A549 cells stably expressing a luciferase cDNA with a *Hmga2*-3'-UTR with mutated *let-7* seed sites. Two independent transfections of a total of four replicates are shown. **E,** CLIP-qPCR using a METTL1-specific antibody or a non-specific IgG. The levels of immunoprecipitated *miR-148a* primary hairpin are shown. The average of two independent immunoprecipitation reactions ± S.E.M. is presented (*P<0.05, two tailed t-test). **F,** RT-qPCR showing the levels of *miR-148a-3p* upon *METTL1* KD in A549 cells. The average of five independent biological replicates ± S.D. is shown. **G,** Western blot of DICER, DGCR8 and ARGONAUTE (pan-AGO) upon *METTL1* KD in A549 cells. **H,** Box plots visualizing the normalized smallRNA-Seq counts of INPUT and m7G-RIP miRNAs from either control (Scr) or *METTL1* KD A549 cells. **I,** m7G RNA immunoprecipitation and RT-qPCR of *LET7A3*, *LET7B*, *LET7E/125A* and *MIR23B* primary transcripts in A549 cells. *MIR148A* is shown as a negative control. **J,** m7G-RIP and RT-qPCR of *MIR23B* and *MIR148A* primary transcripts upon *METTL1* KD in A549 cells. The average of three independent biological replicates ± S.E.M. is shown (*P<0.05, **P<0.01, ***P<0.001, two tailed t-test).

Figure S5

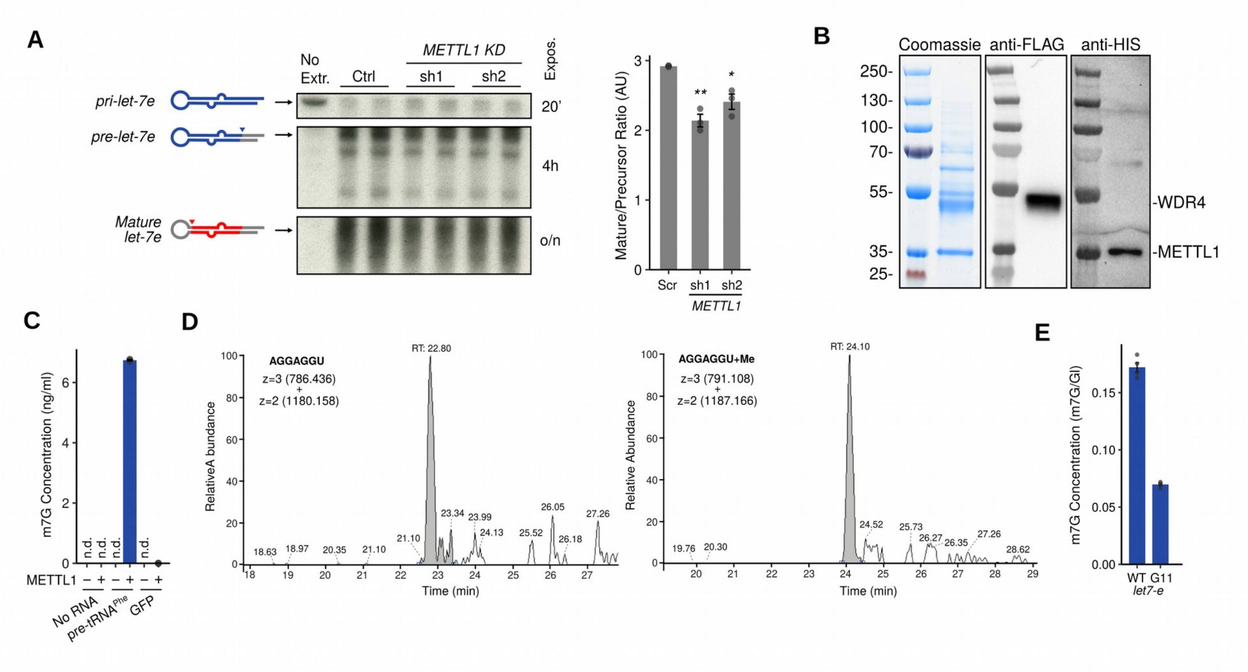

Figure S5 | METTL1-mediated miRNA methylation. Related to Figure 4 and 5.

A, Autoradiography and digital quantification of the results from *in vitro* processing of a radioactively labelled *pri-let-7e* incubated with cell extracts from control (Ctrl) or *METTL1* KD A549 cells. The average of three independent experiments ± S.D. is shown (*P<0.05, **P<0.01, two tailed t-test). Autoradiography images are composite of different molecular weight regions and exposure times. Full, unprocessed images are deposited on Mendeley Data. **B,** Coomassie staining and Western blot of co-expressed recombinant 6xHis-tagged METTL1 and FLAG-tagged WDR4 from baculovirus-infected insect cells. **C,** *In vitro* methylation reaction using recombinant METTL1/WDR4 pre-assembled complex on pre-$tRNA^{Phe}$ or a hairpin shRNA targeting GFP (negative control). Mass spectrometry analysis shows specific m7G methylation of pre-$tRNA^{Phe}$. **D,E,** Representative extracted ion chromatograms of collated charge states of unmodified (left chromatogram) and methylated AGGAGGU (right chromatogram) oligonucleotides. Precursor ion areas were quantified from technical triplicates (quantified area shown in grey). Chromatograms shown are from a single replicate. **E,** *in vitro* methylation reaction using recombinant METTL1/WDR4 pre-assembled complex on WT and G11-DAG *let-7* oligonucleotides.

Figure S6

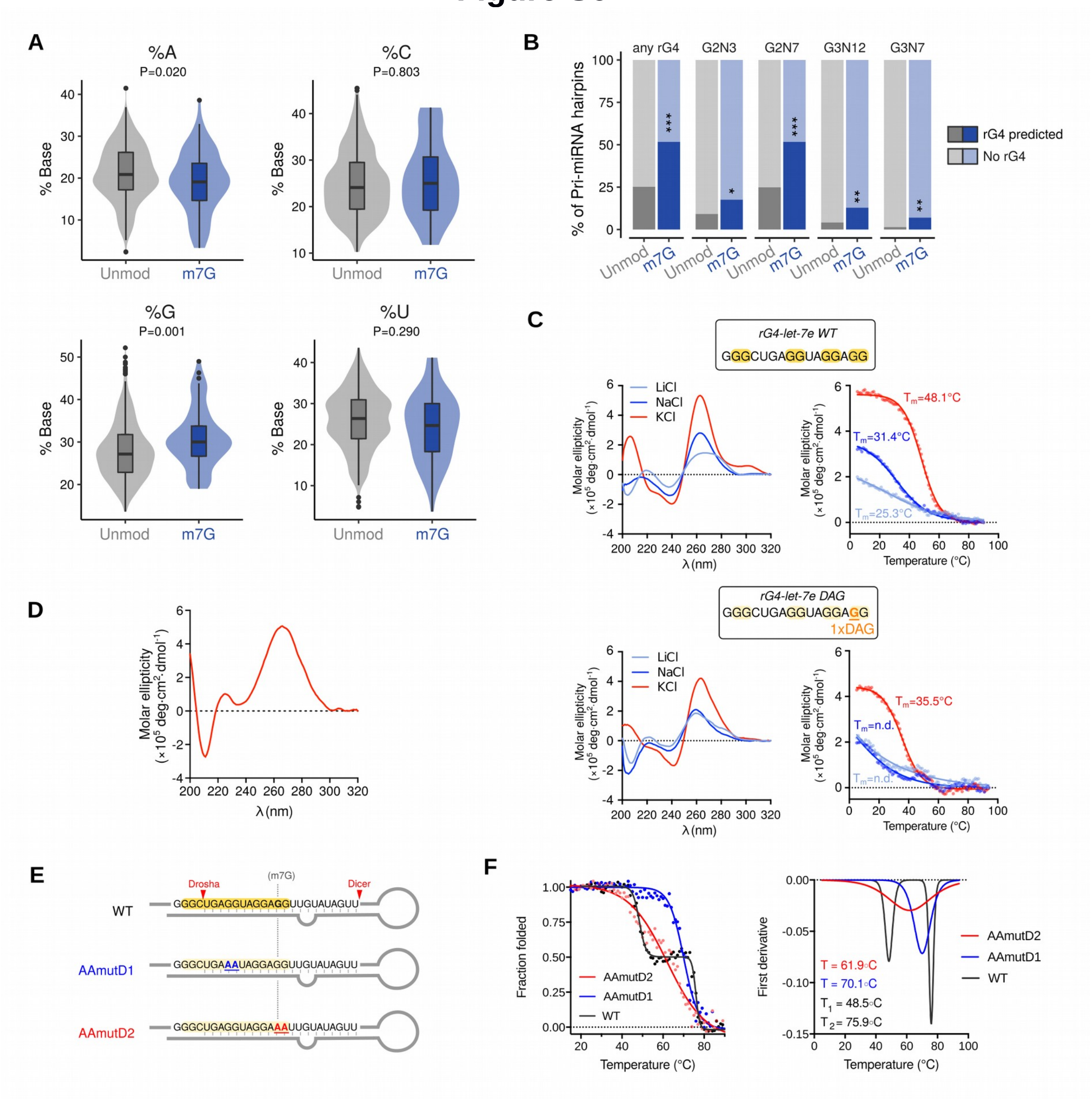

Figure S6 | m7G modified miRNAs are prone to G-quadruplex formation. Related to Figure 5.
A, Box plot showing the base content in primary hairpins of either unmodified or m7G containing miRNAs (P-values are calculated with Wilcoxon test). **B,** Proportion of G-quadruplex forming motifs detected within primary hairpins of either unmodified or m7G containing miRNAs (see STAR Methods for details; *P<0.05, **P<0.01, ***P<0.001; Fisher's exact test). **C,** Circular dichroism (CD) of the minimal *rG4-let-7e* sequence reveals that its structure is cation-dependent and its spectra display a minimum and maximum at 240 and 263 nm, respectively. These are feature of G-quadruplex formation. The stability of *rG4-let-7e* WT structure is cation-dependent, with a thermal stability (T_m) of 48.1 °C in the presence of 100 mM KCl. While G11 to DAG substitution in *rG4-let-7e DAG* does not completely abolishes rG4 formation, it greatly destabilizes it. The thermal stability decreases from 48.1 °C to 35.5 °C in the presence of 100 mM KCl and the oligonucleotide is no longer folded in the presence of LiCl or NaCl. **D,** CD spectra of *pri-let-7e* hairpin. The minimum at 210 nm indicates the expected stem-loop structure. **E,** Cartoon depicting the pri-miRNA hairpins employed in the following panel. **F,** Thermal denaturation studies of GG to AA mutants at the D1 or D2 position, supporting the formation of a G-quadruplex within *pri-let-7e*. Melting profiles of both mutants display a single transition at high temperature characteristic of the canonical hairpin structure. The transition attributed to the rG4 motif is no longer observed. Both mutations destabilize the hairpin structure as expected (decreased GC content).

Table S8

shRNA sequences	
METTL1 **sh1**	CCGGGATGACCCAAAGGATAAGAAACTCGAGTTTCTTATCCTTTGGGTCATCTTTTTG
METTL1 **sh2**	CCGGCCCACATTTCAAGCGGACAAACTCGAGTTTGTCCGCTTGAAATGTGGGTTTTTG
**Scramble shRNA**	CCGGCAACAAGATGAAGAGCACCAACTCGAGTTGGTGCTCTTCATCTTGTTGTTTTTG

Cloning Primers		
METTL1 **cDNA**	**Fw**	GTAGGCGGCCGCACCATGGCAGCCGAGACTCGGA
	**Rev**	CACTGTCTAGATCAGTGACCAGGCAGGCTGG

Gene Synthesis sequences	
METTL1 **WT**	CACACTGGCGGCCGCACCATGGCAGCCGAGACTCGGAACGTGGCCGGAGCAGAGGCCCCACCGCCCCAGAAGCGCTACTACCGGCAACGTGCTCACTCCAACCC<br>CATGGCGGACCACACGCTGCGCTACCCTGTGAAGCCAGAGGAGATGGACTGGTCTGAGCTATACCCAGAGTTCTTCGCTCCACTCACTCAAAATCAGAGCCACG<br>ACGATCCTAAAGACAAAAAAGAAAAGAGAGCTCAGGCCCAAGTGGAGTTTGCAGACATAGGCTGTGGCTATGGTGGCCTGTTAGTGGAACTGTCACCGCTGTTC<br>CCAGACACACTTATTCTGGGTCTGGAGATCCGGGTGAAGGTCTCAGACTATGTACAAGACCGGATTCGGGCCCTACGCGCAGCTCCTGCAGGTGGCTTCCAGAA<br>CATCGCCTGTCTCCGTAGCAATGCCATGAAGCACCTTCCTAACTTCTTCTACAAGGGCCAGCTGACAAAGATGTTCTTCCTCTTCCCCGATCCCCACTTTAAAC<br>GCACAAAGCACAAGTGGCGAATCATCAGTCCCACCCTGCTAGCAGAAT
METTL1 **EIR/AAA Mut**	CACACTGGCGGCCGCACCATGGCAGCCGAGACTCGGAACGTGGCCGGAGCAGAGGCCCCACCGCCCCAGAAGCGCTACTACCGGCAACGTGCTCACTCCAACCC<br>CATGGCGGACCACACGCTGCGCTACCCTGTGAAGCCAGAGGAGATGGACTGGTCTGAGCTATACCCAGAGTTCTTCGCTCCACTCACTCAAAATCAGAGCCACG<br>ACGATCCTAAAGACAAAAAAGAAAAGAGAGCTCAGGCCCAAGTGGAGTTTGCAGACATAGGCTGTGGCTATGGTGGCCTGTTAGTGGAACTGTCACCGCTGTTC<br>CCAGACACACTTATTCTGGGTCTGGCGGCAGCCGTGAAGGTCTCAGACTATGTACAAGACCGGATTCGGGCCCTACGCGCAGCTCCTGCAGGTGGCTTCCAGAA<br>CATCGCCTGTCTCCGTAGCAATGCCATGAAGCACCTTCCTAACTTCTTCTACAAGGGCCAGCTGACAAAGATGTTCTTCCTCTTCCCCGATCCCCACTTTAAAC<br>GCACAAAGCACAAGTGGCGAATCATCAGTCCCACCCTGCTAGCAGAAT

qPCR Taqman Primers

**Primer**		**Sequence**	**Universal ProbeLibrary Number (Roche Cat# 4683633001)**
GAPDH	**Fw**	TCCACTGGCGTCTTCACC	45
	**Rv**	GGCAGAGATGATGACCCTTTT	
HMGA2	**Fw**	ACCATTTCTGCAAGTTAGGTATGTT	2
	**Rv**	GAACAGGGAGAAAGTCAACTGC	
IL11	**Fw**	GGTGCATCTGTGGATAGAACG	28
	**Rv**	TCCTTAGCCTCCCTGAATGA	

qPCR Sybr Green Primers

**Primer**		**Sequence**
LET7A3	**Fw**	GGGTGAGGTAGTAGGTTGTATAG
	**Rv**	TATAGTTATCCCATAGCAGGGCAGA
LET7B	**Fw**	CGGGGTGAGGTAGTAGGTTGT
	**Rv**	CAGGGAAGGCAGTAGGTTGTATAG
LET7E/125A	**Fw**	GGGCTGAGGTAGGAGGTTGT
	**Rv**	GGGAAAGCTAGGAGGCCGTA
MIR23B	**Fw**	TGGCATGCTGATTTGTGACT
	**Rv**	GTGGTAATCCCTGGCAATGT
MIR148A	**Fw**	GGCAAAGTTCTGAGACACTCC
	**Rv**	TTCTGTAGTGCACTGACTTCTATCA
pre-let-7e	**Fw**	TGTATAGTTGAGGAGGACACCC
	**Rv**	GCTAGGAGGCCGTATAGTGA
pre-miR-125a	**Fw**	TCCCTGAGACCCTTTAACCT
	**Rv**	CAAGAACCTCACCTGTGACC
pre-miR-148a	**Fw**	GGCAAAGTTCTGAGACACTCC
	**Rv**	TTCTGTAGTGCACTGACTTCTATCA
RNY1	**Fw**	GCTGGTCCGAAGGTAGTGAG
	**Rv**	GGGGGAAAGAGTAGAACAAGG

qPCR Taqman Advanced miRNA Assay Ids (Thermo Fisher Cat# A25576)	
hsa-let-7e-5p	478579_mir
hsa-miR-125a-5p	477884_mir
hsa-miR-196a-5p	478230_mir
hsa-miR-375	478074_mir
hsa-miR-148a-3p	477814_mir
hsa-miR-381-3p	477816_mir
hsa-mir-101-3p	477863_mir

RNA Oligonucleotides	
pre-Phe-tRNA	GCGGAUUUAGCUCAGUUGGGAGAGCGCCAGACUGAAGAAAAAACUUCGGUCAAGUUAUCUGGAGGUCCUGUGUUCGAUCCACAGAAUUCGCACCA
cel-miR-67	CGUCGAUCCGCUCAUUCUGCCGGUUGUUAUGCUAUUAUCAGAUUAAGCAUCACAACCUCCUAGAAAGAGUAGAUCGAUU
pri-let-7e WT	CCCGGGCUGAGGUAGGAGGUUGUAUAGUUGAGGAGGACACCCAAGGAGAUCACUAUACGGCCUCCUAGCUUUCCCCAGG
pri-let-7e AAmutD1	CCCGGGCUGAAAUAGGAGGUUGUAUAGUUGAGGAGGACACCCAAGGAGAUCACUAUACGGCCUCCUAGCUUUCCCCAGG
pri-let-7e AAmutD2	CCCGGGCUGAGGUAGGAAAUUGUAUAGUUGAGGAGGACACCCAAGGAGAUCACUAUACGGCCUCCUAGCUUUCCCCAGG
pri-let-7e D1 (*=DAG)	CCCGGGCUGA**UAGGAGGUUGUAUAGUUGAGGAGGACACCCAAGGAGAUCACUAUACGGCCUCCUAGCUUUCCCCAGG
pri-let-7e D2 (*=DAG)	CCCGGGCUGAGGUAGGA**UUGUAUAGUUGAGGAGGACACCCAAGGAGAUCACUAUACGGCCUCCUAGCUUUCCCCAGG
pri-let-7e G11 (*=DAG)	CCCGGGCUGAGGUAGGA*GUUGUAUAGUUGAGGAGGACACCCAAGGAGAUCACUAUACGGCCUCCUAGCUUUCCCCAGG
rG4-let-7e WT	GGGCUGAGGUAGGAGG
rG4-let-7e DAG (*=DAG)	GGGCUGAGGUAGGA*G

Table S8 | DNA and RNA oligonucleotides employed in this study. Related to STAR Methods

Methods S1 – m7G Profiling Protocol

Related to STAR Methods

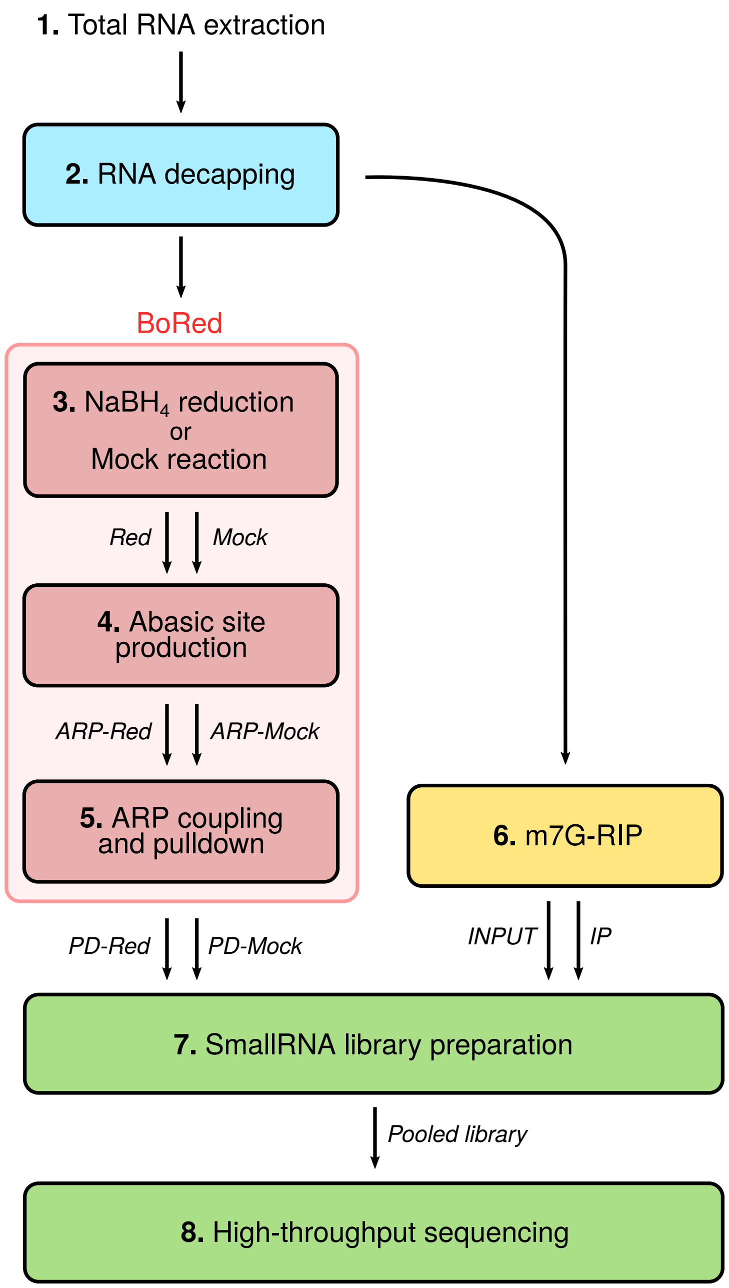

BoRed-Seq Protocol

1 RNA extraction from A549 cells

Total RNA, including miRNAs, is extracted from mammalian cells. To ensure optimal results, it is advisable to always start with freshly extracted RNA.

1. Culture up to 1.5×10^7 A549 cells to a confluency of ~80%.
2. Remove the medium and rinse the cells twice in PBS.
3. Extract RNA using the miRNeasy Mini Kit (Qiagen). Elute RNA in 30 µl RNAse-free water.

2 RNA decapping

Cap-Clip Acid Pyrophosphatase is used to remove the m7GpppG cap of eukaryotic mRNA in order to avoid cross-reactivity to non-internal m7G.

1. Prepare the RNA decapping mix according to Table 1.
2. Incubate for 1 h at 37 °C.
3. Purify RNA using the RNA Clean and Concentrator - 25 Kit (Zymo Research).

Table 1: Reaction mix for RNA decapping for ~20µg total RNA

Volume	Reagent
20 µg	RNA
2 µl	10× Reaction buffer
1 µl	CapCLIP (5 U; CellScript)
1 µl	RNAsin Plus (Thermo Fisher)
20 µl	Final vol. with ddH_2O

3 $NaBH_4$ Reduction

m7G residues are specifically reduced by treatment with $NaBH_4$ in the presence of high concentrations of free m7GTP. Control samples are treated with acid-quenched $NaBH_4$. The $NaBH_4$ treatment also reduces aldehydes in RNA (e.g. 5-formylcytosine or 5-formyluracil), thereby preventing unwanted covalent binding of ARP with them in the downstream stages.

1. Throughout the entire protocol, always use nuclease-free not DEPC-treated water (Ambion) as even small traces of DEPC lead to RNA degradation after $NaBH_4$ reduction.
2. Prepare 3 M Tris-HCl (pH 8.2) and 1 M sodium acetate (NaOAc; pH 4.5). Filter through 0.22 µm filter and autoclave. Store aliquots at -20 °C.
3. Prepare the RNA mix according to Table 2. For each sample prepare n×2 reaction mixes: one reduction reaction (red) and one control reaction (ctrl). At least three parallel reactions are recommended to obtain sufficient reduced RNA for subsequent steps.
4. Prepare a fresh 1 M sodium borohydride solution ($NaBH_4$; Sigma-Aldrich) immediately before use. The $NaBH_4$ powder should be stored at 4 °C in a desiccator.

Table 2: Reaction mix for 4 µg of RNA

Volume	Reagent
up to 4 µg	RNA
3 µl	37 mM m7GTP (Sigma-Aldrich)
5 µl	3 M Tris-HCl (pH 8.2)
15 µl	Final vol. with ddH_2O

5. Add 15 µl 1 M $NaBH_4$ solution to the reduction reaction. Vortex and spin briefly. Do not add $NaBH_4$ to the CTRL reaction and keep it on ice until quenching.
6. Incubate on ice for 40 min. Open the lid of the tube, debubble and spin down every 10 min.
7. Meanwhile prepare the quenched $NaBH_4$ solution (QB1) for the control reaction (non-reduced RNA) according to Table 3.
8. Incubate QB1 at 90 °C for 30 min.
9. Place QB1 on ice and add 1 µl of glycogen (20 µg/µl; Roche).

Table 3: Preparation of QB1 solution

Volume	Reagent
180 µl	1 M NaOAc (pH 4.5)
120 µl	ddH_2O
15 µl	1 M $NaBH_4$

Table 4: Preparation of QB2 solution

Volume	Reagent
180 µl	1 M NaOAc (pH 4.5)
120 µl	ddH_2O
1 µl	20µg/µl glycogen

10. Prepare the quenching solution for the reduction reaction (QB2) according to Table 4. Keep it on ice until used.
11. After the incubation of the reduction reaction (RED) on ice for 40 min, add 300 µl of QB2 quenching solution drop-wise, to avoid a violent reaction.
12. Add 990 µl of ice-cold absolute ethanol.
13. Add 315 µl of QB1 solution and 990 µl of ice-cold absolute ethanol to the control reaction (CTRL).
14. Incubate both reactions (RED and CTRL) for 1 h at -20 °C to precipitate RNA.
15. Centrifuge at ≥15,000 g for 30 min at 4 °C. Carefully decant the supernatant.
16. Wash the RNA with 1 ml of 70% ethanol. Vortex to detach the pellet for 5 min at room temperature.
17. Centrifuge at ≥15,000 g for 5 min at 4 °C.
18. Carefully remove the supernatant and air-dry the pellet.
19. Dissolve the pellet in 10 µl ddH_2O and quantify by Qubit RNA HS Assay (Thermo Fisher).

4 RNA abasic site production

The reduced m7G residues are subjected to nucleoside hydrolysis at low pH.

1. Prepare a sodium acetate solution (pH 3.9) by mixing 3.5 ml ddH_2O, 1.5 ml acetic acid and

300 µl 10 N sodium hydroxide (NaOH).

2. Add 1 µl 7-methyl-GTP (37 mM; Sigma-Aldrich) and 11 µl of acetate solution to the RNA dissolved in 10 µl of ddH_2O (ctrl or red).
3. Incubate the mix for 15 min at 37 °C.
4. Precipitate by adding 200 µl ddH_2O, 20 µl NaOAc (pH 4.5), 660 µl of ice-cold absolute ethanol and 1 µl glycogen.
5. Incubate for 1 h at -20 °C.
6. Centrifuge at ≥15,000 g for 30 min at 4 °C. Carefully decant the supernatant.
7. Wash the RNA with 1 ml of 70% ethanol. Vortex to detach the pellet.
8. Centrifuge at ≥15,000 g for 5 min at 4 °C.
9. Carefully remove the supernatant and air-dry the pellet.
10. Dissolve the pellet in 10 µl ddH_2O and quantify by Qubit RNA HS Assay.

5 ARP coupling and pulldown

A biotin-coupled aldehyde reactive probe covalently binds to the abasic RNA sites resulting from m7G reduction and hydrolysis. This allows pull-down with streptavidin-conjugated beads.

1. Prepare a 22.5 mM stock solution (4.5×) of Aldehyde Reactive Probe (ARP) by adding 1 ml ddH_2O to 10 mg of ARP powder (N-(aminooxyacetyl)-N'-(D-Biotinoyl) hydrazine, Trifluoroacetic Acid Salt; Thermo Fisher).
2. Prepare the 10× Reaction buffer containing 50 mM Tris-HCl (pH 6.8) and 10 mM EDTA.
3. Denature the RNA at 80 °C for 2 min.
4. Incubate RNA on ice for 5 min.
5. Prepare the ARP reaction according to Table 5.

Table 5: Preparation of ARP reaction mix

Volume	Reagent
4.5 µg	RNA (crtl or red)
8 µl	22.5 mM ARP (3 mM final)
6 µl	10× Reaction buffer
60 µl	Final vol. with ddH_2O

Table 6: Preparation of pulldown reaction

Volume	Reagent
2-3 µg	total RNA
200 µl	5× PD buffer
1 ml	Final vol. with ddH_2O

6. Incubate for 1 h at 30 °C.
7. Meanwhile prepare 1.33 M formaldehyde solution in water (dilution of 1:10 from 37% formaldehyde stock; Sigma-Aldrich).
8. To stop the reaction, add 2.25 µl 1.33 M formaldehyde solution, then add 40 µl ddH_2O, 10 µl 3 M sodium acetate and 300 µl absolute ethanol to the ARP samples.
9. Precipitate RNA at -20 °C for 1 h.
10. Pellet RNA by centrifugation at ≥15,000 g for 30 min at 4 °C.
11. Decant supernatant carefully and wash the pellet twice with 70% ethanol (step 7 of section 4).
12. Dissolve RNA in 50 µl ddH_2O.

13. Filter through an Illustra MicroSpin G-25 spin column (GE Healthcare) to remove the remaining unreacted ARP.
14. Prepare the 5× Pulldown (PD) buffer containing 50 mM Tris-HCl (pH 7.4), 750 mM sodium chloride (NaCl) and 0.5% NP-40 (v/v).
15. Wash 50 µl of Dynabeads MyOne C1 Streptavidin beads (Thermo Fisher) three times with 1× PD buffer.
16. Resuspend the beads in 1 ml 1× PD buffer + 0.5 mg/ml BSA (NEB).
17. Incubate for 1 h at 4 °C in rotation.
18. Prepare the PD reaction according to Table 6.
19. Add the blocked beads to the PD reaction.
20. Incubate at room temperature for 25 min in rotation.
21. Wash four times with ice-cold 1× PD buffer.
22. Add 700 µl QIAzol (Qiagen) and 140 µl chloroform to the beads.
23. Purify using the RNA Clean and Concentrator - 25 Kit (Zymo Research) according to the manufacturer's instructions.
24. Elute in 10-15 µl ddH_2O and quantify by Qubit RNA HS Assay.

6 7-Methylguanosine RNA Immunoprecipitation

Heat-denatured RNA is immunoprecipitated with a specific antibody against m7G. Both Input and IP samples are then purified using the same strategy in order to avoid any bias.

1. Denature decapped RNA for 2 min at 80 °C and place immediately on ice.
2. Save a 1-2.5 µg aliquot of RNA as INPUT control.
3. Prepare the 5× RIP buffer according to Table 7.
4. Prepare the RIP reaction according to Table 8.
5. Incubate the RIP reaction for 2 h at 4 °C in rotation.

Table 7: Preparation of 5× RIP reaction buffer

Volume	Reagent
0.5 ml	1 M Tris-HCl (pH 7.4)
1.5 ml	5 M NaCl
0.5 ml	10% NP-40 (v/v)
7.5 ml	ddH_2O

Table 8: Preparation of RIP reaction

Volume	Reagent
25 µg	total RNA
10 µg	anti-m7G antibody (RNO17M)
10 µl	RNAseOUT (Thermo Fisher)
200 µl	5× RIP buffer
1 ml	Final vol. with ddH_2O

6. Wash n×80 µl Dynabeads G (Thermo Fisher) twice with 1 ml 1× RIP buffer.
7. To block the Dynabeads, resuspend them in 1 ml 1× RIP buffer and add 50 µl 20 µg/µl BSA (NEB), then incubate for 2 h at 4 °C in rotation.
8. Wash the beads twice with 1× RIP buffer and resuspend them in n×100 µl 1× RIP buffer.
9. Add 100 µl beads to each RIP reaction.
10. Incubate for 2 h at 4 °C in rotation.

11. Wash three times with 1 ml ice-cold 1× RIP buffer, changing the 1.5 ml tube in the first wash.
12. Resuspend the beads in 100 µl 1× RIP buffer + 2 µl RNAseOUT (Thermo Fisher) + 6.7 mM 7-methyl-GTP (Sigma-Aldrich).
13. Incubate for 30 min at 37 °C to specifically elute m7G-harbouring RNAs, then collect the supernatant.
14. Bring the volume of the INPUT RNA control to 100 µl in 1× RIP buffer + 2 µl RNAseOUT + 6.7 mM 7-methyl-GTP.
15. Purify both INPUT and RIP samples with the RNA Clean and Concentrator - 25 Kit.
16. Elute in 10-15 µl ddH_2O and quantify by Qubit RNA HS Assay.

7 SmallRNA Sequencing Library Preparation

Sequencing libraries are prepared from resulting BoRed or m7G-RIP RNA with a commercial kit optimized to generate size-selected Small RNA libraries.

Prepare high-throughput libraries using NEBNext Small RNA Library Preparation kit for Illumina (NEB) starting from 1 µg of RNA for each sample and employing gel extraction as the final purification step, according to the manufacturer's protocol.

8 High Throughput Sequencing

Multiplexed libraries are sequenced by high-throughput sequencing-by-synthesis (Illumina).

Sequence the pooled libraries on Illumina platform running in 50-bp single-read mode. Plan the multiplexing strategy in order to get at least 15-20M raw reads per sample for BoRed-Seq and 5-10M reads for m7G-RIP.
